# Validation of automated lobe segmentation on paired inspiratory-expiratory chest CT in 8-14 year-old children with cystic fibrosis

**DOI:** 10.1371/journal.pone.0194557

**Published:** 2018-04-09

**Authors:** Philip Konietzke, Oliver Weinheimer, Mark O. Wielpütz, Dasha Savage, Tiglath Ziyeh, Christin Tu, Beverly Newman, Craig J. Galbán, Marcus A. Mall, Hans-Ulrich Kauczor, Terry E. Robinson

**Affiliations:** 1 Department of Diagnostic and Interventional Radiology, University Hospital of Heidelberg, Heidelberg, Baden-Württemberg, Germany; 2 Translational Lung Research Center Heidelberg (TLRC), German Center for Lung Research (DZL), University of Heidelberg, Heidelberg, Baden-Württemberg, Germany; 3 Department of Diagnostic and Interventional Radiology with Nuclear Medicine, Thoraxklinik at University of Heidelberg, Heidelberg, Baden-Württemberg, Germany; 4 Department of Radiology, University Hospital Kobe, Kobe, Hyogo Prefecture, Japan; 5 Center of Excellence in Pediatric Cystic Fibrosis and Pulmonary Diseases, Department of Pediatrics, Stanford University Medical Center, Stanford, California, United States of America; 6 Pediatric Radiology, Stanford University Medical Center, Stanford, California, United States of America; 7 Center for Molecular Imaging, Department of Radiology, University of Michigan, Ann Arbor, United States of America; 8 Division of Pediatric Pulmonology and Allergy and Cystic Fibrosis Center, Department of Pediatrics, University of Heidelberg, Heidelberg, Baden-Württemberg, Germany; 9 Department of Translational Pulmonology, University of Heidelberg, Heidelberg, Baden-Württemberg, Germany; Vanderbilt University Medical Center, UNITED STATES

## Abstract

**Objectives:**

Densitometry on paired inspiratory and expiratory multidetector computed tomography (MDCT) for the quantification of air trapping is an important approach to assess functional changes in airways diseases such as cystic fibrosis (CF). For a regional analysis of functional deficits, an accurate lobe segmentation algorithm applicable to inspiratory and expiratory scans is beneficial.

**Materials and methods:**

We developed a fully automated lobe segmentation algorithm, and subsequently validated automatically generated lobe masks (ALM) against manually corrected lobe masks (MLM). Paired inspiratory and expiratory CTs from 16 children with CF (mean age 11.1±2.4) acquired at 4 time-points (baseline, 3mon, 12mon, 24mon) with 2 kernels (B30f, B60f) were segmented, resulting in 256 ALM. After manual correction spatial overlap (Dice index) and mean differences in lung volume and air trapping were calculated for ALM vs. MLM.

**Results:**

The mean overlap calculated with Dice index between ALM and MLM was 0.98±0.02 on inspiratory, and 0.86±0.07 on expiratory CT. If 6 lobes were segmented (lingula treated as separate lobe), the mean overlap was 0.97±0.02 on inspiratory, and 0.83±0.08 on expiratory CT. The mean differences in lobar volumes calculated in accordance with the approach of Bland and Altman were generally low, ranging on inspiratory CT from 5.7±52.23cm^3^ for the right upper lobe to 17.41±14.92cm^3^ for the right lower lobe. Higher differences were noted on expiratory CT. The mean differences for air trapping were even lower, ranging from 0±0.01 for the right upper lobe to 0.03±0.03 for the left lower lobe.

**Conclusions:**

Automatic lobe segmentation delivers excellent results for inspiratory and good results for expiratory CT. It may become an important component for lobe-based quantification of functional deficits in cystic fibrosis lung disease, reducing necessity for user-interaction in CT post-processing.

## Introduction

Quantitative computed tomography (QCT) of cystic fibrosis (CF) lung disease is becoming an increasingly recognized and viable approach for evaluation of CF airway disease. Chronic progressive lung disease continues to determine more than 90% of morbidity and mortality in patients with CF [1–3]. Recent studies employing computed tomography (CT) in infants and preschool children with CF, including patients who were diagnosed by newborn screening, have confirmed early and progressive structural abnormalities of the lung, frequently observed even in the absence of respiratory symptoms [4–11]. Apart from irreversible structural lung damage in the form of bronchiectasis, potentially reversible and closely linked abnormalities such as mucus plugging, air trapping and perfusion abnormalities have increasingly drawn attention [12–14], and especially air trapping and perfusion abnormalities are considered early lesions that are present already in newborns and infants [9, 10, 14]. Only few studies have been conducted to longitudinally study the functional deficits, and it is hypothesized that visual scoring may not be sensitive enough to describe the exact spatial and temporal changes in air trapping. Thus, a more direct approach to QCT by computational post-processing is desirable [15, 16]. Semi-quantitative approaches have been proposed [17], but more complete direct computational airway dimension and air trapping quantification have also recently been described [12, 16–20]. QCT of air trapping has been challenging because it involves the segmentation of the lung volume on inspiratory and expiratory CT in order to detect density differences [15, 21, 22], and deformable registration for pixel-wise comparison has also been suggested [23]. Until now, a lobe-based quantification of air trapping has been hampered by the necessity to manually segment lung lobes from thin-slice high-resolution datasets on both acquisitions, which is cumbersome and limits the processing of a higher volume of patients. Robust automatic segmentation of lung lobes is now available for inspiratory CT of adults [24, 25] but not of school-age children with cystic fibrosis. Further, CT of young children and especially expiratory CT are specifically difficult to segment by such algorithms due to a much higher attenuation of the lung parenchyma itself with lower contrast to soft tissue chest wall and mediastinal structures as well as a reduction of airway and vessel calibers. Despite these difficulties a lobe-based assessment of CF lung disease may have an additional benefit, since CF shows very high heterogeneity between patients, as well as variable extent of disease from one time of assessment to another. Spirometry, yet the most important marker of disease severity and prognosis, delivers several quantitative markers on patient condition [26], but only globally for the entire lung. QCT on the other hand has the potential for regional assessment of disease burden, differentiation of CF-related imaging findings like bronchiectasis, mucus plugging and air trapping and may quantify the individual contribution of such findings to the overall disease burden. Pathologies affecting individual lobes are already taken into account in some clinical studies. For example in exacerbation, single lobes may be affected by consolidations, adding to the definition for pulmonary exacerbation [27], or the CT score was used to define a lobe with “greatest disease” to follow-up treatment response specifically in this lobe [4]. Especially changes in air trapping may just as well be related to the clinical course of a patient, and although there is no data specifically supporting a regional assessment of air trapping, there is no reason to regard air trapping differently than other imaging findings in CF. With this study, we propose a novel method for automatic lobe segmentation on inspiratory and expiratory CT as a preparatory study for lobe-based quantification of air trapping, and validate this method in a group of 16 school-age children with CF, each contributing a series of 4 CT scans, resulting in a total of 128 datasets.

## Materials and methods

### Ethics statement

The prospective multi-center study was carried out in 36 subjects enrolled in the Novartis CF Natural History Study [28] from 2007–2011 and was approved by the Institutional Review Boards of Stanford University Medical Center and Ohio State University School of Medicine. Informed written consent for examination and further data processing was obtained from all patients or legal guardians prior to inclusion.

### Patient population

16 children with confirmed CF at baseline were included in the study who were subjects in a joint Novartis Pharmaceutical—Cystic Fibrosis Foundation Therapeutics Development Network Consortium were included for this analysis. These 16 children were selected out of 36 subjects because they were examined exactly with the same scanner in the same institution. Moreover, they differed neither in the severity of the disease nor in any other aspect from the remaining 20 participants in the joint Novartis Pharmaceutical—Cystic Fibrosis Foundation Therapeutics Development Network Consortium study. Demographic data is reported in Table 1.

**Table 1:** Patients baseline characteristics.

Number of subjects	16
Age (years)	11.1±2.4
Male/female	9/7
Weight (kg)	39.2±10.3
Height (cm)	144±14.2
BMI (kg/m^2^)	18.5±2.2
FEV1 (l/s)	2.27±0.61
FEV1 (%)	106±11.9
TLC (l)	3.65±0.92
TLC (%)	108±11.2
RV (l)	0.88±0.35
RV (%)	131±38.4
RV/TLC (%)	24±5.3
PA positive	2
MRSA positive	3
Pancreatic insufficiency	16
Homozygous F508del	12

BMI = body mass index, FEV1 = forced expiratory volume in 1s, TLC = total lung capacity, RV = residual volume, RV/TLC = percentage of residual volume/total lung capacity. Percentage values refer to the predicted volumes. PA = chronic Pseudomonas aeruginosa infection; MRSA = chronic methicillin resistant Staphylococcus aureus infection.

### Multidetector computed tomography

All patients underwent multidetector CT (Siemens Sensation 64 CT scanner [32 detector], Siemens Medical Solutions, Malvern, PA) at baseline, and consecutively after 3, 12 and 24 months, comprising a total of 64 exams, i.e. 128 volumetric datasets. Exclusively non-enhanced spirometer-controlled paired inspiratory (100kVp, 30–50mAs; Pitch 1.0) and expiratory (100kVp, 30–50mAs; Pitch 1.2) CT was routinely performed in supine position as reported previously [15, 29, 30]. Specifically, spirometer control was employed to achieve full inspiration, and maximum expiration to near residual volume. The total estimated effective dose for the 4 serial CT scans over the 2 year period was 5.4–5.6 mSv. Reconstruction was performed in a medium soft B30f, as recommended for parenchyma quantification, as well as a sharp B60f algorithm [16, 20, 30]. All examinations were visually inspected by a reader with more than 20 years (BN) of experience in pediatric chest imaging for adequate inspiration, absence of significant motion artifacts and inclusion of all parts of the chest. The examination protocol and equipment were kept exactly constant during the study period.

### Automatic lobe segmentation

**Fig 1:**
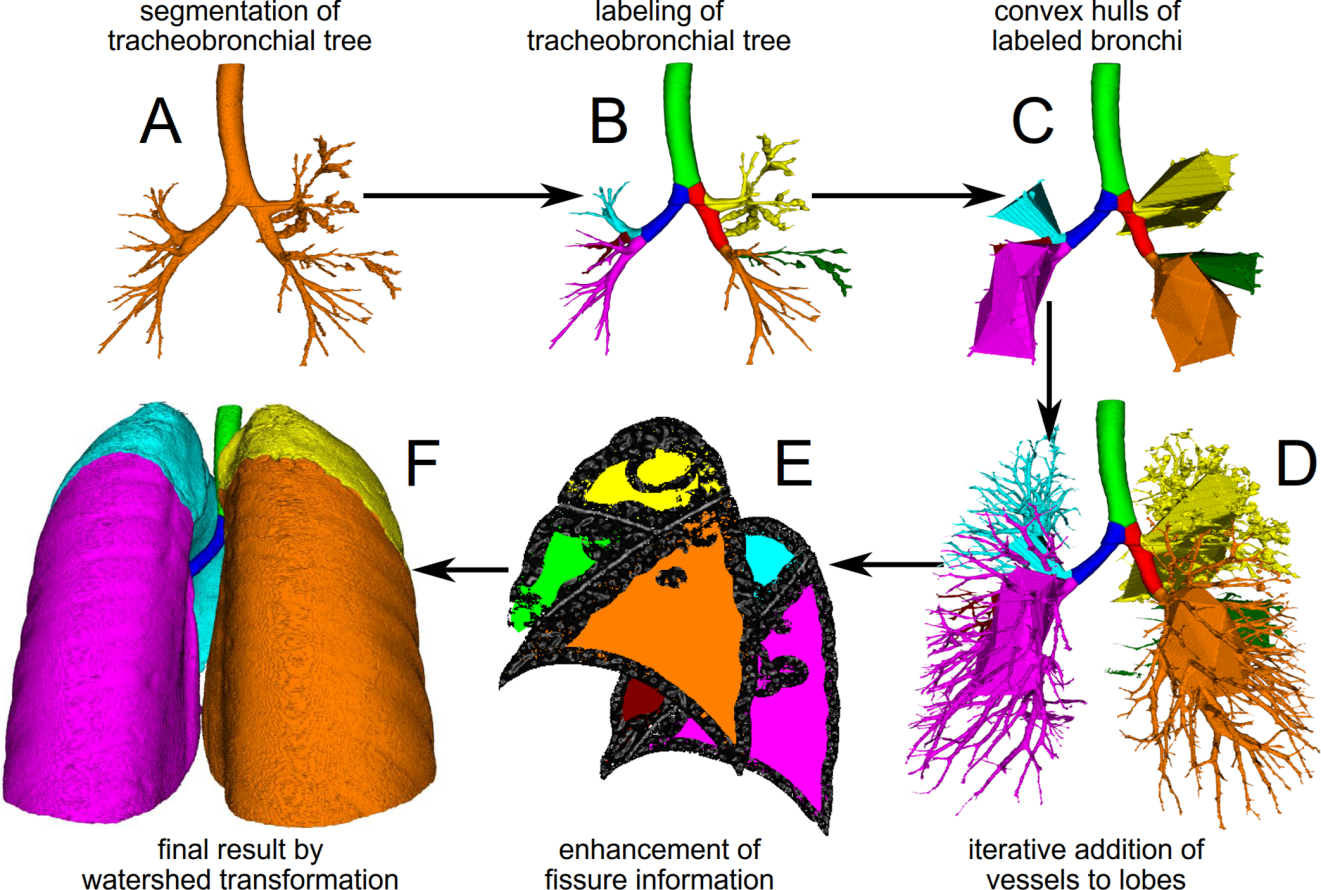
Work-flow chart for fully automatic lung lobe segmentation. A: The initial step is the segmentation of the airway tree. B: Second, central airways and lobar bronchi are labeled by an anatomical knowledge-based algorithm. C: Then, a convex hull around the labeled lobar bronchi is generated. D: In the next step vasculature is iteratively subsequently segmented as far into the lung periphery as possible and added to the corresponding lobe by distance measures. E: Lastly, fissures are detected by eigenvalue/eigenvector operations (sagittal view right and left lung). F: Final results of automatic lobe segmentation using bronchi, vessel and fissure information as volume rendering images in posterior view. Lobes are indicated as follows: yellow = right superior lobe, green = middle lobe, orange = right inferior lobe, light blue = left superior lobe, red = lingual, pink = left inferior lobe.

The in-house program YACTA (version 2.7.1.3) segmented the lungs and individual lobes fully automatically on inspiratory images as employed in previous studies [16, 25, 29, 31, 32] and also on the expiratory images, which has not been reported before. YACTA is a non-commercial openly available software for scientific purpose. The process of segmenting individual lung lobes is illustrated in Fig 1. First, airway segmentation was performed with a self-adapting iterative region growing algorithm, followed by the skeletonization of the segmentation result using a sequential topology-preserving 3D thinning algorithm, and the transformation of the skeleton to an acyclic graph representation [20, 33, 34]. The graph is needed for the lobar labeling of the airways with a modified version of the rule based method introduced [35] extended by special rules for anatomical variations. Next, the lungs are segmented with an algorithm based on the method described in [36] which delivers masks of the right and left lung. Then, vessels are segmented using a threshold-based algorithm with an adjusted threshold for each CT. Three-dimensionally (3D) connected vessel objects are generated from the vessel segmentation result. Hereafter, the convex hulls of the bronchi within one lobe are determined and added to the corresponding lobes. Then, vessels are iteratively assigned to the different lobes according to various distance measures and rules. Unassigned vessel objects are divided into smaller vessel objects by an erosion algorithm for the next iteration step. The fissures are enhanced in the CT image by calculating the Hessian matrix and using the eigenvalues, [37] and finally, lobe masks are generated by distance measures to the labeled bronchi and vessels. These lobe masks are eroded by some voxel layers and the final lobe masks are created by a watershed transformation using the eroded lobe masks and the enhanced fissure information as input. The segmentation time per data set was approximately 15 minutes.

### Manual lobe segmentation

**Fig 2:**
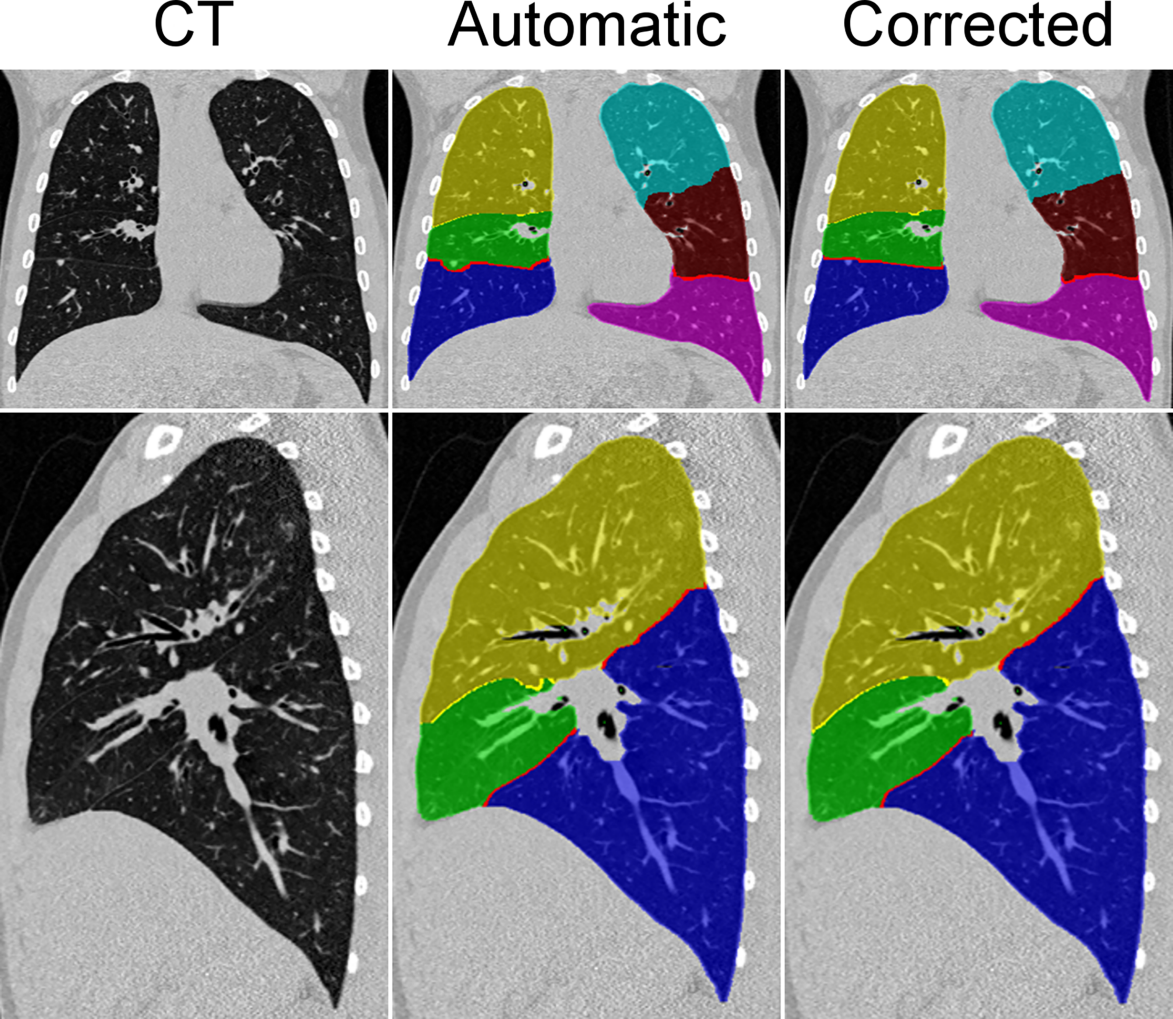
Representative example of the manual correction process of the automatically segmented lung lobes. Coronal as well as sagittal views of the right lung are shown for the original CT data and the results after automatic lobe segmentation (“automatic”) in this CF patient. The course of the fissures was already satisfactorily detected by the software. Manual correction lead to a smoother delineation but did not substantially change the position of the margins of the lobes. Lobes are indicated as follows: yellow = right superior lobe, green = middle lobe, blue = right inferior lobe, light blue = left superior lobe, maroon = lingual, purple magenta = left inferior lobe.

After fully automatic lobe segmentation on inspiratory and expiratory CT datasets, the lobe masks were reviewed by a radiologist, and the masks were corrected manually for each inspiratory and expiratory acquisition to perfectly separate pulmonary lobes (Fig 2). This process took approx. 90 min. of manual interaction per dataset.

### Statistical analysis

All data were recorded in a dedicated database (Excel®, Microsoft Corp., Redmond, USA) and analyses were performed in R 3.4.3 [38]. The data are displayed as mean values and standard deviations. Results of fully-automatic segmentation were compared against manually corrected segmentation results separately for: 1) 4 time points i.e. baseline, 3 month, 12 month and 24 month. 2) individual lobes, i.e. right upper (RUL), middle (RML) and lower (RLL) lobe, as well as left upper lobe (LUL), lingula (LLi) and left lower lobe (LLL) (6 lobes); additionally, results for combined LUL and LLi were generated (LUL+LLi) 3) inspiratory and expiratory scans 4) B30f and B60f kernel. In order to detect spatial discrepancy of the segmentation maps, the Dice and Jaccard indices (***D*** and ***J***) were calculated for aforesaid groups [39]. Let *A* be an automatically segmented lobe and *M* a manually segmented lobe, than the similarity of the two regions can be measured by $D=2\frac{\left|A\cap M\right|}{\left|A|+|M\right|}$ and $J=2\frac{\left|A\cap M\right|}{\left|A\cup M\right|}$, where |*A*| and |*M*| are the number of elements in the regions. For multiple regions the following formulas apply accordingly: $D=2\frac{\sum_{i}\left|A_{i}\cap M_{i}\right|}{\sum_{i}\left|A_{i}|+|M_{i}\right|}$ and $J=2\frac{\sum_{i}\left|A_{i}\cap M_{i}\right|}{\sum_{i}\left|A_{i}\cup M_{i}\right|}$. Both indices range between 0 and 1, from 0 = no overlap to 1 = perfect overlap. In addition, the mean absolute surface distance (average Hausdorff distance) in mm was determined to assess the similarity [40].

Further lobar volume and the air trapping parameter; expiratory to inspiratory ratio of the mean lung attenuation (E/I MLA) [41] were calculated by YACTA for the aforesaid groups. Mean differences (**Δ**) as well as limits of agreement (LoA) in accordance with the approach of Bland and Altman were calculated for fully-automatic vs. manually corrected segmentation [42]. Finally, the Pearson correlation coefficient was calculated for all lobes and time-points. The p-values for time-dependent change in air trapping and lung volume were determined through one way repeated measures ANOVA test using SigmaPlot® (Systat Software GmbH, Erkrath, Germany) software.

## Results

### Spatial overlap of fully-automatic and manually corrected lobe segmentation for 5 and 6 lobes

#### 5 lobes

The average overlap according to the Dice index was 0.98±0.02 for B30f and 0.97±0.02 for B60f on inspiratory scans, respectively. On expiratory scans the results were somewhat worse with 0.86±0.07 for B30f and 0.85±0.08 for B60f kernel, respectively Table 2. In general, the B30f kernel achieved slightly better results on inspiratory and on expiratory scans. The results were stable over all time-points on inspiratory scans. On expiratory B30f scans the overlap improved from 0.83±0.08 to 0.9±0.06 over all time-points, for the B60f scans from 0.81±0.09 to 0.89±0.07 Table 2. The lobe-based analysis revealed that the middle lobe was the most problematic with a mean Dice index dropping from 0.94±0.03 on inspiratory B30f to 0.77±0.22 on expiratory B30f scans S1 Table. The Jaccard index S2 Table as well as the Hausdorff distance S3 Table showed comparable results.

**Table 2:** Summary for Dice index, mean differences in segmented volume and air trapping on B30f scans.

		Baseline		3 months		12 months		24 months		Overall	
		Insp.	Exp.	Insp.	Exp.	Insp.	Exp.	Insp.	Exp.	Insp	Exp.
**RUL**	**Dice Index**	0.98±0.01	0.83±0.13	0.97±0.01	0.88±0.07	0.98±0.01	0.88±0.06	0.98±0.01	0.9±0.07	0.98±0.01	0.87±0.09
	**Δ Volume [cm3]**	-10.63±14.41	8.74±47.35	-7.31±18.43	-27.25±50.32	-7.34±14.77	-25.59±59.02	-10.47±16.5	-22.99±60.4	-8.91±15.82	-16.77±55.29
	**Δ Air trapping**	-0.01±0.01		0±0.02		-0.01±0.01		-0.01±0.01		0±0.01	
**RML**	**Dice Index**	0.94±0.02	0.78±0.19	0.93±0.02	0.77±0.23	0.94±0.02	0.7±0.3	0.93±0.04	0.83±0.12	0.94±0.03	0.77±0.22
	**Δ Volume [cm3]**	-6.91±12.76	-12.01±41.1	-6.66±16.78	-9.3±48	-7.65±16.05	26.73±69.6	-8.34±20.46	-7.1±37.82	-7.4±16.4	-0.42±51.94
	**Δ Air trapping**	0±0.02		0.01±0.03		-0.02±0.06		0±0.02		0±0.04	
**RLL**	**Dice Index**	0.98±0.01	0.87±0.07	0.98±0.01	0.86±0.13	0.98±0.01	0.88±0.07	0.98±0.01	0.91±0.07	0.98±0.01	0.88±0.09
	**Δ Volume [cm3]**	18.85±13.4	29.77±41.82	14.32±12.59	54.53±44.44	15.99±14.09	13.56±60.63	20.59±19.24	45.13±67.2	17.41±14.92	35.75±55.53
	**Δ Air trapping**	-0.04±0.03		-0.02±0.02		-0.02±0.02		-0.01±0.02		-0.02±0.03	
**LUL**	**Dice Index**	0.96±0.01	0.73±0.19	0.94±0.06	0.81±0.11	0.95±0.02	0.84±0.1	0.96±0.02	0.84±0.1	0.96±0.03	0.8±0.14
	**Δ Volume [cm3]**	10.32±14.95	-30.88±103.34	6.19±65.3	-17.26±67.65	7.06±42.42	-2.13±44.88	19.34±40.12	-15.01±57.54	10.74±43.98	-16.32±70.75
	**Δ Air trapping**	-0.04±0.04		-0.01±0.02		-0.01±0.02		-0.01±0.02		-0.02±0.03	
**LLi**	**Dice Index**	0.9±0.04	0.37±0.29	0.82±0.23	0.62±0.28	0.88±0.04	0.63±0.31	0.88±0.06	0.73±0.22	0.87±0.12	0.59±0.3
	**Δ Volume [cm3]**	-23.1±63.98	36.03±62.92	-16.86±45.96	17.3±47.28	-10.61±44.68	14.71±53.59	-7.3±51.87	2.27±43.33	-14.33±51.05	17.62±52.46
	**Δ Air trapping**	-0.01±0.11		-0.02±0.07		0±0.04		-0.01±0.02		-0.01±0.07	
**LLL**	**Dice Index**	0.98±0.01	0.8±0.11	0.97±0.06	0.83±0.11	0.98±0.01	0.87±0.09	0.98±0.01	0.89±0.09	0.98±0.03	0.85±0.11
	**Δ Volume [cm3]**	13.86±66.59	32.75±76	11.96±74.45	27.65±44.65	6.05±22.13	2.14±44.25	-8.58±27.93	28.18±39.68	5.7±52.23	22.68±53.27
	**Δ Air trapping**	-0.05±0.03		-0.04±0.03		-0.03±0.02		-0.02±0.03		-0.03±0.03	
**LUL+LLi**	**Dice Index**	0.98±0.01	0.81±0.08	0.97±0.05	0.87±0.07	0.98±0.01	0.88±0.08	0.98±0.01	0.91±0.06	0.98±0.03	0.87±0.08
	**Δ Volume [cm3]**	-12.78±65.42	5.15±68.16	-10.67±74.94	0.05±36.81	-3.55±23.59	16.51±43.88	12.05±28.32	-12.74±36.18	-3.59±52.44	2.24±48.07
	**Δ Air trapping**	-0.01±0.02		-0.01±0.02		-0.01±0.01		-0.01±0.01		-0.01±0.02	

Overlap calculated by the Dice index (%), mean difference (**Δ)** for lobar volumes [cm^3^], and mean differences for lobar air trapping comparing fully automatic and manually corrected segmentation are given for baseline, 3, 12 and 24 months. The last column summarizes all time points. All values are separately calculated for the right upper (RUL), middle (RML) and lower lobe (RLL), the left upper lobe (LUL), the lingula (LLi), the left lower lobe (LLL), and also combining left upper lobe and lingula into one lobe (LUL+LLi).

#### 6 lobes

Considering the lingula as an independent lobe may hamper automatic but also manual segmentation due to missing fissural borders. Surprisingly, on inspiratory scans the relative difference regarding the Dice index was only 1.02% and 1.03% for B30f and B60f, respectively. This observation was more pronounced on the expiratory scans with a lower mean Dice index of about 3.49% and 4.71%, respectively S4 Table. As expected, the overlap for the lingula was the lowest with a mean overlap of 0.87±0.12 on inspiratory and 0.59±0.3 on expiratory B30f scans. On inspiratory scans, the results were nearly stable over all time-points whereas all three indices show a concordant improvement for overlap on expiratory scans from baseline to 24 months. The most distinct improvement occurred for the lingula with the Dice index increasing from 0.37±0.29 to 0.73±0.22 on expiratory B30f scans S1 Table.

### Lobe-based volumes and air trapping for fully automatic and manually corrected lobe segmentation

The mean differences in lobar volumes calculated in accordance with the approach of Bland and Altman were generally low, ranging from 5.7±52.23 cm^3^ for the right upper lobe, to 17.41±14.92 cm^3^ for the right lower lobe on inspiratory CT B30f. On expiratory CT, higher differences were noted in some lobes, for example 35.75±55.53 cm^3^ for the right lower lobe and 22.68±53.27 cm^3^ for the left lower lobe using the B30f kernel Table 2 and S2 Table. As expected, the results for B60f were slightly worse S6 and S8 Tables. The Pearson correlation coefficient was ranging from 0.98 to 1 in inspiratory B30f scans and from 0.52 to 0.92 in expiratory B30f scans Table 3.

**Table 3:** Temporal development of lung volume and air trapping over 24 months.

Lung		Baseline	3 month	12 month	24 month	p
**Volume [cm**^**3**^**]**						
**B30f Insp.**	**manually**	3653.26 ±909.92	3643.03 ±1014.31	3973.5 ±1184.7	4440.41 ±1238.32	<0.0001
	**automatic**	3650.87 ±908.89	3641.38 ±1011.11	3969.99 ±1181.85	4435.17 ±1236.43	<0.0001
**B60f Insp.**	**manually**	3608.79 ±896.88	3643.03 ±1014.31	3972.8 ±1184.55	4505.61 ±1253.03	<0.0001
	**automatic**	3597.88 ±892.34	3641.38 ±1011.11	3968.75 ±1180.54	4495.61 ±1249.2	<0.0001
**B30f Exp.**	**manually**	1259.34 ±374.04	1310.39 ±512.75	1430.32 ±476.48	1615.05 ±509.09	<0.0003
	**automatic**	1194.94 ±396.65	1264.71 ±540.25	1396.96 ±487.09	1584.57 ±533.53	<0.0002
**B60f Exp.**	**manually**	1265.22 ±376.85	1310.39 ±512.75	1429.99 ±493.21	1615.05 ±509.09	<0.0004
	**automatic**	1228.59 ±376.63	1296.5 ±522.06	1386.8 ±478.32	1598.22 ±525.09	<0.0003
**Air trapping (E/I MLA)**						
**B30f**	**manually**	0.63±0.1	0.64±0.1	0.65±0.09	0.67±0.08	0.59
	**automatic**	0.65±0.09	0.66±0.09	0.66±0.08	0.68±0.07	0.74
**B60f**	**manually**	0.62±0.1	0.63±0.1	0.64±0.09	0.66±0.08	0.59
	**automatic**	0.64±0.09	0.64±0.09	0.66±0.09	0.67±0.08	0.50

Mean and standard deviation for total lung volume [cm^3^] and air trapping, calculated for fully automatic and manually corrected segmentations at baseline 3, 12 and 24 months. Total lung volume and air trapping increased steadily over time. Changes were significant for volume changes but not significant for air trapping.

In comparison to the lobar volume, the mean differences in air trapping comparing fully automatic with manually corrected segmentation were much lower, ranging from 0±0.01 for the right upper lobe to 0.03±0.03 for the left lower lobe in B30f scans Table 2 and S9 Table. The results for B60f scans were comparable showing a slightly higher Pearson correlation coefficient for the lingula with 0.82 and a slightly lower Pearson correlation coefficient for the RLL and LUL with 0.95 and 0.96 respectively S10 Table. Overall, the Pearson correlation coefficients calculated for lobe-based air trapping were higher than for lobar volume Table 3.

## Discussion

In order to introduce QCT into routine patient work-up as a quantitative endpoint, it is necessary to agree on and strictly control for examination protocols, post-processing, measurement parameters and parameter interpretation [43]. Specifically, lung segmentation as an important step of densitometry for emphysema but also for air trapping is variable between different software for the whole lung and for lung lobes [25, 44]. To the best of our knowledge, reports on lung lobe segmentation on expiratory CT are missing, which however is a pre-requisite for quantification of air trapping on a lobar level. A regional correlative assessment of airway and parenchymal disease is highly desirable in the process of understanding regional evolution of airway diseases such as CF and also COPD. It is known that CF manifestations in the lung are heterogeneous between patients, but also within the same patient at a given time-point as well as over time. This emphasizes the need for a quantitative assessment of regional disease activity, which is not possible with lung function testing (spirometry, lung clearance index).

The present study sought to validate a state-of-the-art lung lobe segmentation algorithm for inspiratory and expiratory CT as a novel method for the assessment of regional disease in a highly challenging population, school-age children with CF. The obstacles to automatic lobe segmentation are manifold: 1.) Smaller lungs, vessels and airways but similar resolution of CT compared to adults. 2.) Displacement of anatomical landmarks in deep end-expiration due to inhomogeneously distributed air trapping. 3) Higher lung density and thus lower differences between lung, chest wall, and airway wall etc. due to the young age as well as the examination technique. The deeper expiration to spirometer-controlled FRC-levels leads to a higher sensitivity to air trapping but increases the segmentation error in return [45]. The influence of lung density is partially reduced with ageing since lung density on CT decreases linearly in the first few years of life, and thereafter approximates adult levels during adolescence [46], but continues to decrease slowly at older ages [47]. Therefore, with decreasing lung density it can be assumed that our segmentation algorithm will deliver even better results in adults. However, an assessment of lung disease in earlier lung disease is valuable in order to start treatment early and to prolong the occurrence of irreversible lung damage. Thus, we focused on the group of children in school-age from 8 to 14 years.

The results of the present study show, that the overlap between fully-automatically generated and manually corrected lobe segmentation masks is very high for both upper and lower lobes, since these lobes are clearly limited by the major fissures. The overlap for middle lobe as well as lingula is not yet completely satisfactory with a Dice index of 0.77±0.22 and 0.59±0.3 on expiratory CT using a B30f kernel, respectively Table 2. Due to the lower noise level, the use of a soft B30f algorithm was slightly advantageous compared to a B60f algorithm. Reasons for this are the better segmentation results for the airways and vessels as well as clearer depiction and little deformation of the fissures in inspiration. This holds true especially for baseline exams in which the lung density in expiration was very high compared to anatomical landmarks (vessels, fissures, chest wall). With progressive maturation (baseline to 24 month) of the lung structures the results for the Dice index improved from 0.83±0.08 to approximately 0.9±0.06 for expiratory scans S4 Table. Though the actual overlap for baseline exams for expiratory scans was somewhat lower, the segmentation of 6 instead of 5 lobes resulted only in a slight worsening in the overall overlap by approximately -1.02% for inspiratory scans and -3.49% for expiratory scans S4 Table. The mean differences in lobar volumes calculated in accordance with the approach of Bland and Altman comparing fully automatic with manually corrected segmentation were generally low, ranging from only 5.7±52.23 cm^3^ for the right upper lobe to 17.41±14.92 cm^3^ for the right lower lobe on inspiratory CT S5 and S6 Tables, and showing only slightly higher differences on expiratory CT scans for some lobes S7 and S8 Tables. Lung volume showed an increase over time, most likely due to growth Table 3. In comparison to the lobar volume the mean differences in lobar air trapping as the final variable to be assessed with the new segmentation approach suggested in this manuscript were negligible S9 and S10 Tables. Furthermore, the Pearson correlation coefficients for lobe-based air trapping were clearly higher compared to the Pearson correlation coefficients calculated for lobar volumes on expiratory scans Table 2. As mentioned above, the overlap with regard to the middle lobe and lingula is still not completely satisfactory, but we could show that the actual error rate regarding air trapping quantification was much lower than the error induced by mis-segmentation by the automatic algorithm, because very likely not all of the mis-segmented lung will be affected by air trapping thus assigned to the wrong lung lobe. The increase in air trapping is most likely based on progressing small airways disease Table 3.

In light of these results, the evaluation of 6 lobes seems feasible in order to allow for a finer graduation of the analysis of structural and functional lung damage. This approach will match previous work using visual scoring, in which also the lingual is usually treated as a lung lobe [48, 49]. Overall, we believe that the achieved error rate for air trapping is acceptable, and that in the future an automatic evaluation of air trapping of CF lung disease using B30f kernels is therefore possible with the suggested approach.

An alternative approach employing visual scoring for air trapping with expiratory CT scans is subject to very high inter-reader variability, and does not allow for a sensitive grading of the severity of trapped air. The AREST CF collaborative reported a kappa value for air trapping of 0.55 for intra-reader agreement in a group of 96 children diagnosed by newborn screening (mean age 1.1 years, range 0.3–3.3 years) [11]. The CT protocol of the above mentioned study did not include entirely volumetric scans but few slices for sampling the lung parenchyma. Though slice sampling has been reported to be representative in some studies [17], some studies reported that it might underestimate air trapping [50], and is probably not useful in the setting of longitudinal studies. Better results were reported by AREST CF in slightly older patients using volumetric scans (mean age 3.4, range 1–6 years), with kappa of 0.8 for air trapping reflecting better intra-reader agreement [7]. A software-supported scoring system was able to improve the intra- and inter-reader intra-class correlation coefficients to >0.9 for air trapping, but still requires human readers and currently does not extract lobe-based information [17]. Furthermore a poor kappa for air trapping of 0.17–0.23 for intra-reader agreement in a group of 294 subjects, including normal non-smokers, smokers without COPD, and smokers with GOLD Stage I-IV COPD was reported [51]. In view of these results, and the agreement for air trapping between fully automatic and manually corrected lobe segmentation across the population in our study is, much better than previously reported agreement for air trapping between human readers. Also, results of airway quantification will not be affected at all, as airway segmentation is the pre-requisite for lobe segmentation as explained above, and missing spatial overlap is a concern of the periphery of the lobe where no evaluable airways are present.

## Conclusions

In conclusion, fully-automatic lobe segmentation delivers excellent results for inspiratory and, with some limitations with regard to the middle lobe as well as the lingula, satisfactory results for expiratory CT scans. Currently, the process of manual correction of CT data amounted to approx. 90 min per patient. For the total population, the above mentioned overall high agreement for fully automatic with manually corrected lung lobe segmentation seems highly acceptable, especially in view of the results achieved by visual scoring in the past. However, the relative wide range of the limits of agreement means that in the individual patient segmentation failures may occur and warrant at least visual inspection of the automatically generated segmentation results before use of the results in clinical routine. We feel that it is feasible to accept fully-automatic segmentation in the process of air trapping quantification, in order to minimize user-interaction and allow faster throughput of large imaging datasets. It will thus likely become an important component for lobe-based quantification of airways diseases such as CF. Newer methods are currently under development that further improve expiratory lobar segmentation by the introduction of a registration step, which need to be validated in further studies. Furthermore we believe that automated regional quantification of lung disease in CF will assist in developing reliable imaging-derived biomarkers for disease severity, which provide a more accurate grading of disease burden than spirometry, and will help to find personalized therapy. The methods stated here will subsequently be employed to study the full dataset of the total study population with regard to longitudinal air trapping, parenchymal and airway changes.

## Supporting information

S1 Fig Regression lines for lobar lung volumes on inspiration B30f scans. (PDF)

S2 Fig Bland-Altman plots for lobar lung volumes on inspiration B60f scans. (PDF)

S3 Fig Regression lines for lobar lung volumes on inspiration B30f scans. (PDF)

S4 Fig Bland-Altman plots for lobar lung volumes on inspiration B60f scans. (PDF)

S5 Fig Regression lines for lobar lung volumes on expiration B30f scans. (PDF)

S6 Fig Bland-Altman plots for lobar lung volumes on expiration B60f scans. (PDF)

S7 Fig Regression lines for lobar lung volumes on expiration B30f scans. (PDF)

S8 Fig Bland-Altman plots for lobar lung volumes on expiration B60f scans. (PDF)

S9 Fig Regression lines for air-trapping on B30f scans. (PDF)

S10 Fig Bland-Altman plots for air-trapping on B60f scans. (PDF)

S11 Fig Regression lines for air-trapping on B30f scans. (PDF)

S12 Fig Bland-Altman plots for air-trapping on B60f scans. (PDF)

S1 Table Dice index of overlap between fully automatic and manually corrected segmentation of lung lobes. Overlap calculated by the Dice index for fully automatic and manually corrected segmentation are given for inspiration (Insp) and expiration (Exp) and each for B30f and B60f kernel at baseline, 3, 12 and 24 months. The last column summarizes all time points. Mean and standard deviations (mean±sd) are separately calculated for the right upper (RUL), middle (RML) and lower lobe (RLL), the left upper lobe (LUL), the lingula (LLi), the left lower lobe (LLL), and also combining left upper lobe and lingula into one lobe (LUL+LLi).
(PDF)

S2 Table Jaccard index of overlap between fully automatic and manually corrected segmentation of lung. Overlap calculated by the Jaccard index for fully automatic and manually corrected segmentation are given for inspiration (Insp) and expiration (Exp) and each for B30f and B60f kernel at baseline, 3, 12 and 24 months. The last column summarizes all time points. Mean and standard deviations (mean±sd) are separately calculated for the right upper (RUL), middle (RML) and lower lobe (RLL), the left upper lobe (LUL), the lingula (LLi), the left lower lobe (LLL), and also combining left upper lobe and lingula into one lobe (LUL+LLi).
(PDF)

S3 Table Hausdorff distance of overlap between fully automatic and manually corrected segmentation of lung. Overlap calculated by the Hausdorff distance for fully automatic and manually corrected segmentation are given for inspiration (Insp) and expiration (Exp) and each for B30f and B60f kernel at baseline, 3, 12 and 24 months. The last column summarizes all time points. Mean and standard deviations (mean±sd) are separately calculated for the right upper (RUL), middle (RML) and lower lobe (RLL), the left upper lobe (LUL), the lingula (LLi), the left lower lobe (LLL), and also combining left upper lobe and lingula into one lobe (LUL+LLi).
(PDF)

S4 Table Overlap comparison for 5 vs. 6 lobes. The indices of overlap summarized for 5 and 6 lobes at baseline, 3, 12, 24 month. Mean and standard deviation (mean±sd) are given for inspiration (Insp) and expiration (Exp) and each for B30f and B60f kernel. When treating LUL and LLi as a combined lobe (LUL+LLi, 5 lobes), the results were somewhat better than for separate analysis (LUL and LLi; 6 lobes), which is mainly due to the missing fissure, i.e. unequivocal separation, between both lobes. The relative difference between the two approaches are calculated as relative difference in [%].
(PDF)

S5 Table Lobe volume determination on manual and automatic segmentation maps (inspiration B30f scans). Lobar volumes [cm^3^] calculated for fully automatic and manually corrected segmentation on inspiration B30f scans at baseline, 3, 12 and 24 months. The last column summarizes all time points. All values are separately calculated for the right upper (RUL), middle (RML) and lower lobe (RLL), the left upper lobe (LUL), the lingula (LLi), the left lower lobe (LLL), and also combining left upper lobe and lingula into one lobe (LUL+LLi). Both methods are compared in accordance with the approach of Bland-Altman giving mean differences (Δ), limits of agreement (LoA) and two regression coefficients (Intercept / Slope and Pearson’s correlation coefficient).
(PDF)

S6 Table Lobe volume determination on manual and automatic segmentation maps (inspiration B60f scans). Lobar volumes [cm^3^] calculated for fully automatic and manually corrected segmentation on inspiration B60f scans at baseline, 3, 12 and 24 months. The last column summarizes all time points. All values are separately calculated for the right upper (RUL), middle (RML) and lower lobe (RLL), the left upper lobe (LUL), the lingula (LLi), the left lower lobe (LLL), and also combining left upper lobe and lingula into one lobe (LUL+LLi). Both methods are compared in accordance with the approach of Bland-Altman giving mean differences (Δ), limits of agreement (LoA) and two regression coefficients (Intercept / Slope and Pearson’s correlation coefficient).
(PDF)

S7 Table Lobe volume determination on manual and automatic segmentation maps (expiration B30f scans). Lobar volumes [cm^3^] calculated for fully automatic and manually corrected segmentation on expiration B30f scans at baseline, 3, 12 and 24 months. The last column summarizes all time points. All values are separately calculated for the right upper (RUL), middle (RML) and lower lobe (RLL), the left upper lobe (LUL), the lingula (LLi), the left lower lobe (LLL), and also combining left upper lobe and lingula into one lobe (LUL+LLi). Both methods are compared in accordance with the approach of Bland-Altman giving mean differences (Δ), limits of agreement (LoA) and two regression coefficients (Intercept / Slope and Pearson’s correlation coefficient).
(PDF)

S8 Table Lobe volume determination on manual and automatic segmentation maps (expiration B60f scans). Lobar volumes [cm^3^] calculated for fully automatic and manually corrected segmentation on expiration B60f scans at baseline, 3, 12 and 24 months. The last column summarizes all time points. All values are separately calculated for the right upper (RUL), middle (RML) and lower lobe (RLL), the left upper lobe (LUL), the lingula (LLi), the left lower lobe (LLL), and also combining left upper lobe and lingula into one lobe (LUL+LLi). Both methods are compared in accordance with the approach of Bland-Altman giving mean differences (Δ), limits of agreement (LoA) and two regression coefficients (Intercept / Slope and Pearson’s correlation coefficient).
(PDF)

S9 Table E/I MLA determination on manual and automatic segmentation maps (B30f scans). Air trapping (E/I MLA) calculated for fully automatic and manually corrected segmentation on B30f scans at baseline, 3, 12 and 24 months. The last column summarizes all time points. All values are separately calculated for the right upper (RUL), middle (RML) and lower lobe (RLL), the left upper lobe (LUL), the lingula (LLi), the left lower lobe (LLL), and also combining left upper lobe and lingula into one lobe (LUL+LLi). Both methods are compared in accordance with the approach of Bland-Altman giving mean differences (Δ), limits of agreement (LoA) and two regression coefficients (Intercept / Slope and Pearson’s correlation coefficient).
(PDF)

S10 Table E/I MLA determination on manual and automatic segmentation maps (B60f scans). Air trapping (E/I MLA) calculated for fully automatic and manually corrected segmentation on B60f scans at baseline, 3, 12 and 24 months. The last column summarizes all time points. All values are separately calculated for the right upper (RUL), middle (RML) and lower lobe (RLL), the left upper lobe (LUL), the lingula (LLi), the left lower lobe (LLL), and also combining left upper lobe and lingula into one lobe (LUL+LLi). Both methods are compared in accordance with the approach of Bland-Altman giving mean differences (Δ), limits of agreement (LoA) and two regression coefficients (Intercept / Slope and Pearson’s correlation coefficient).
(PDF)
