# Supplementary figures and images for: Validation of automated lobe segmentation on paired inspiratory-expiratory chest CT in 8-14 year-old children with cystic fibrosis
Source: PLoS One. 2018 Apr 9;13(4):e0194557. doi: 10.1371/journal.pone.0194557 (PMC5890971; doi:10.1371/journal.pone.0194557)

**RUL B30f**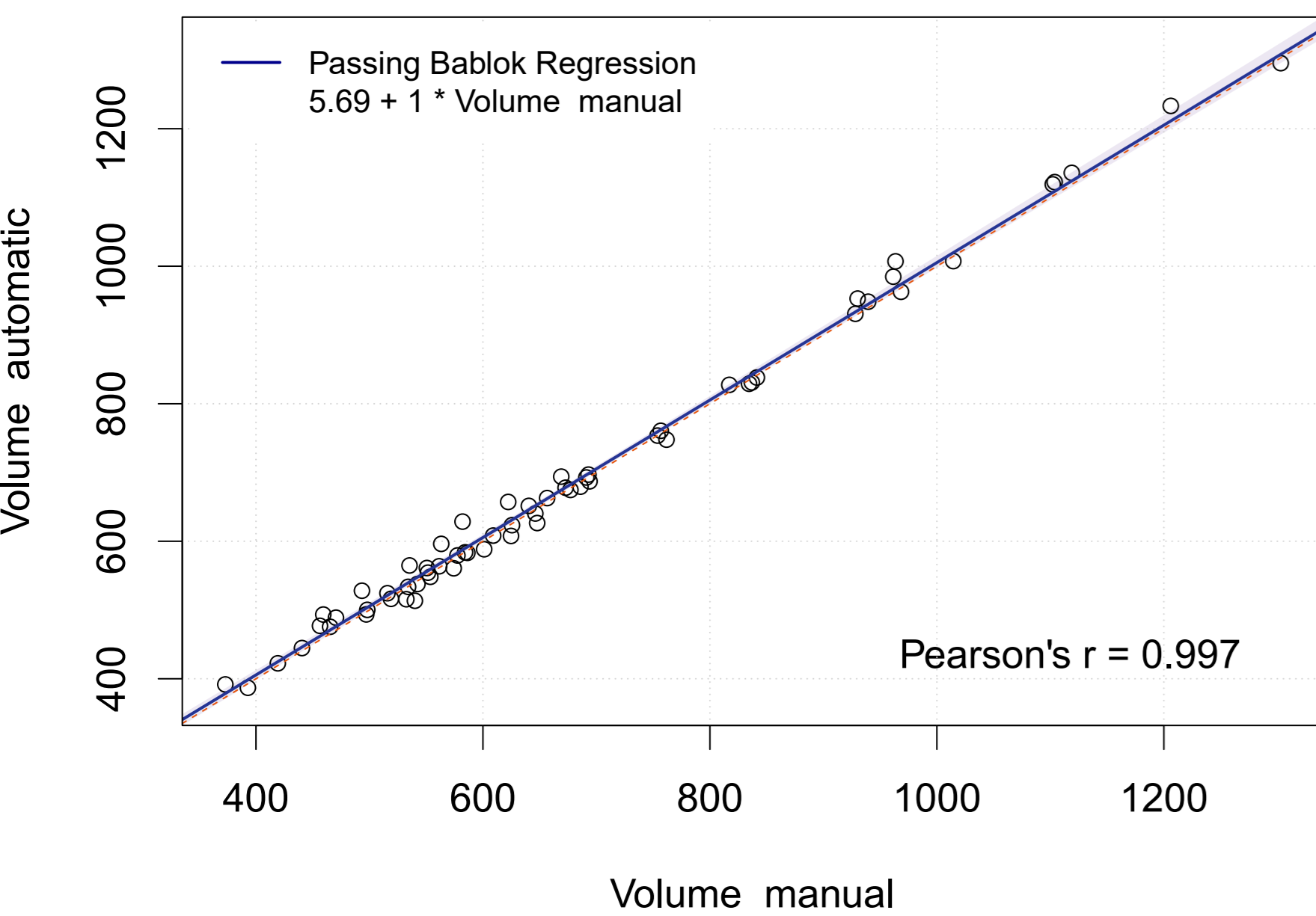**RML B30f**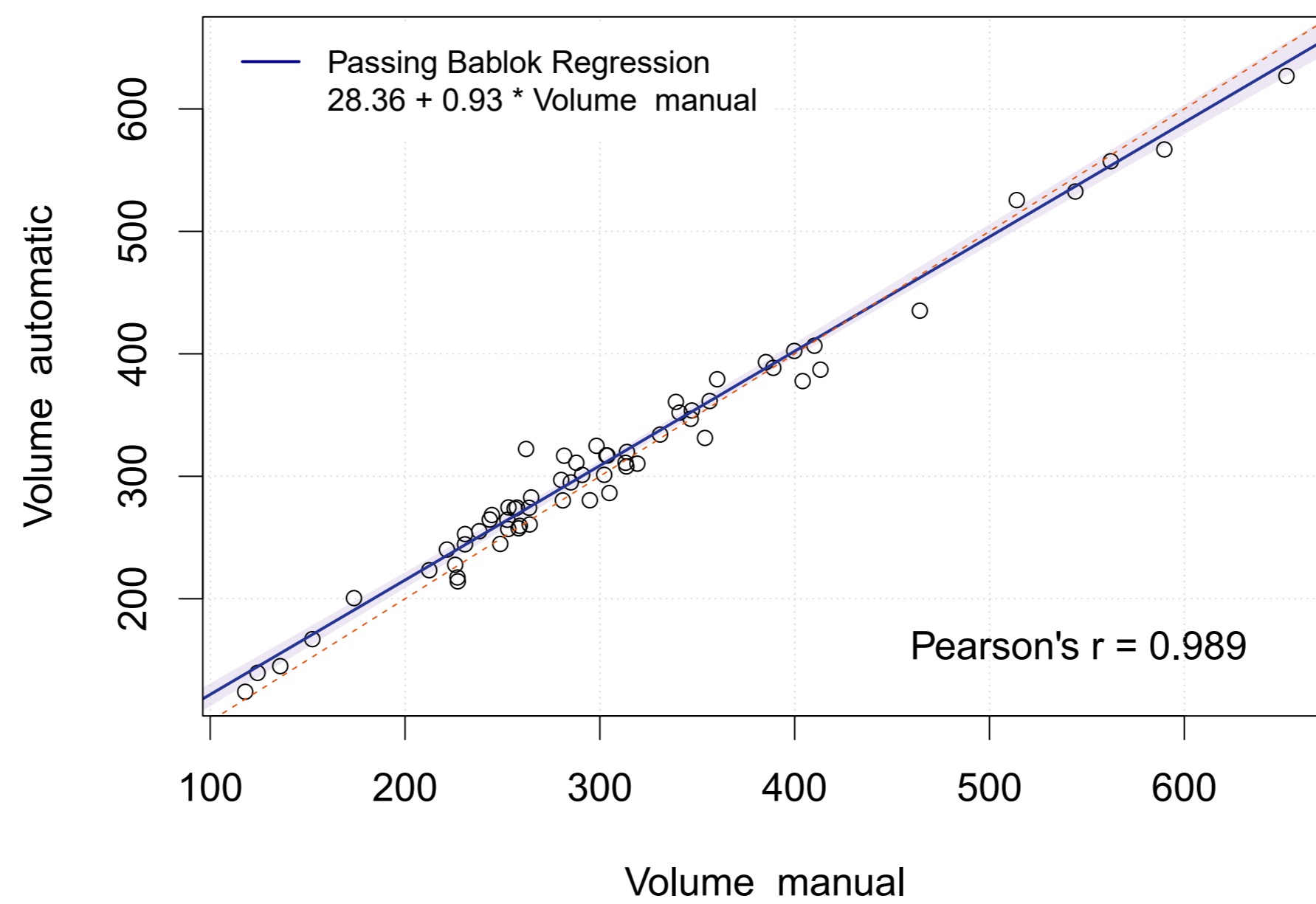**RLL B30f**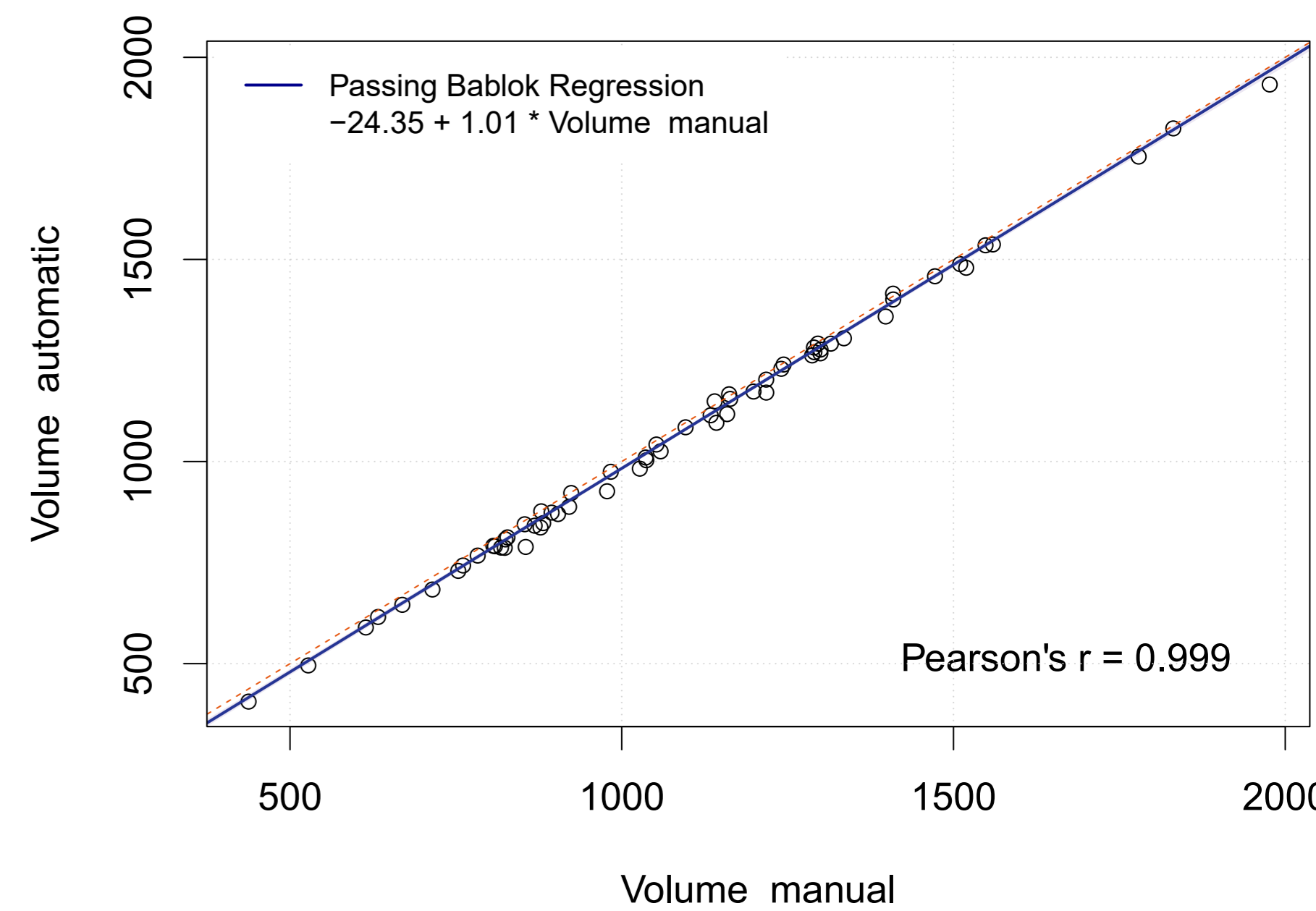**LUL B30f**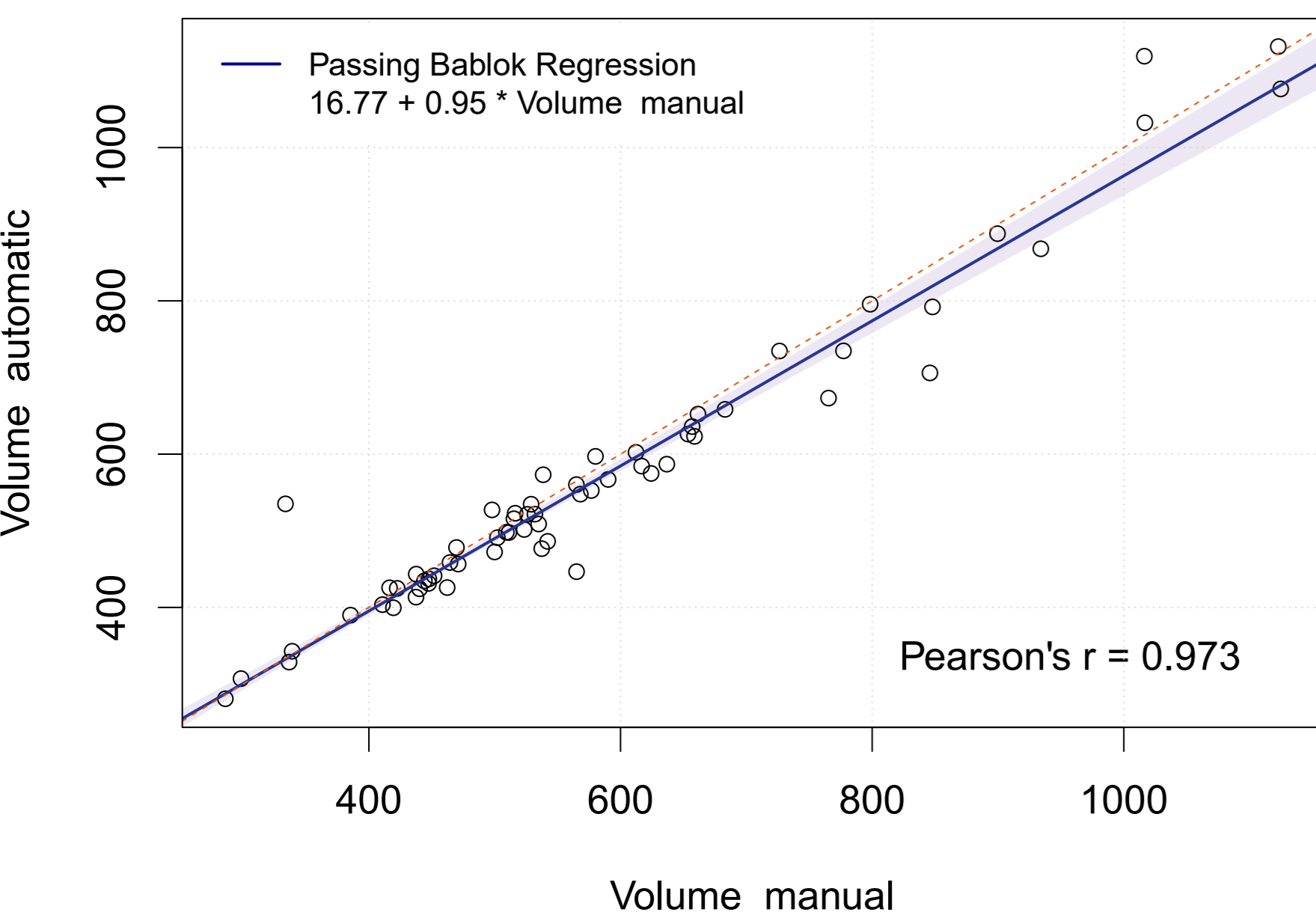**LLi B30f**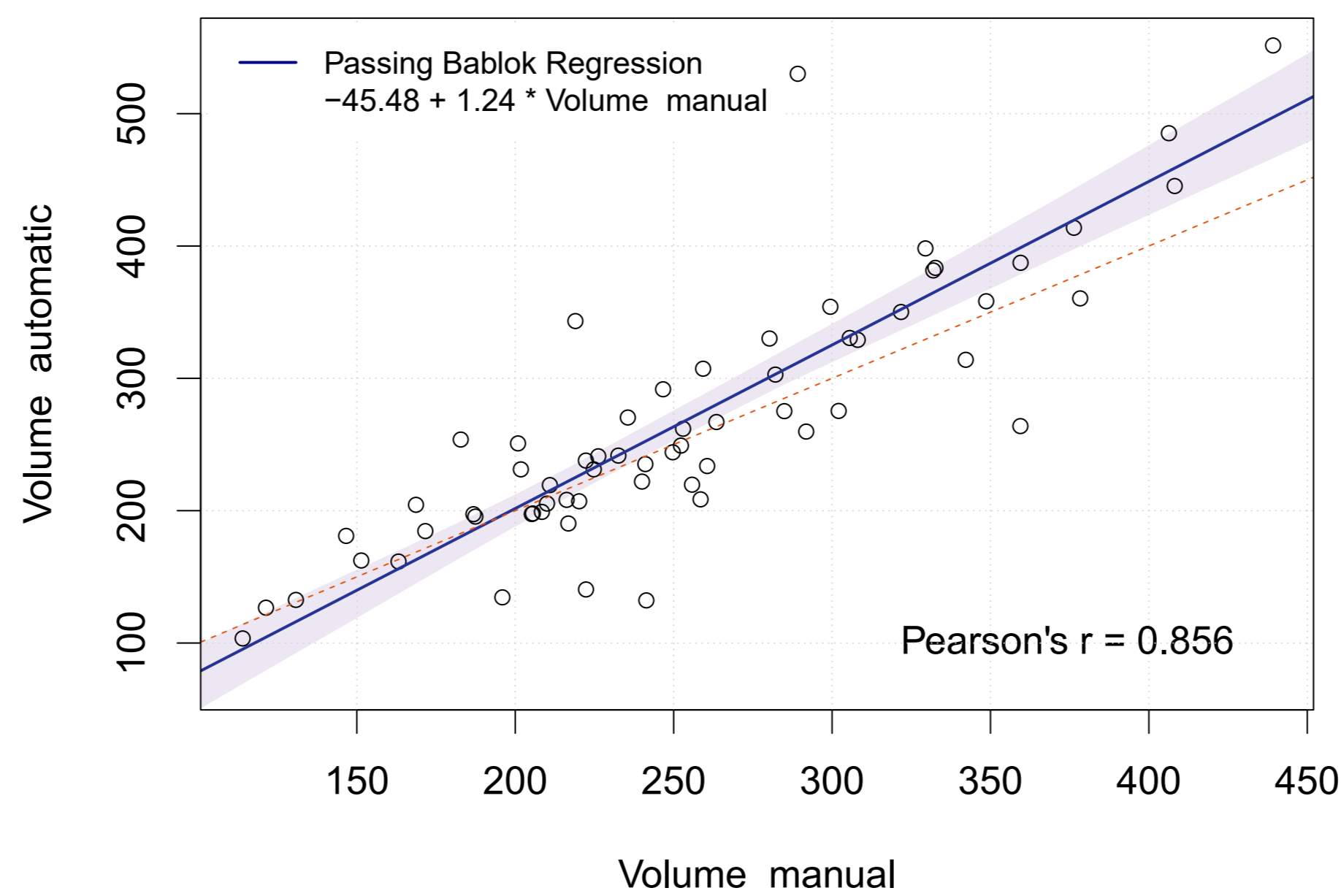**LLL B30f**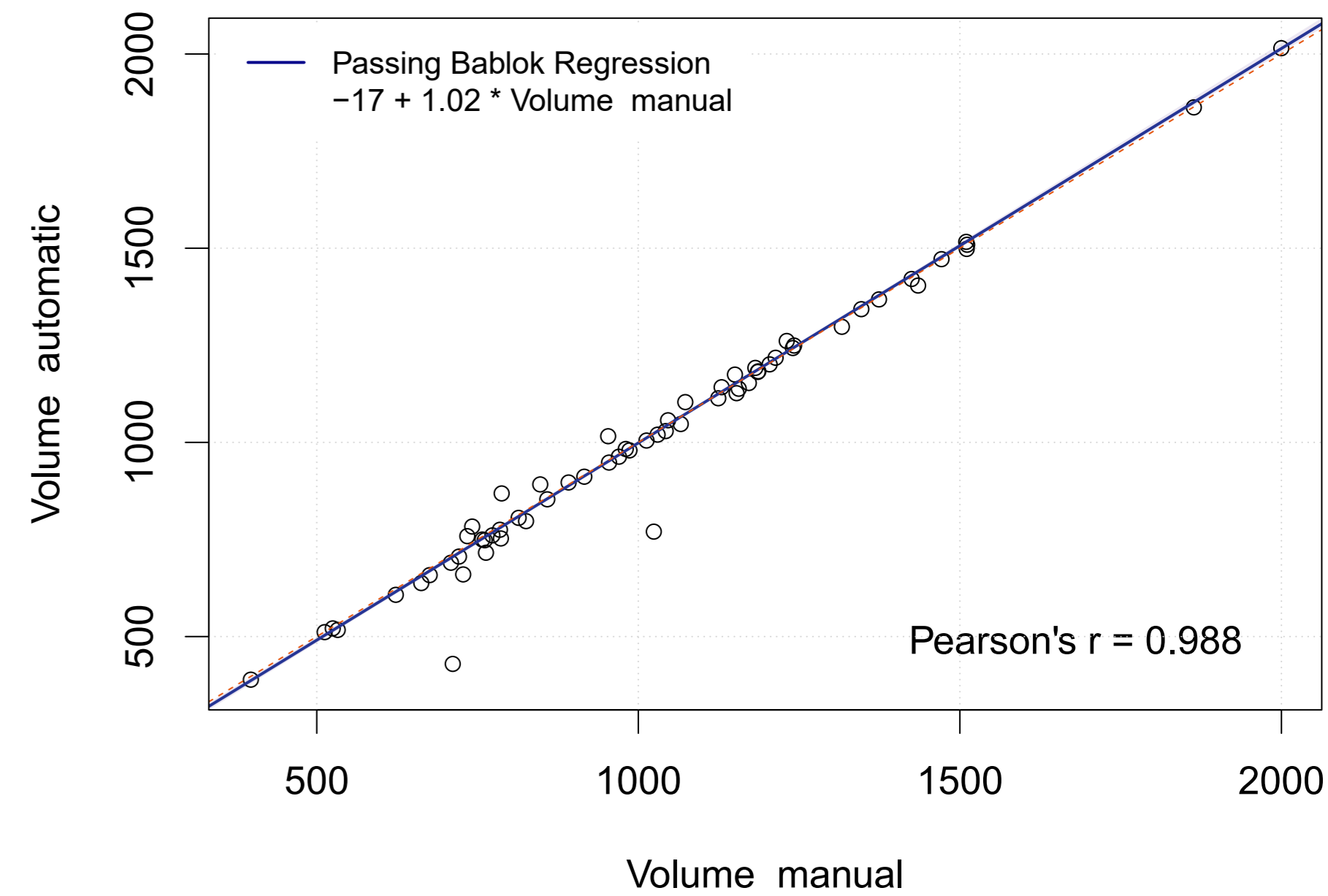**LUL+LLi B30f**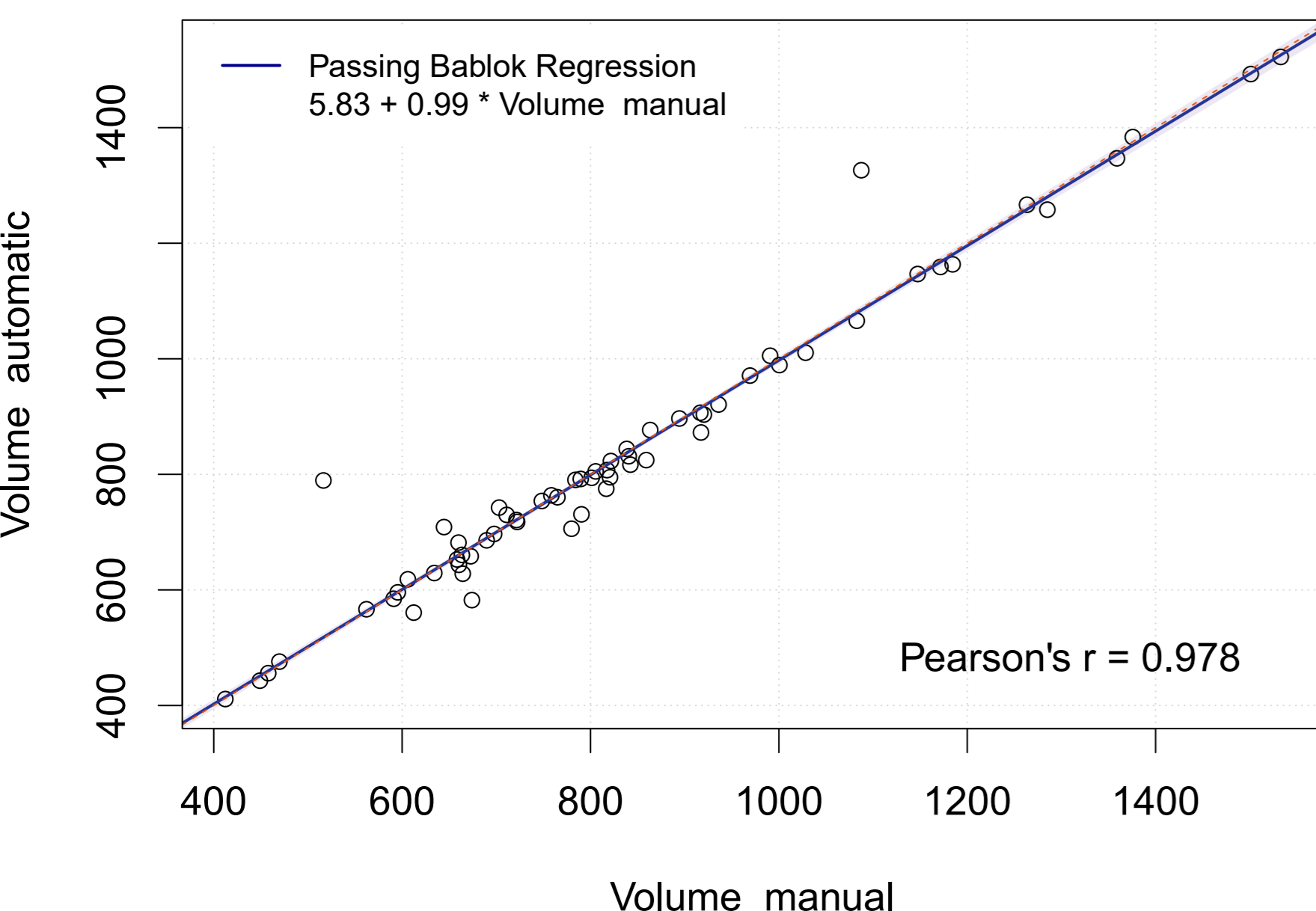

Supplement: S1 Fig — (PDF) [file pone.0194557.s001.pdf]

**RUL B30f**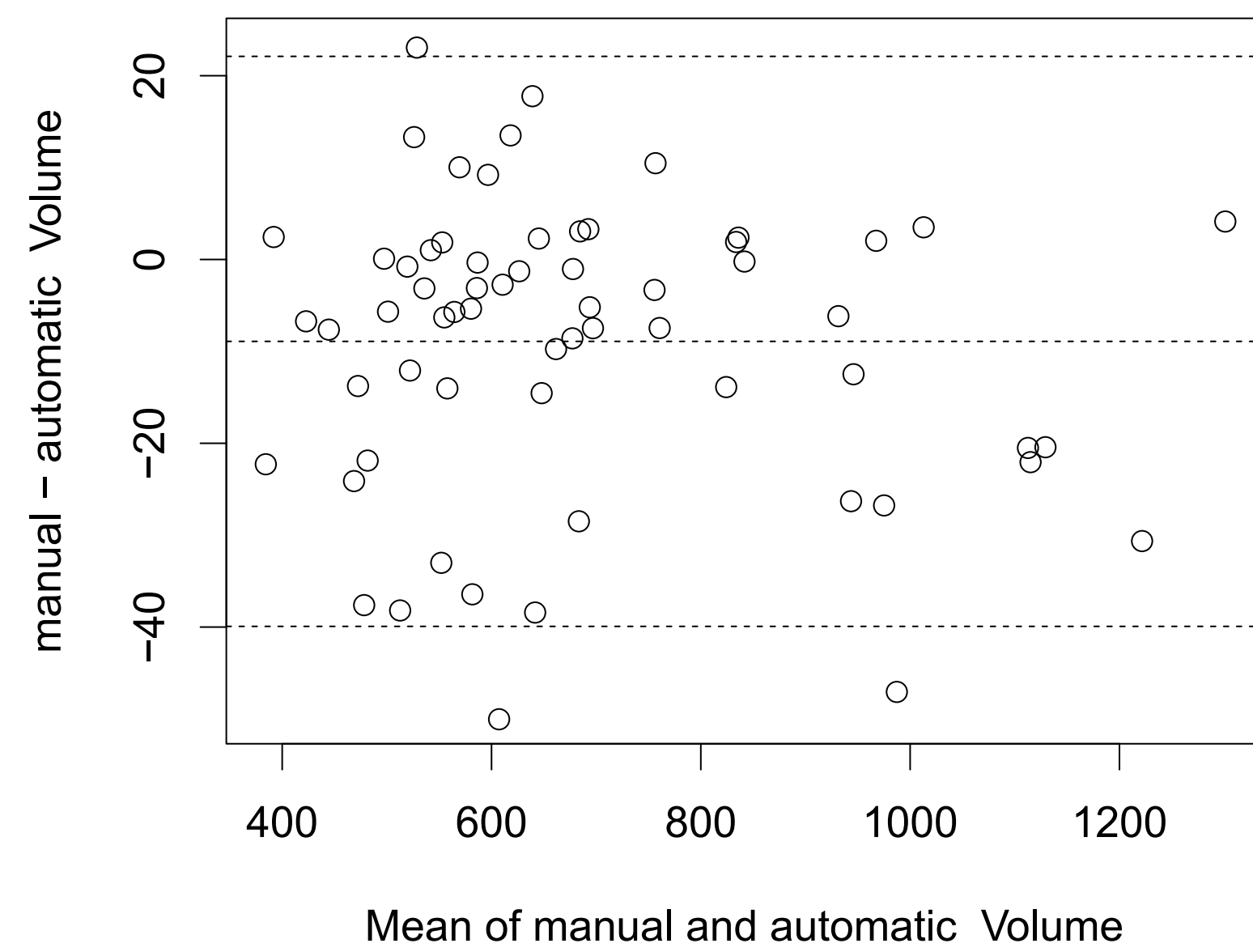**RML B30f**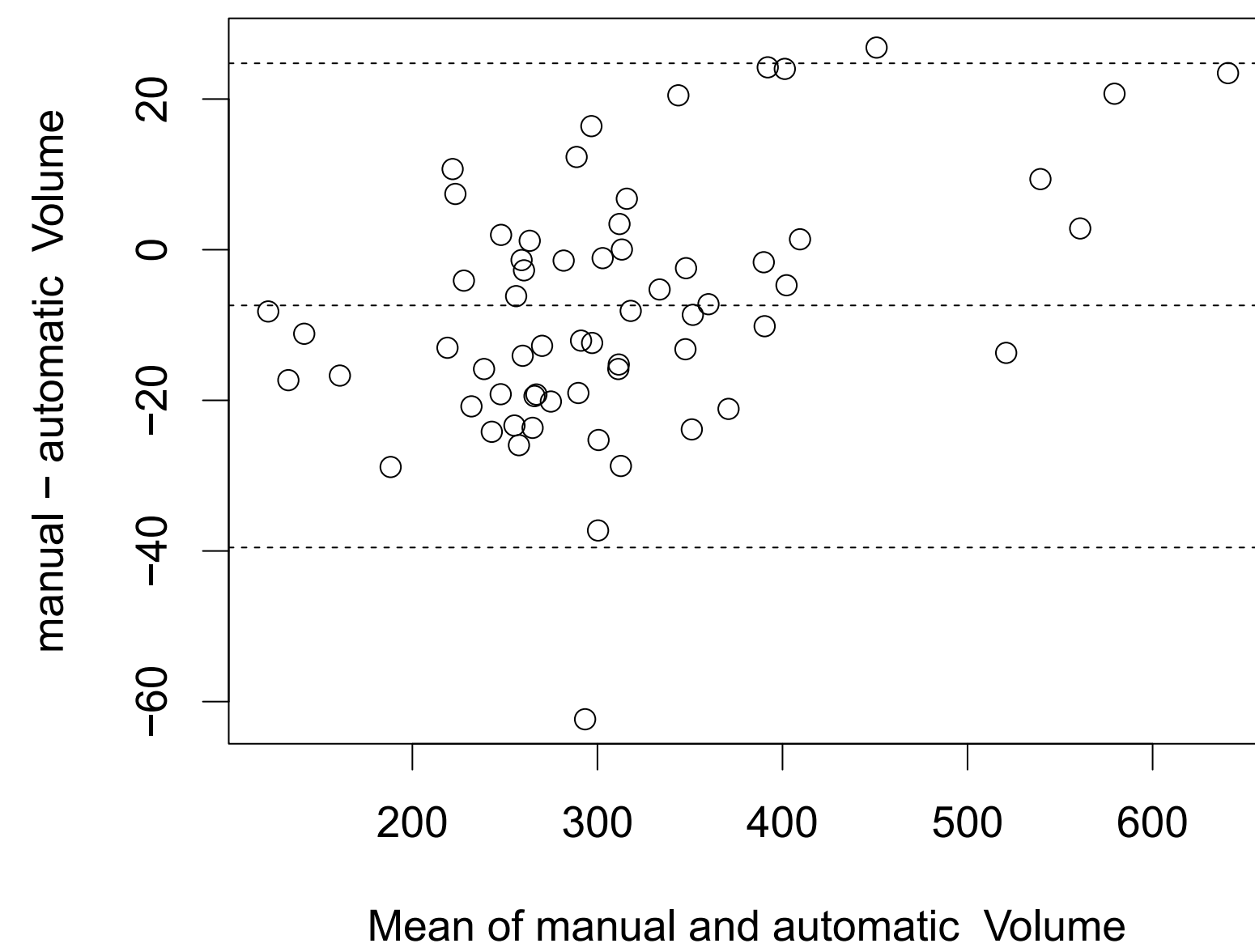**RLL B30f**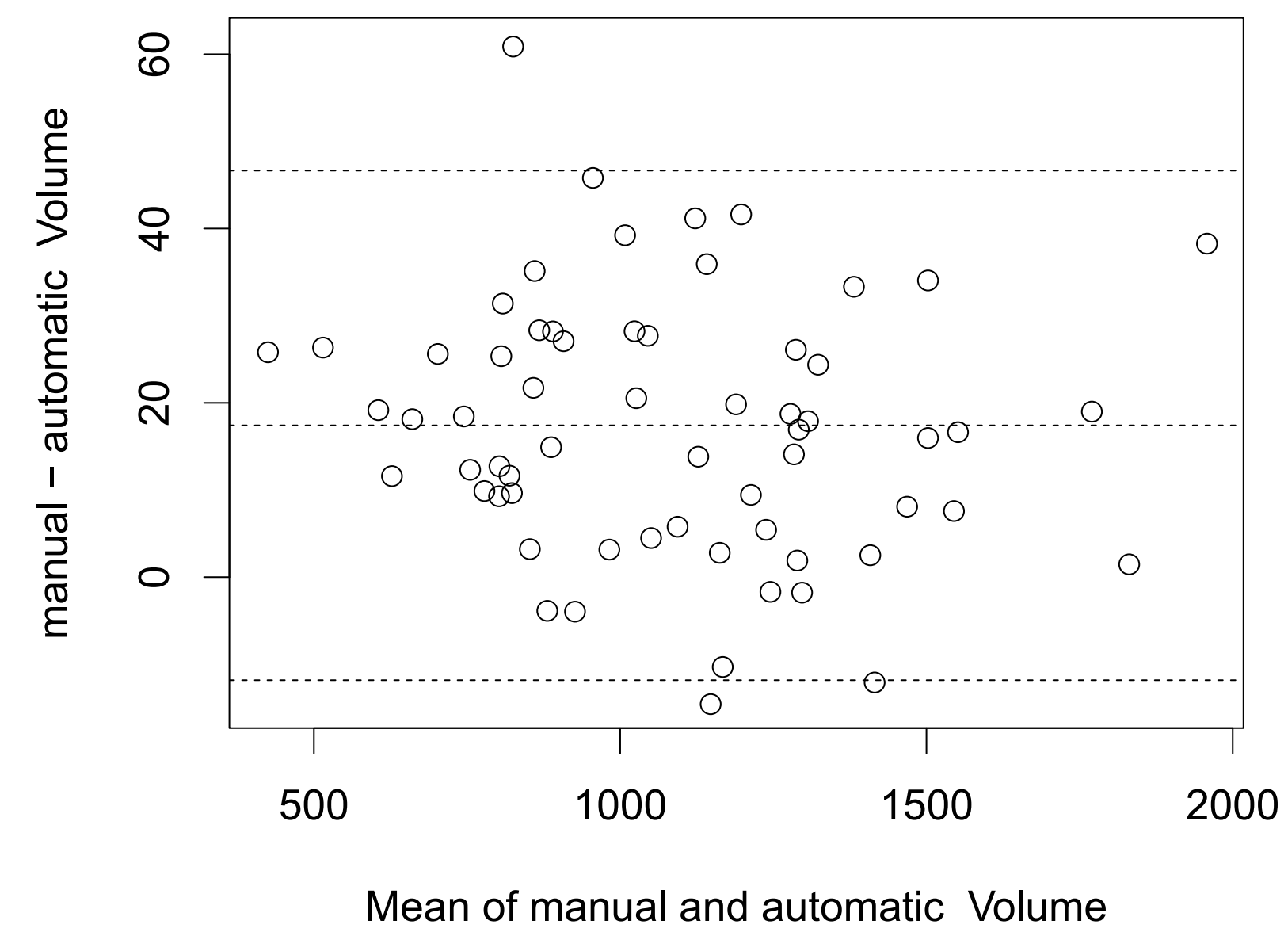**LUL B30f**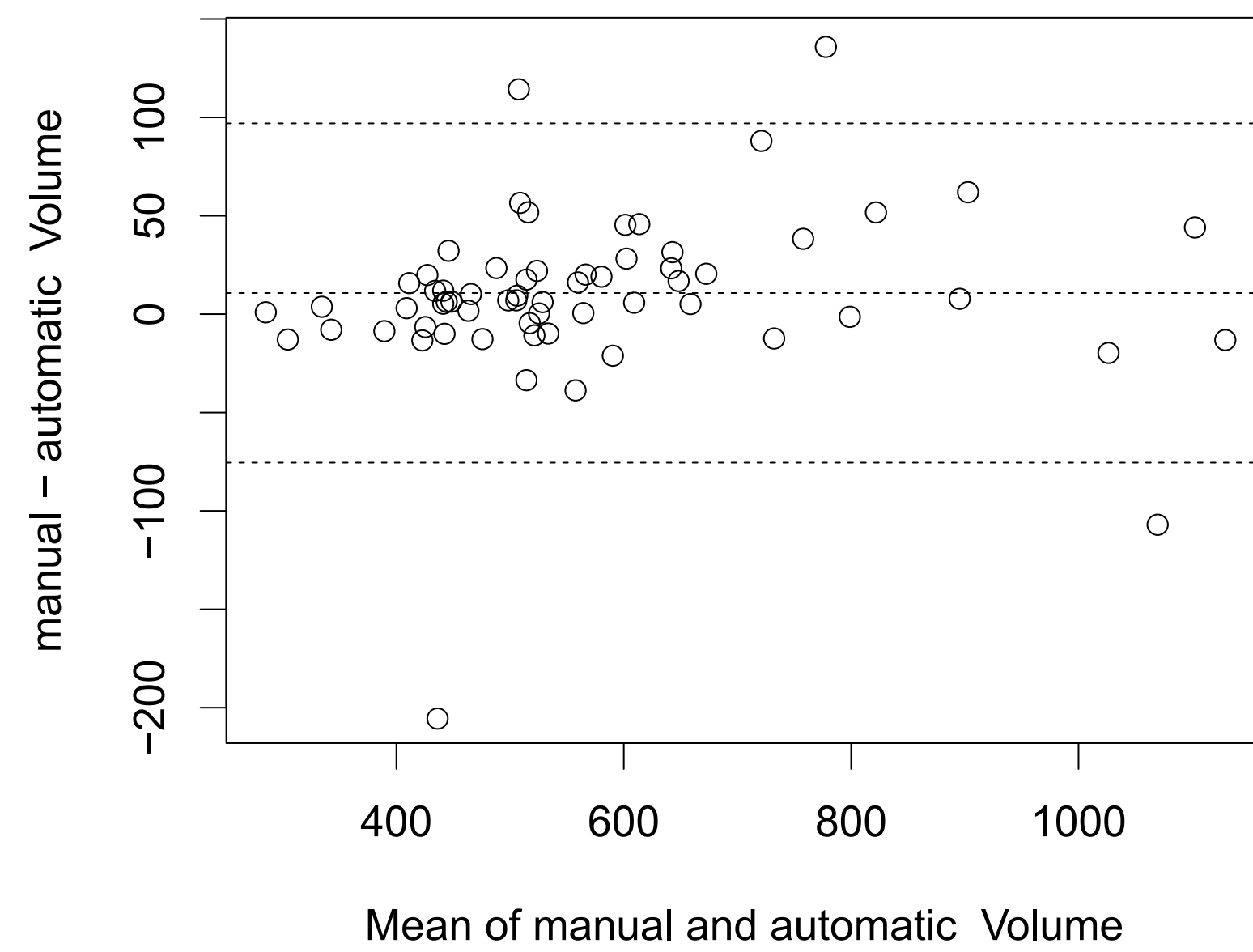**LLi B30f**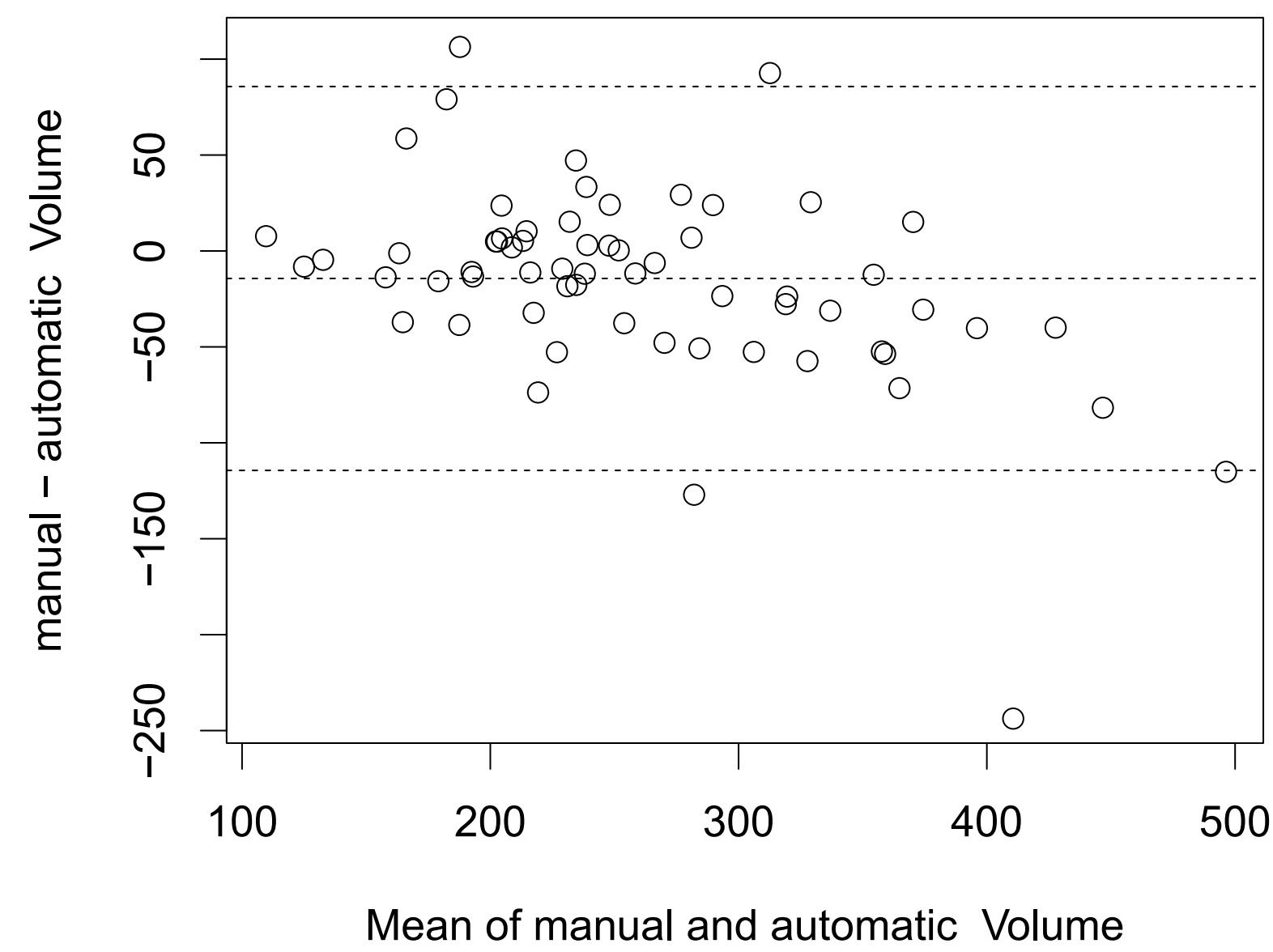**LLL B30f**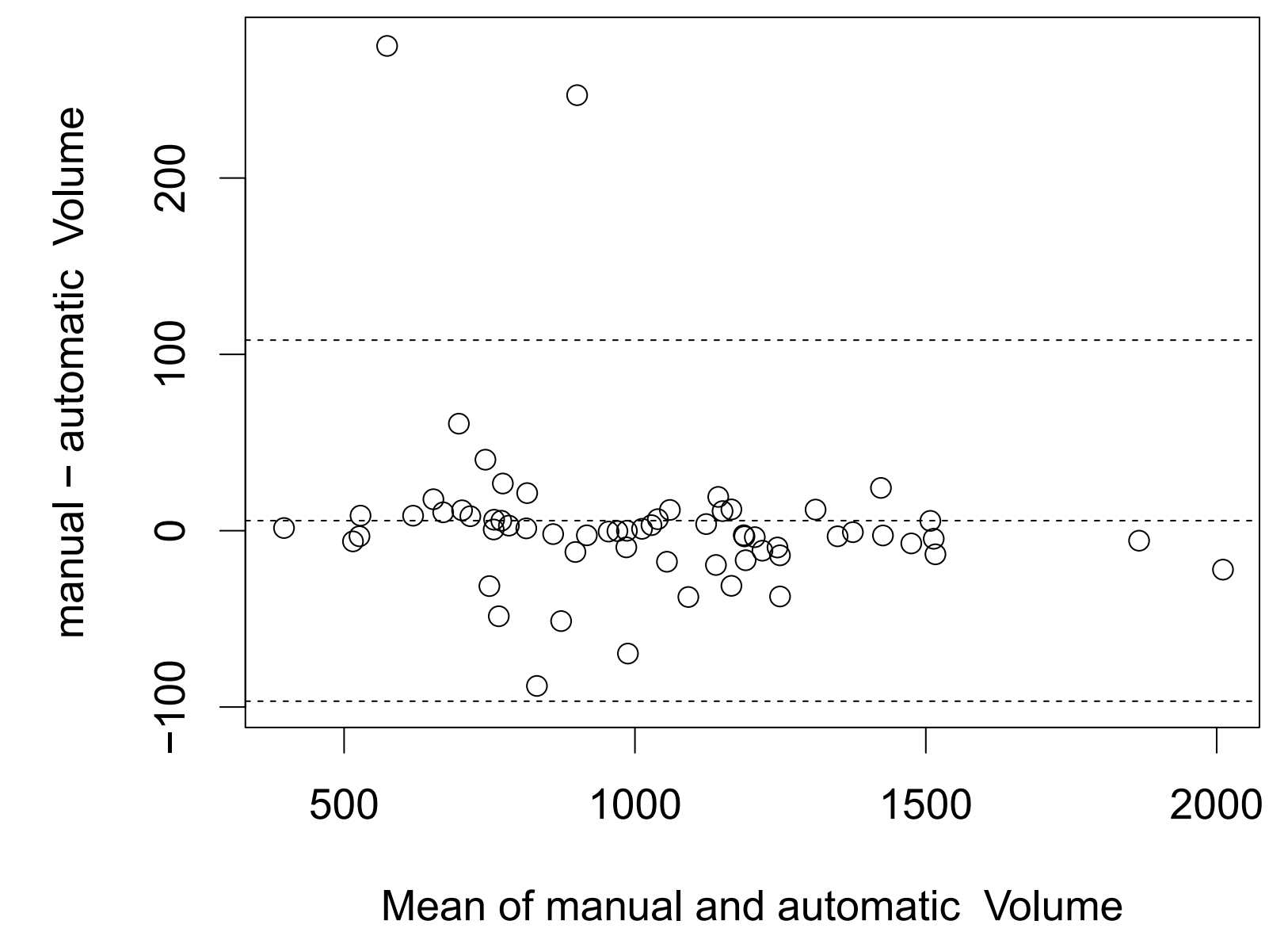**LUL+LLi B30f**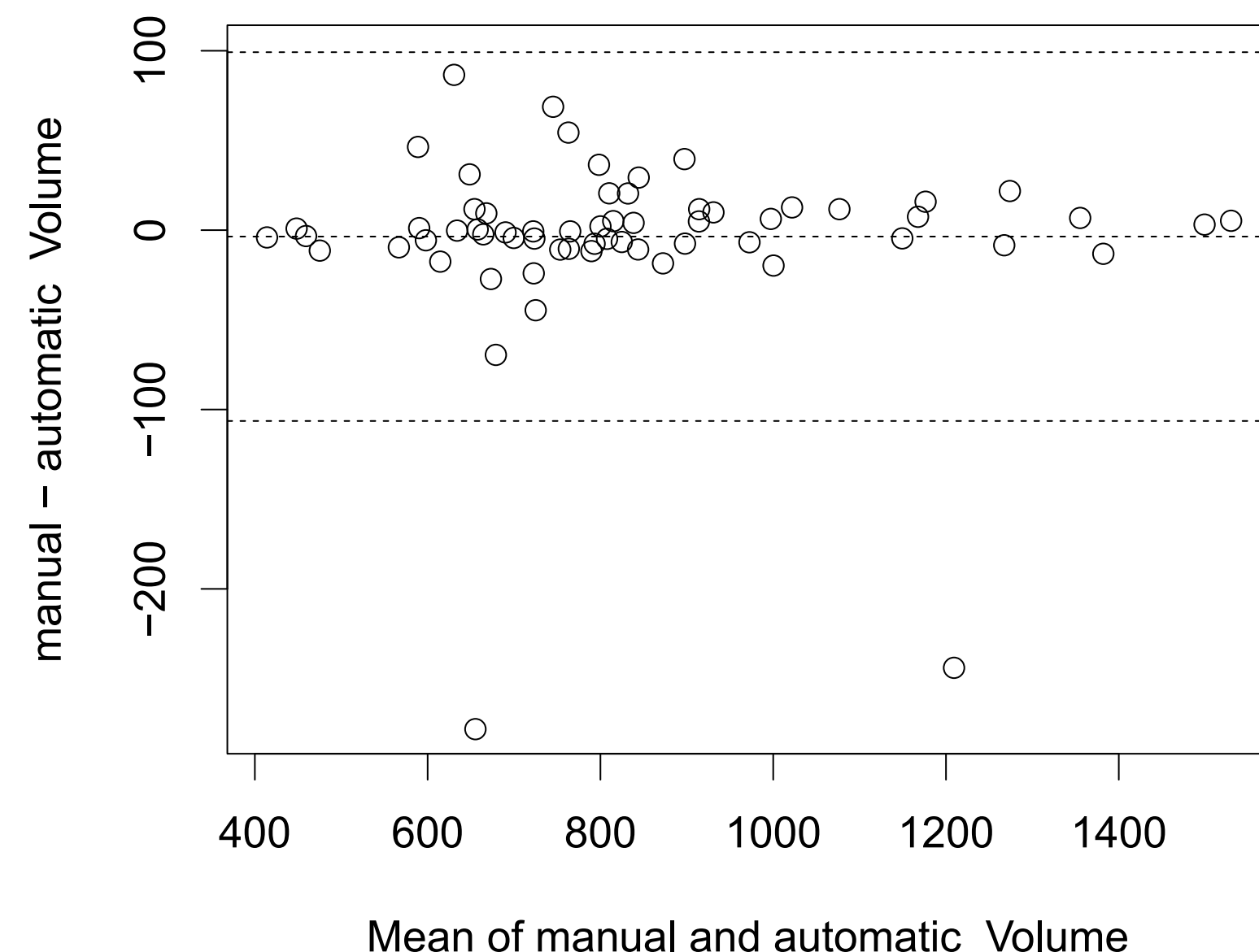

Supplement: S2 Fig — (PDF) [file pone.0194557.s002.pdf]

**RUL B60f**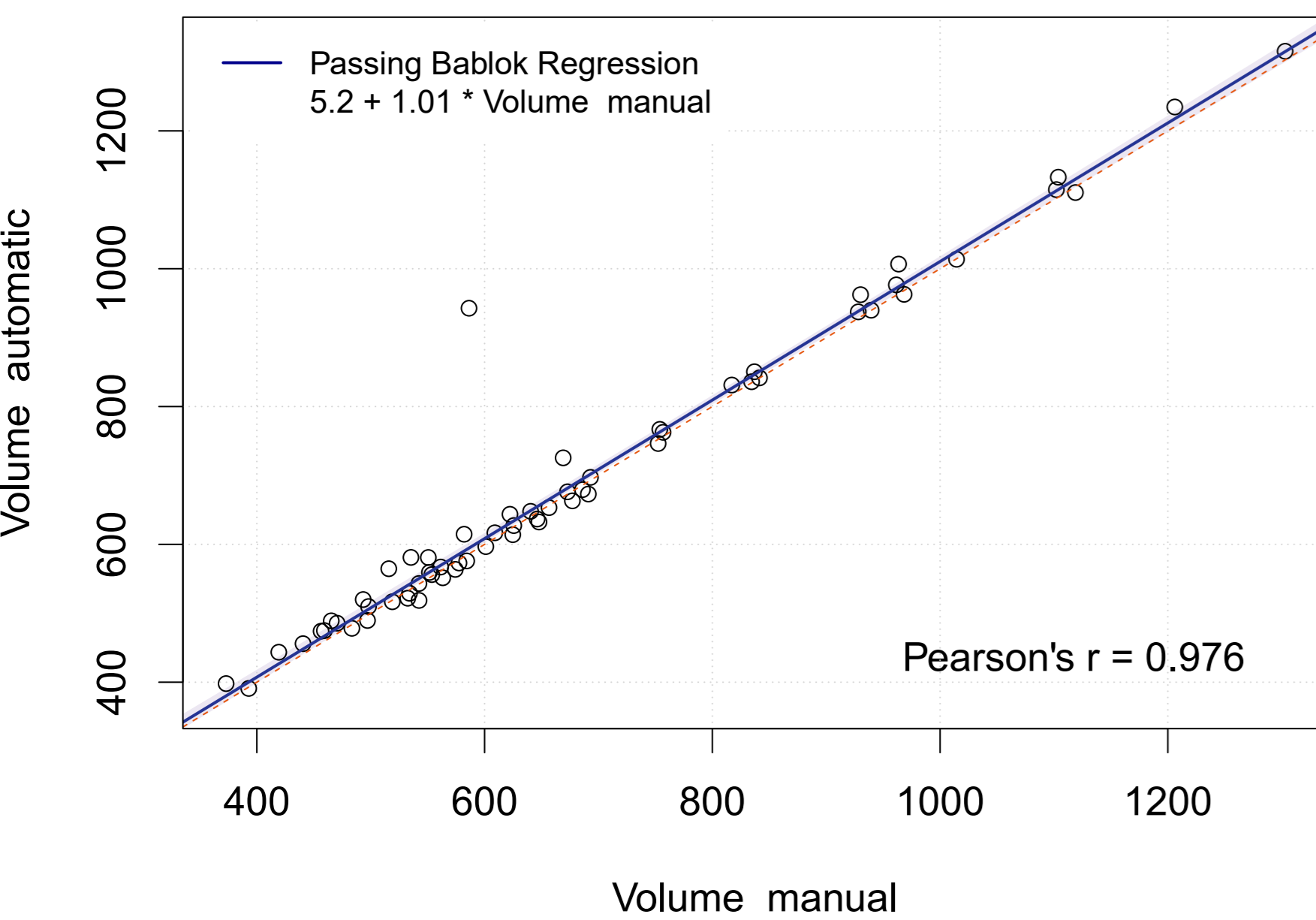**RML B60f**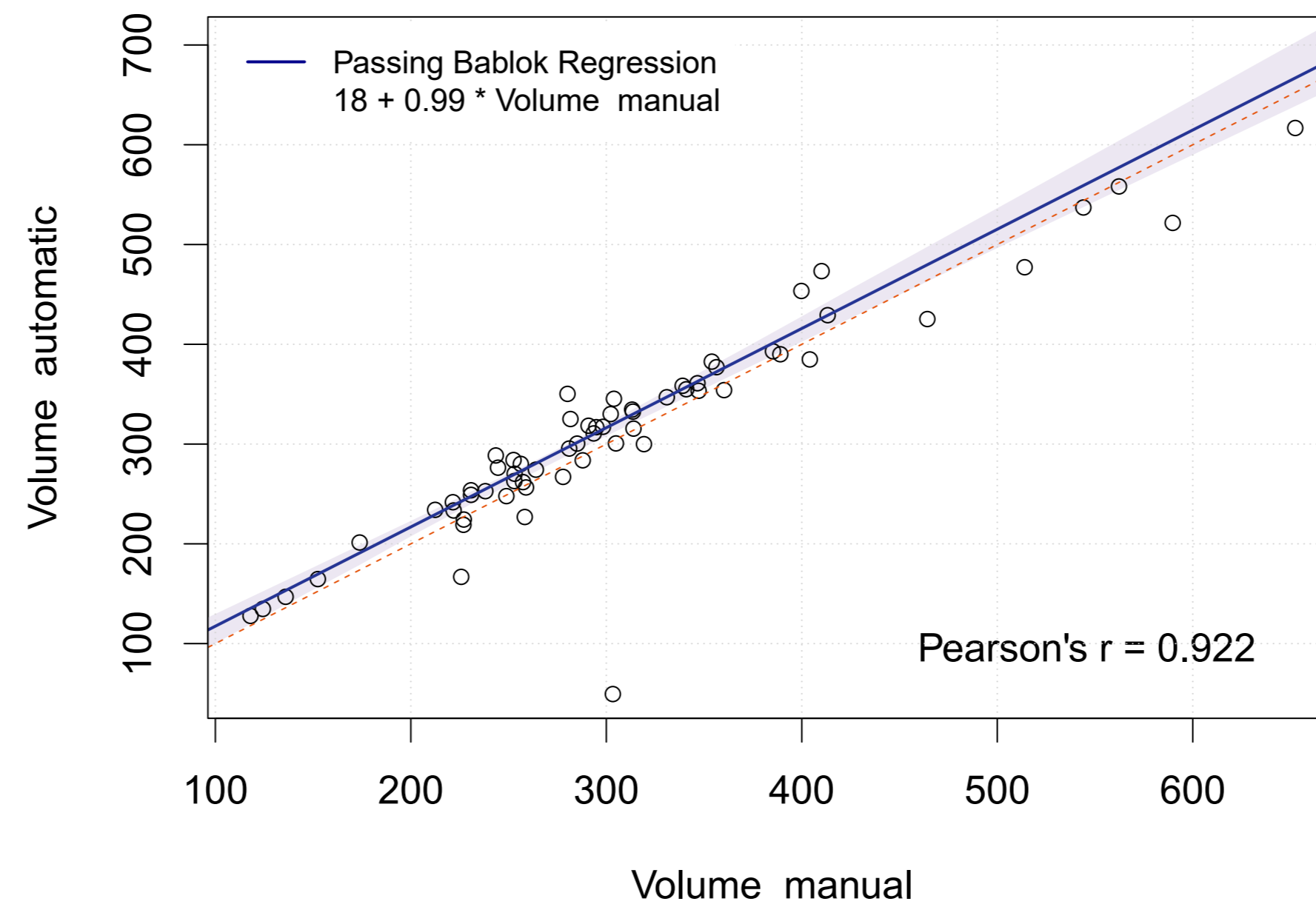**RLL B60f**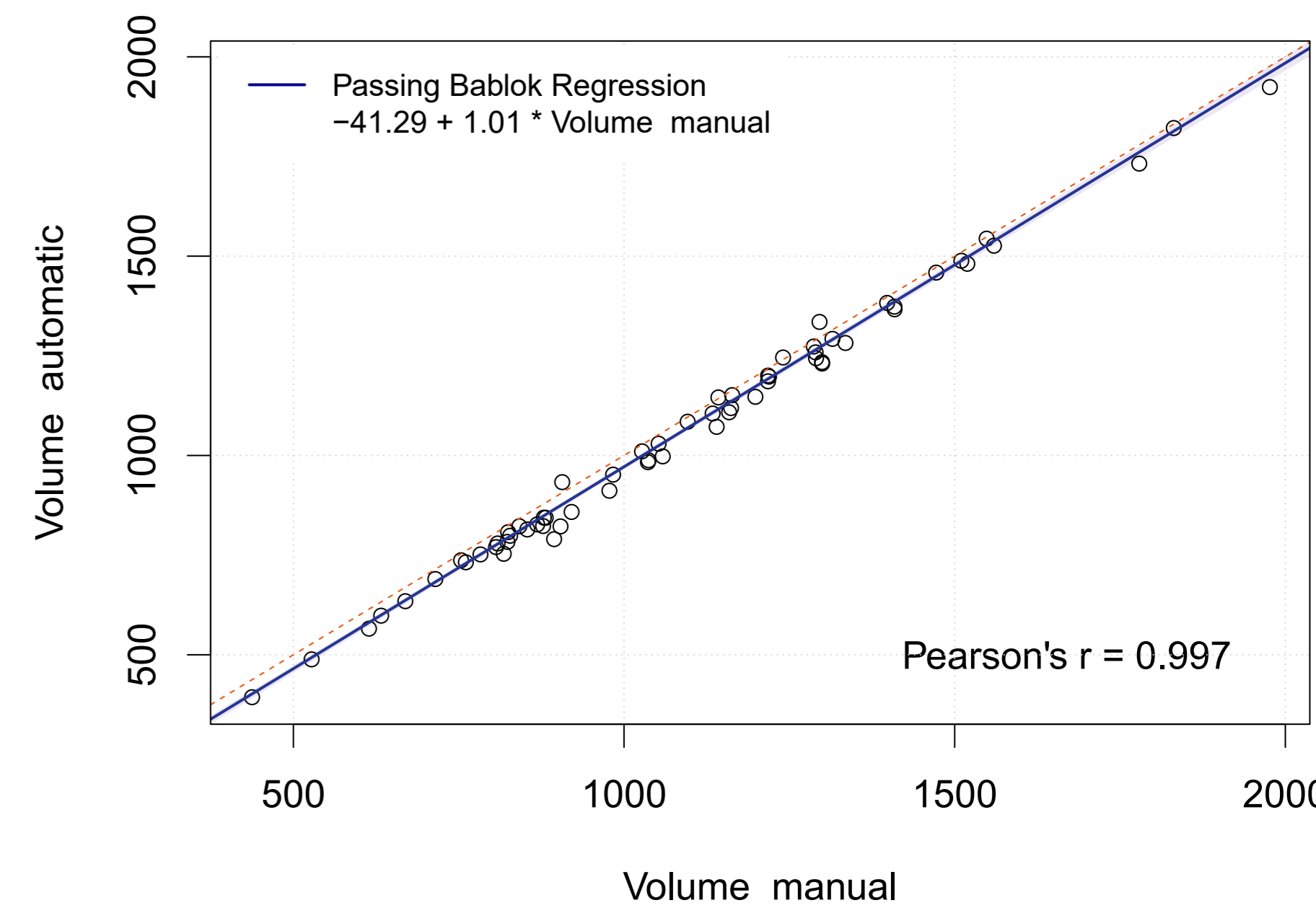**LUL B60f**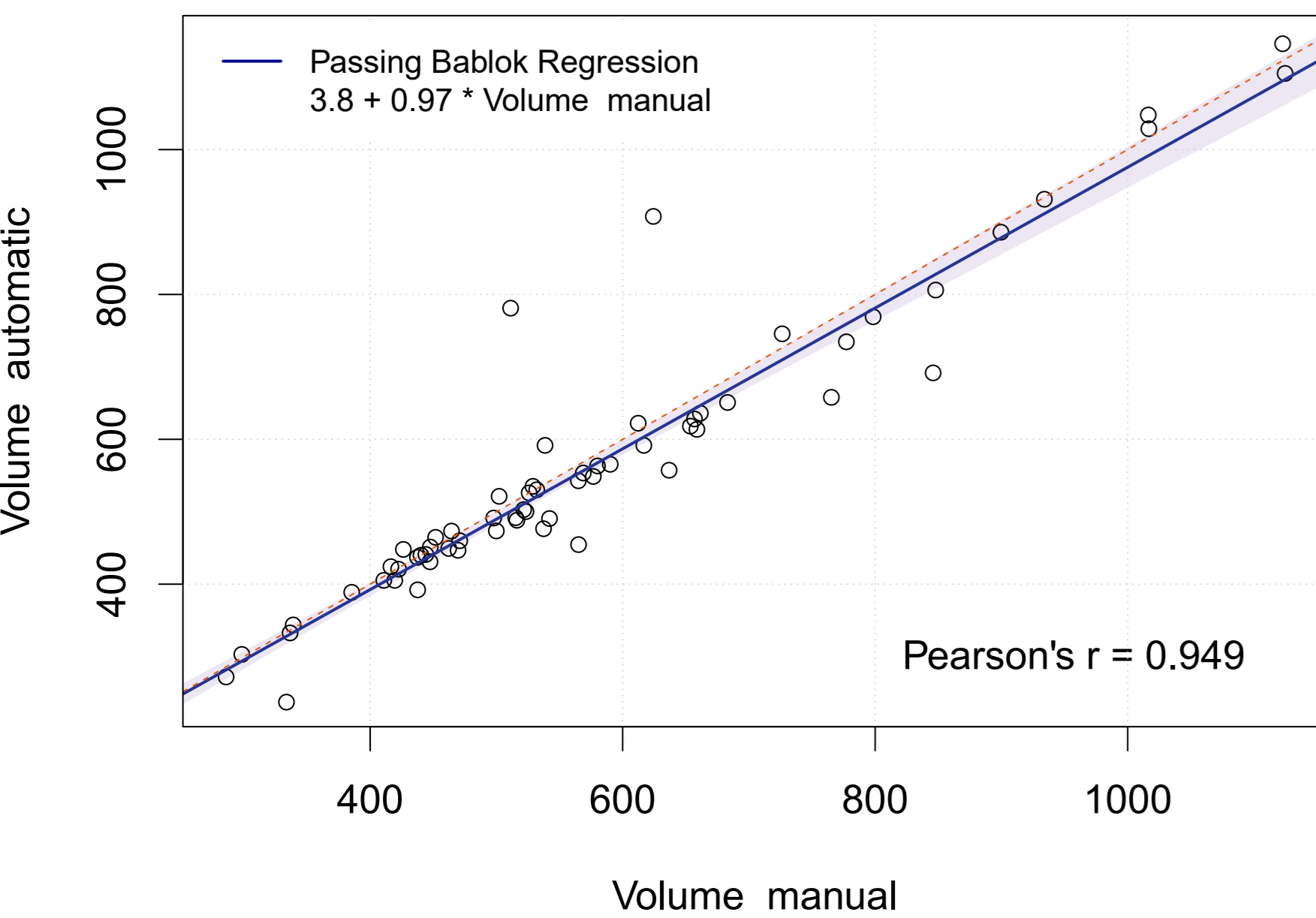**LLi B60f**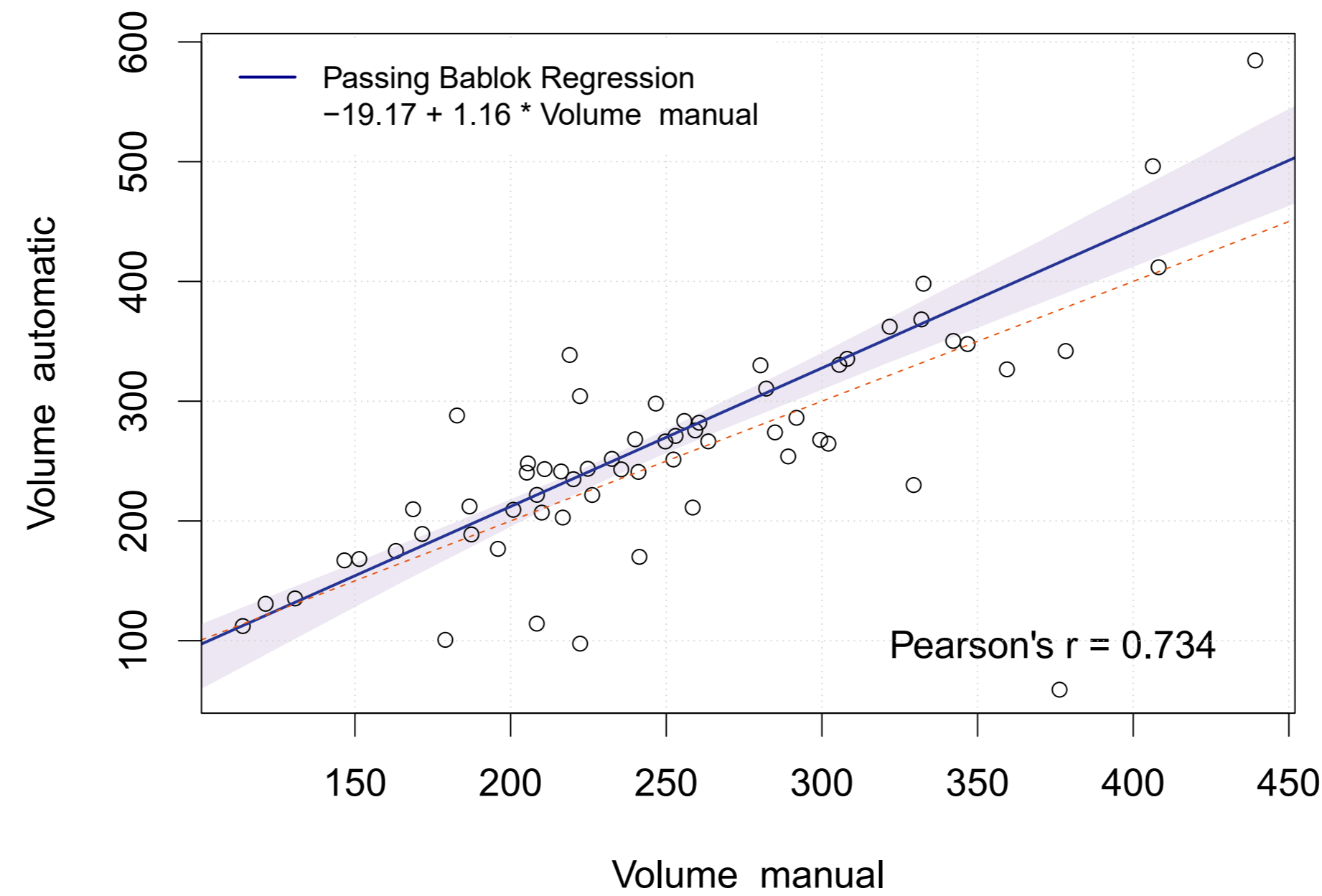**LLL B60f**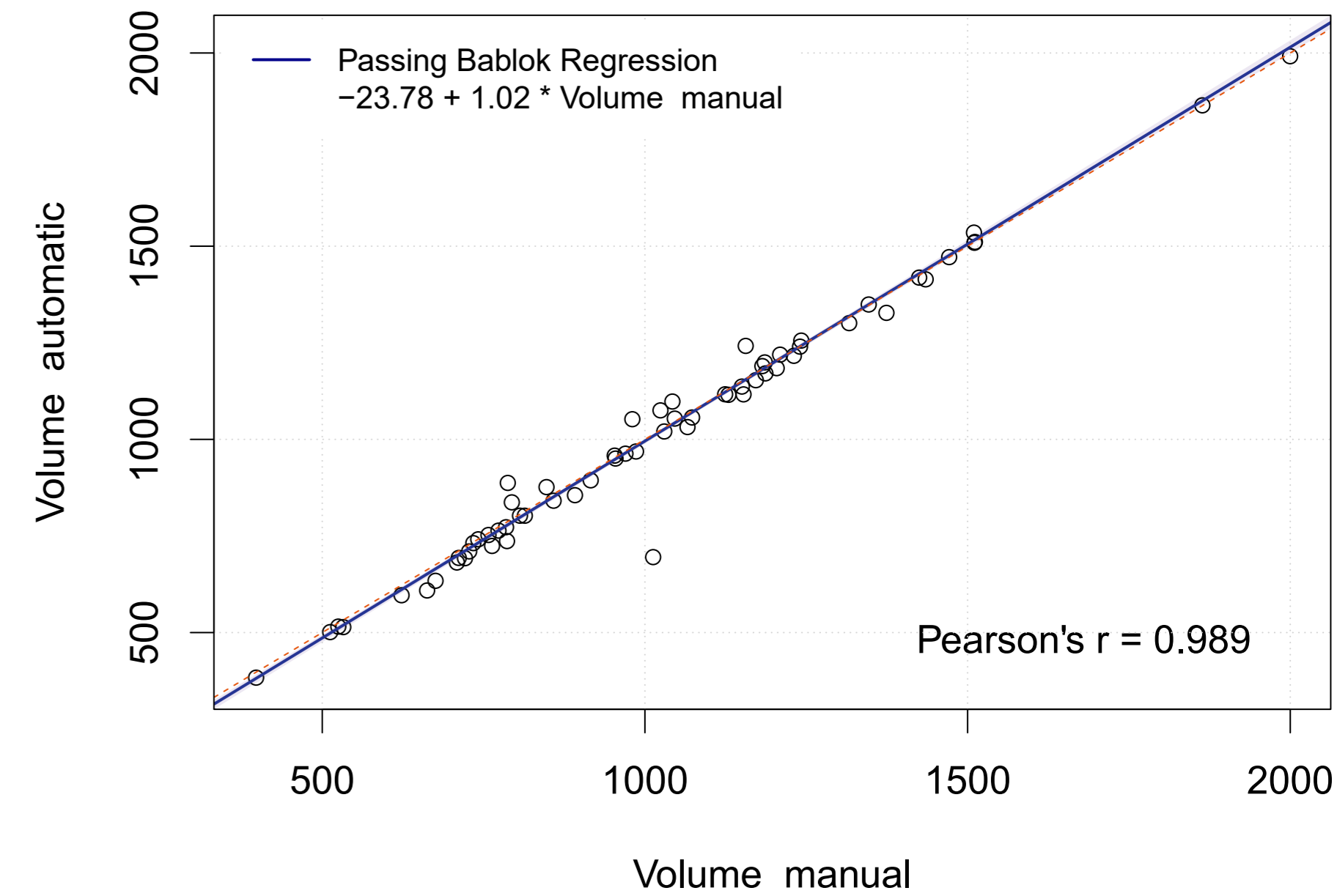**LUL+LLi B60f**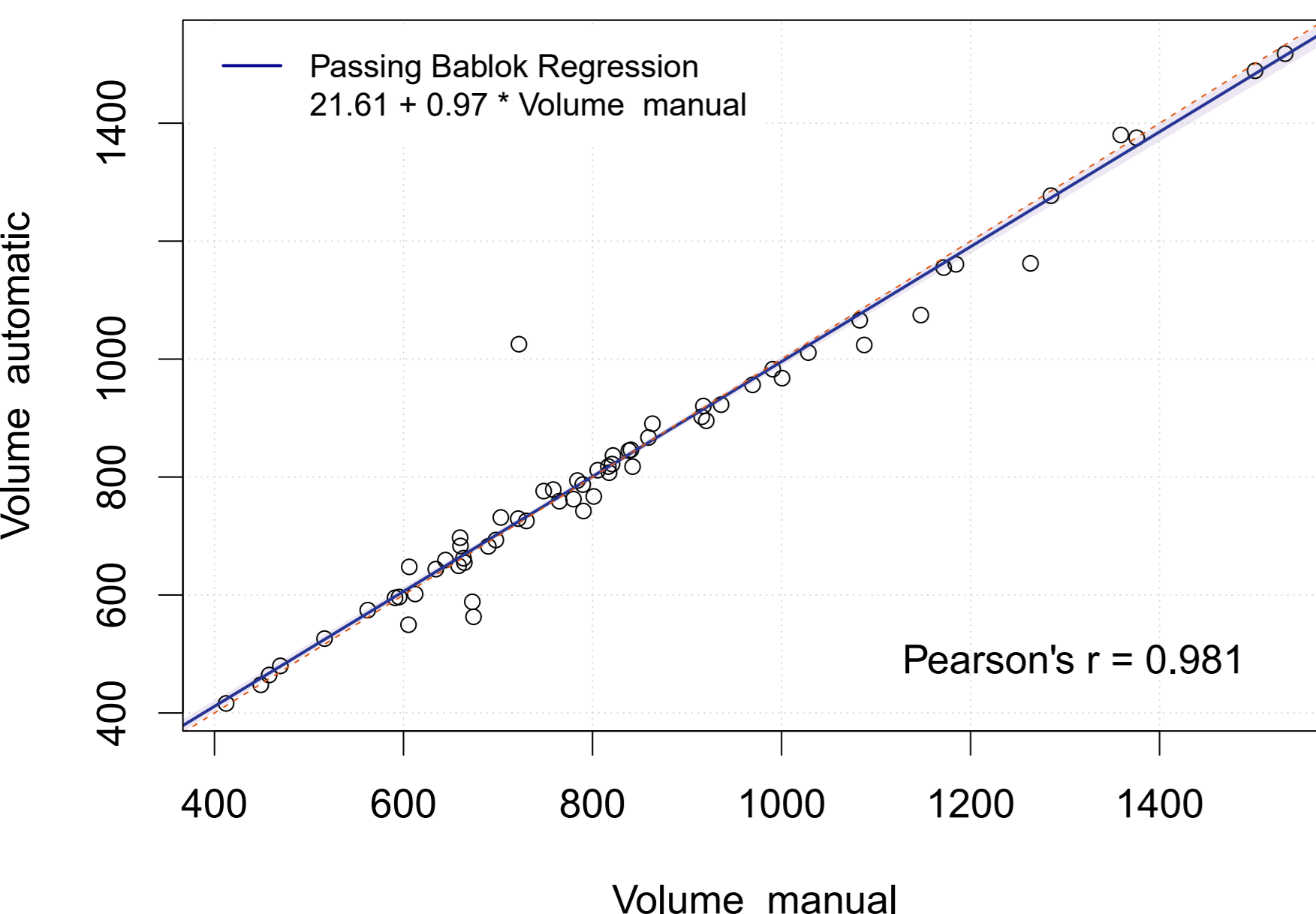

Supplement: S3 Fig — (PDF) [file pone.0194557.s003.pdf]

**RUL B60f**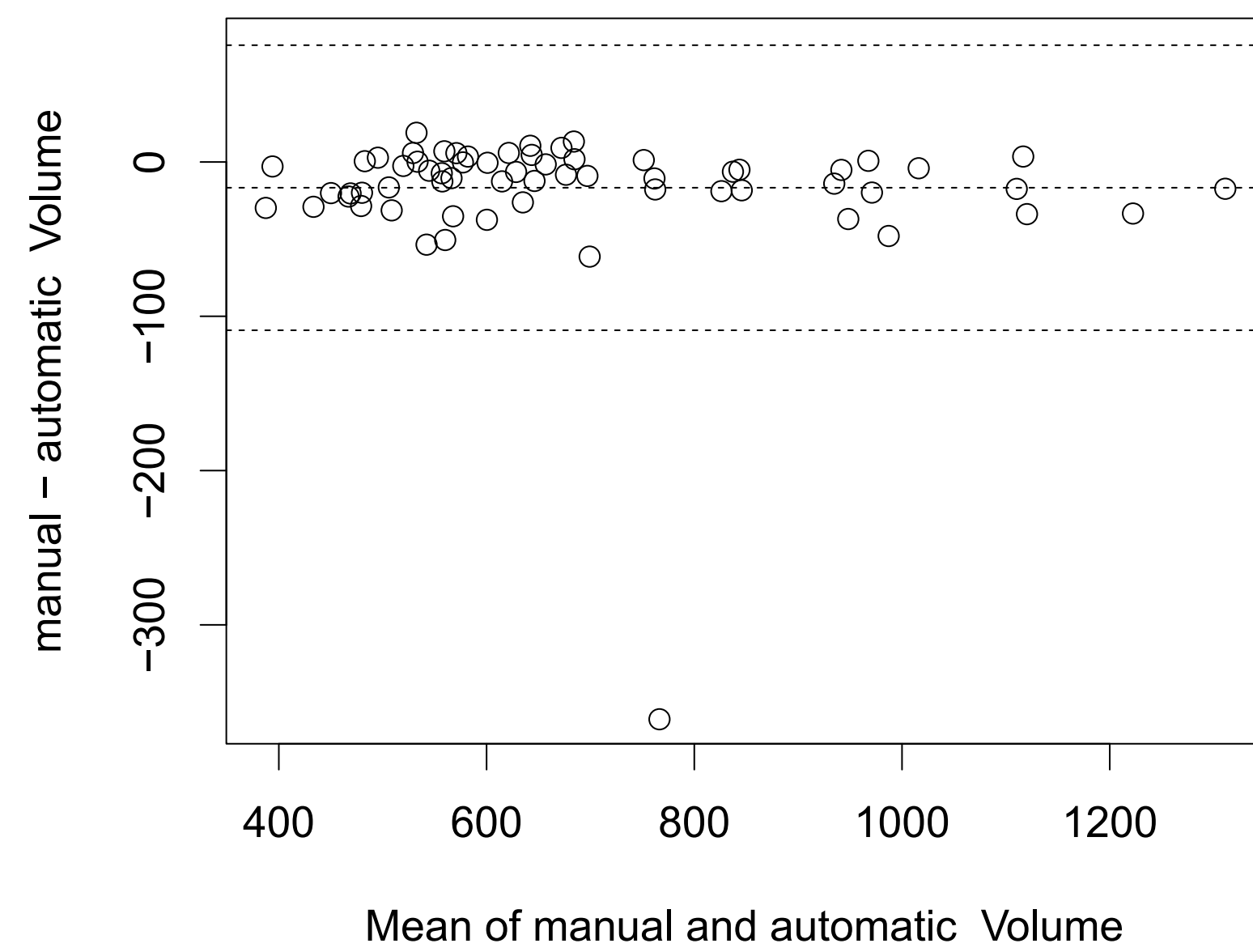**RML B60f**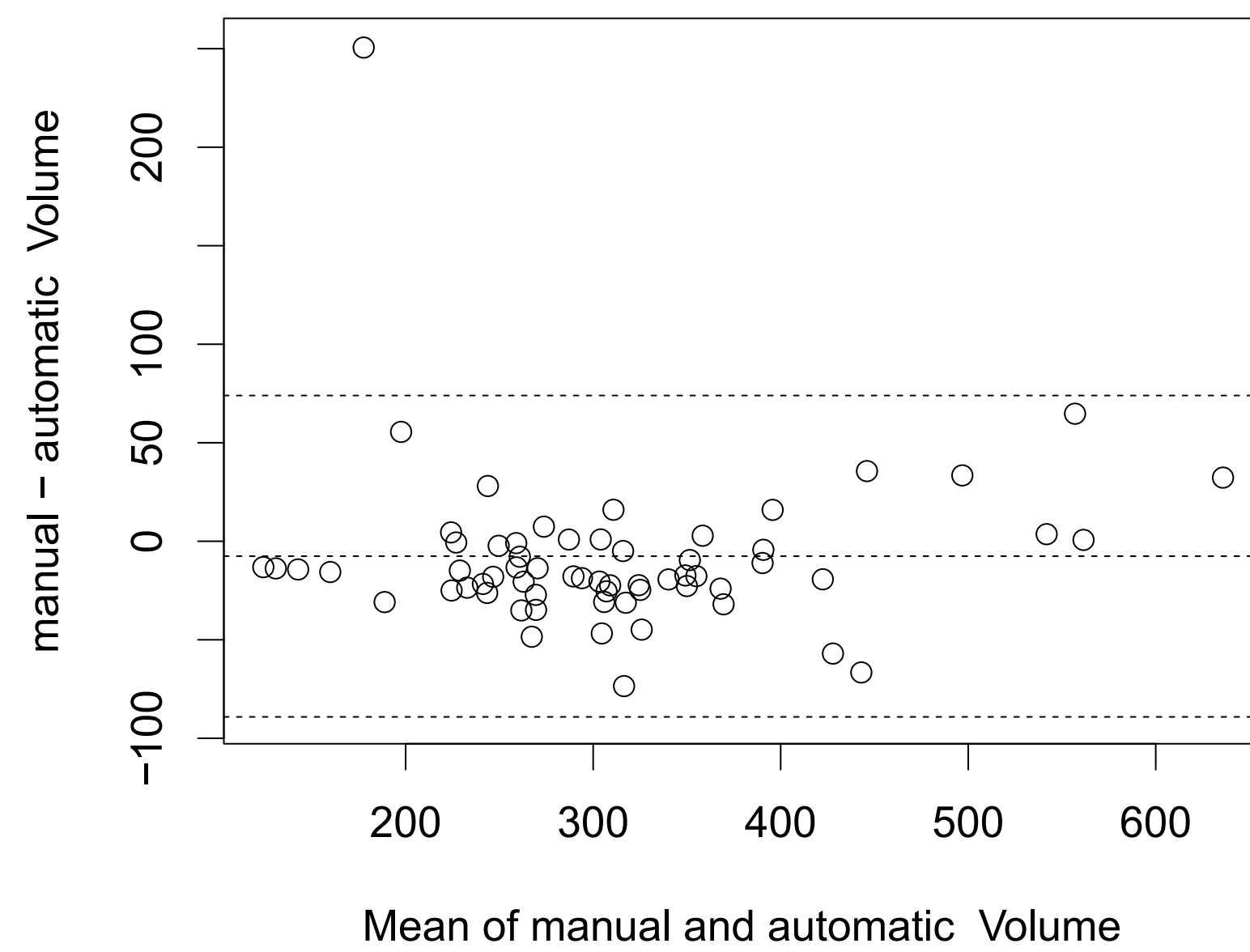**RLL B60f**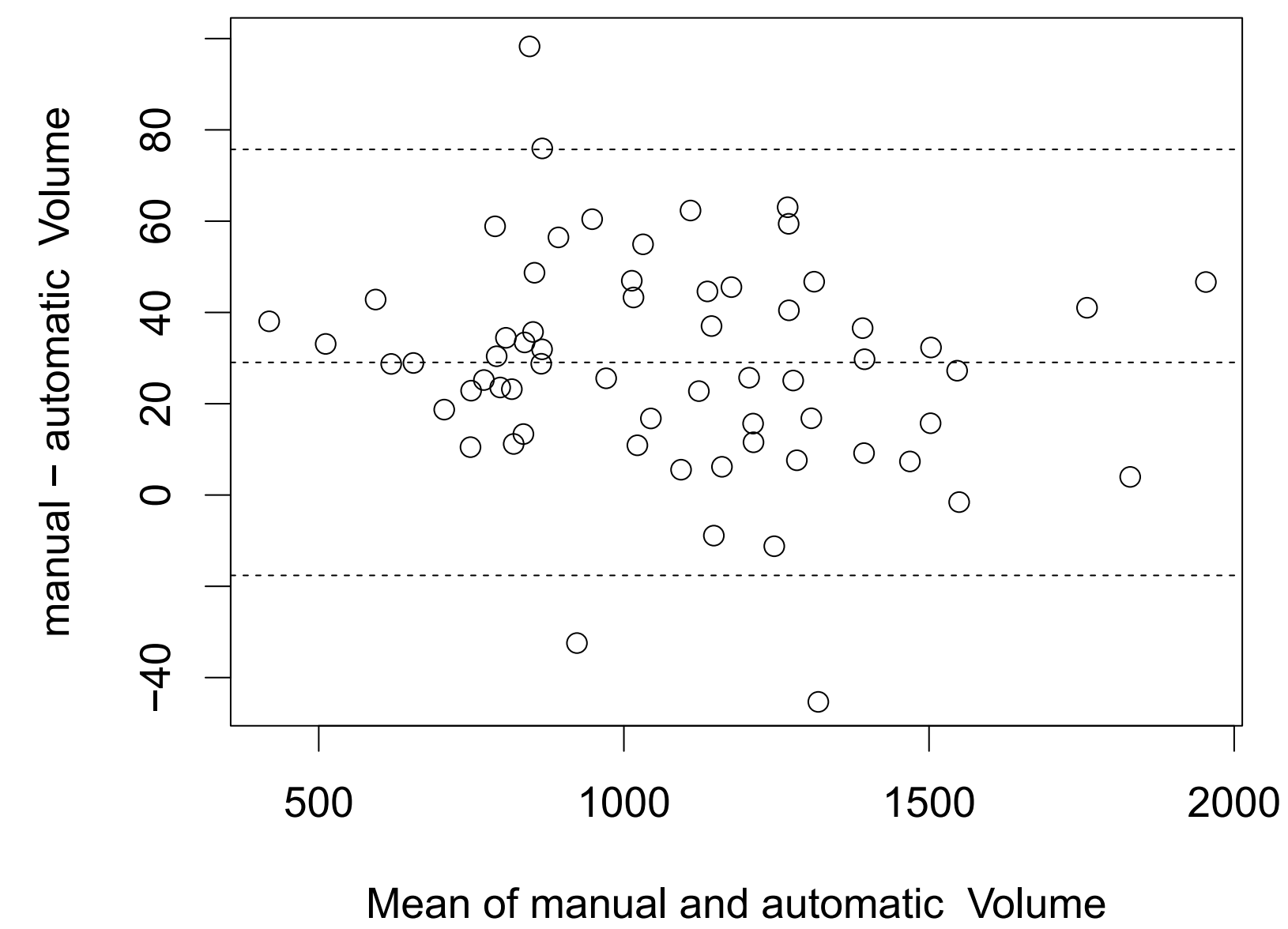**LUL B60f**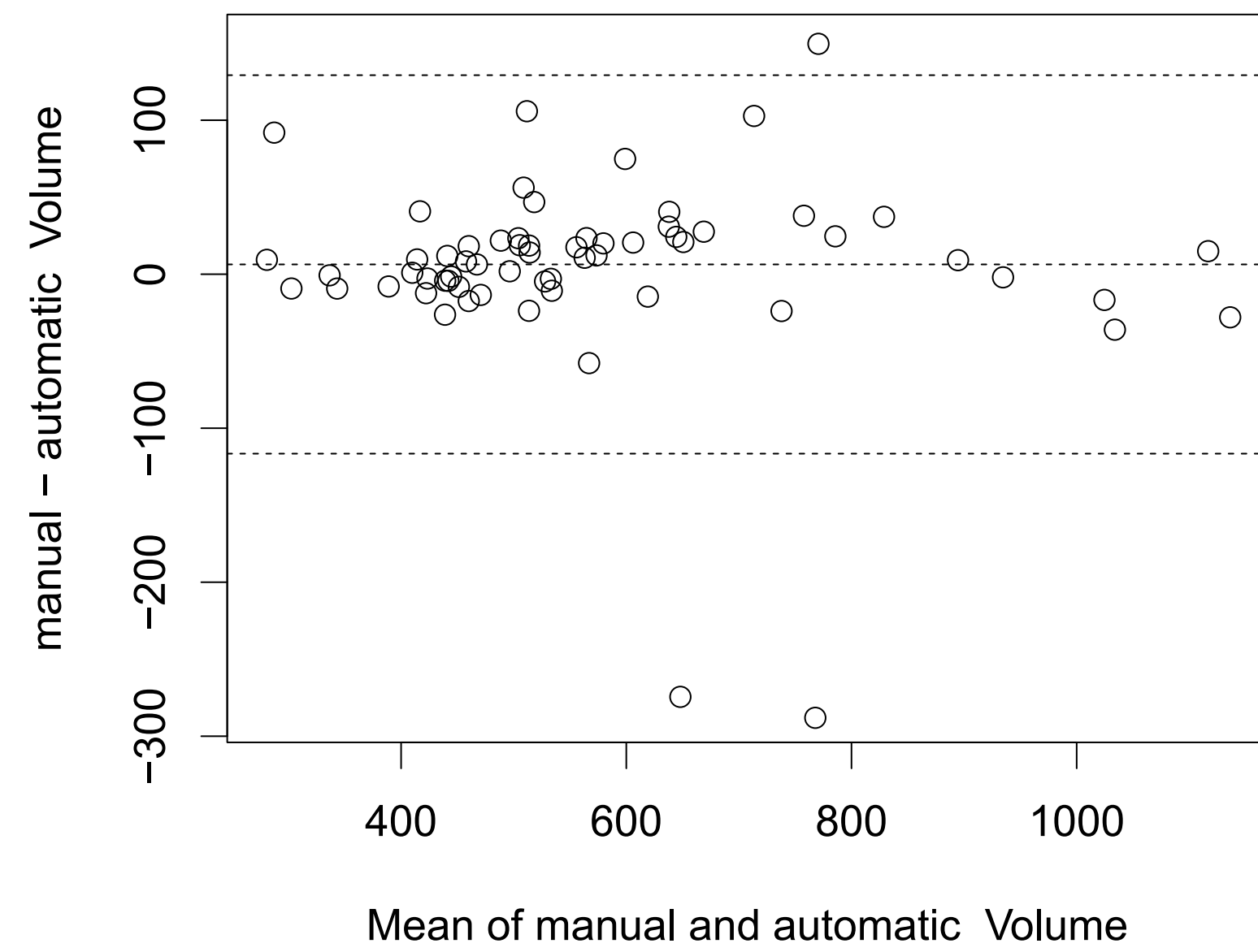**LLi B60f**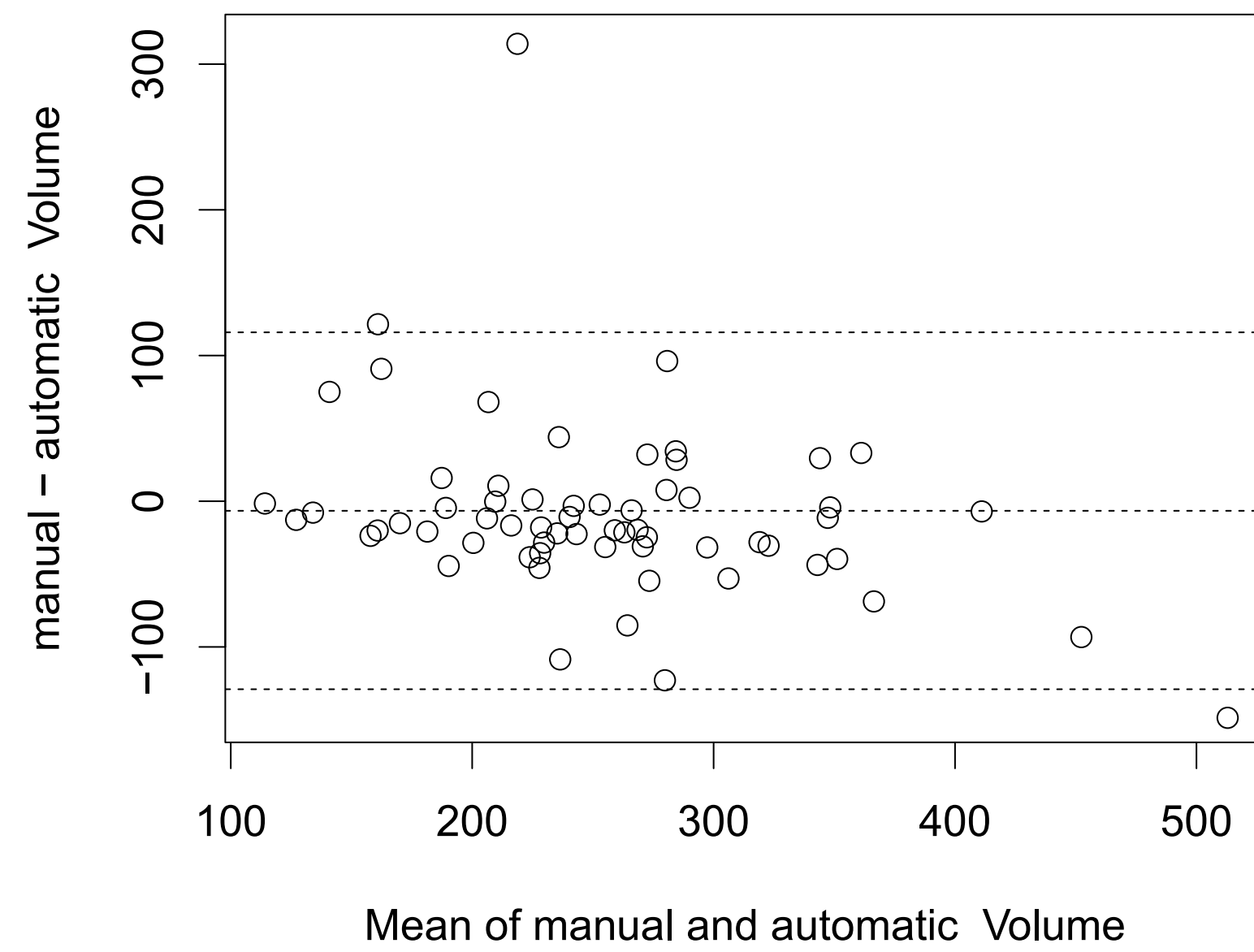**LLL B60f**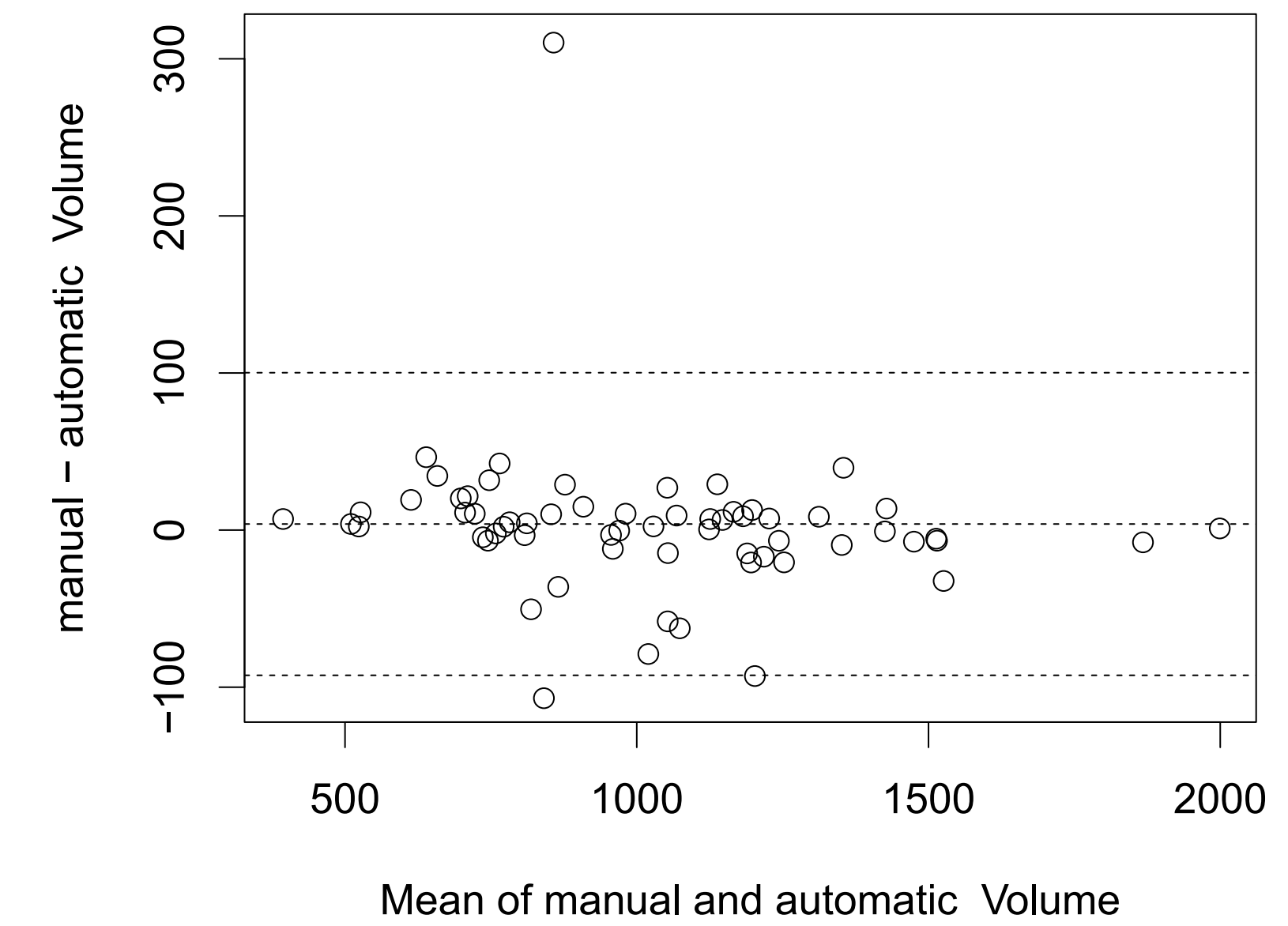**LUL+LLi B60f**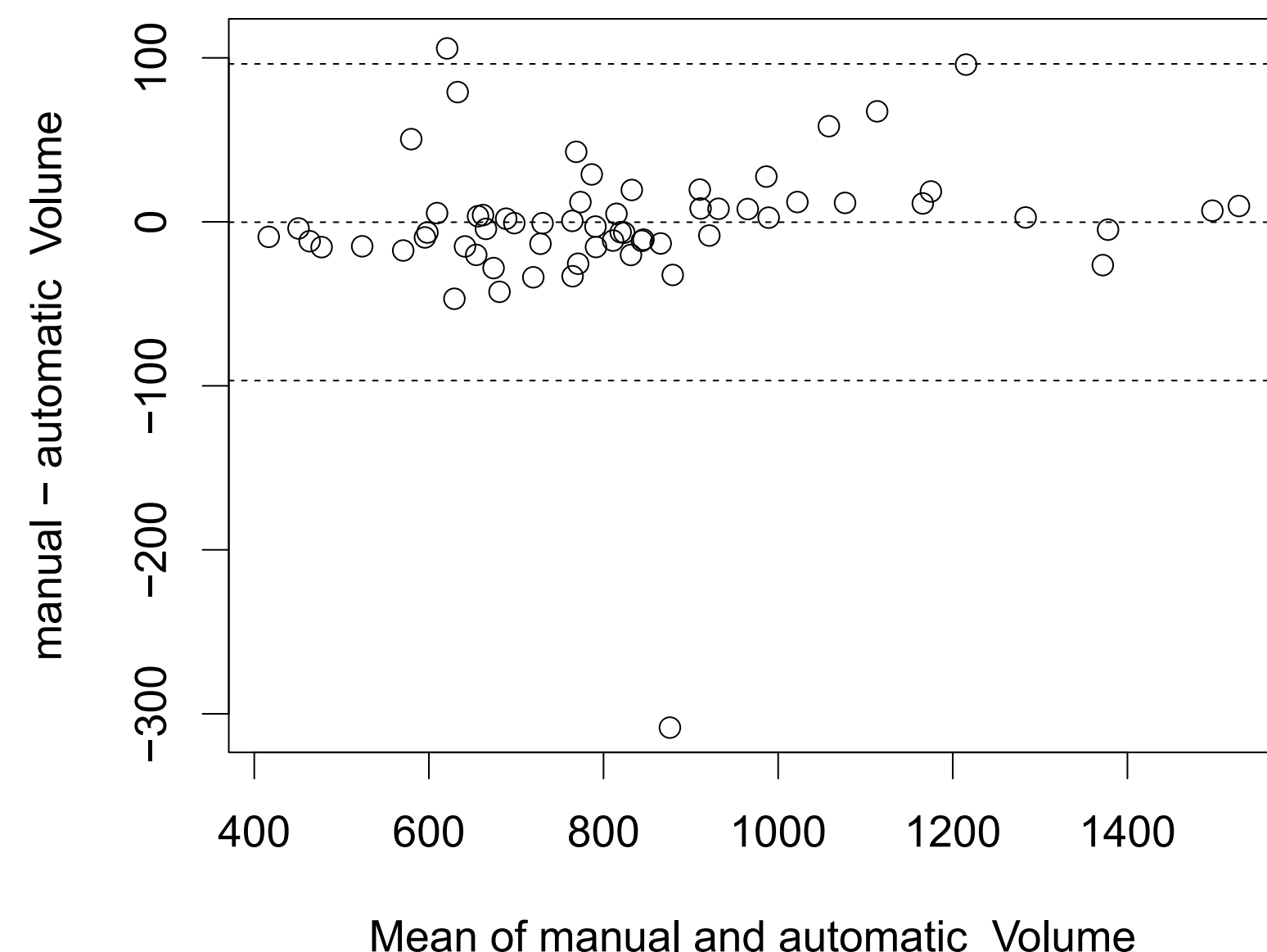

Supplement: S4 Fig — (PDF) [file pone.0194557.s004.pdf]

**RUL B30f**

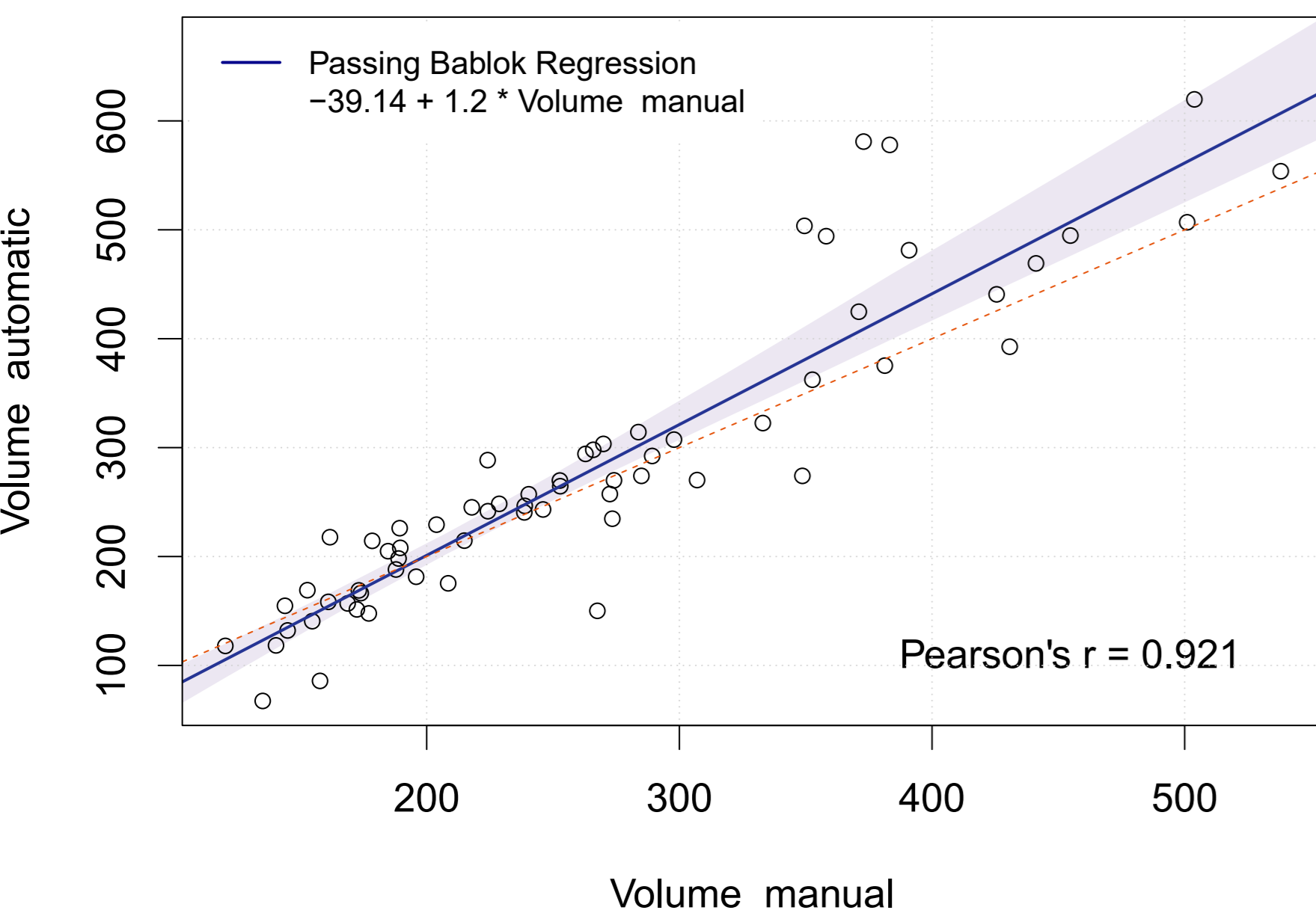

**RML B30f**

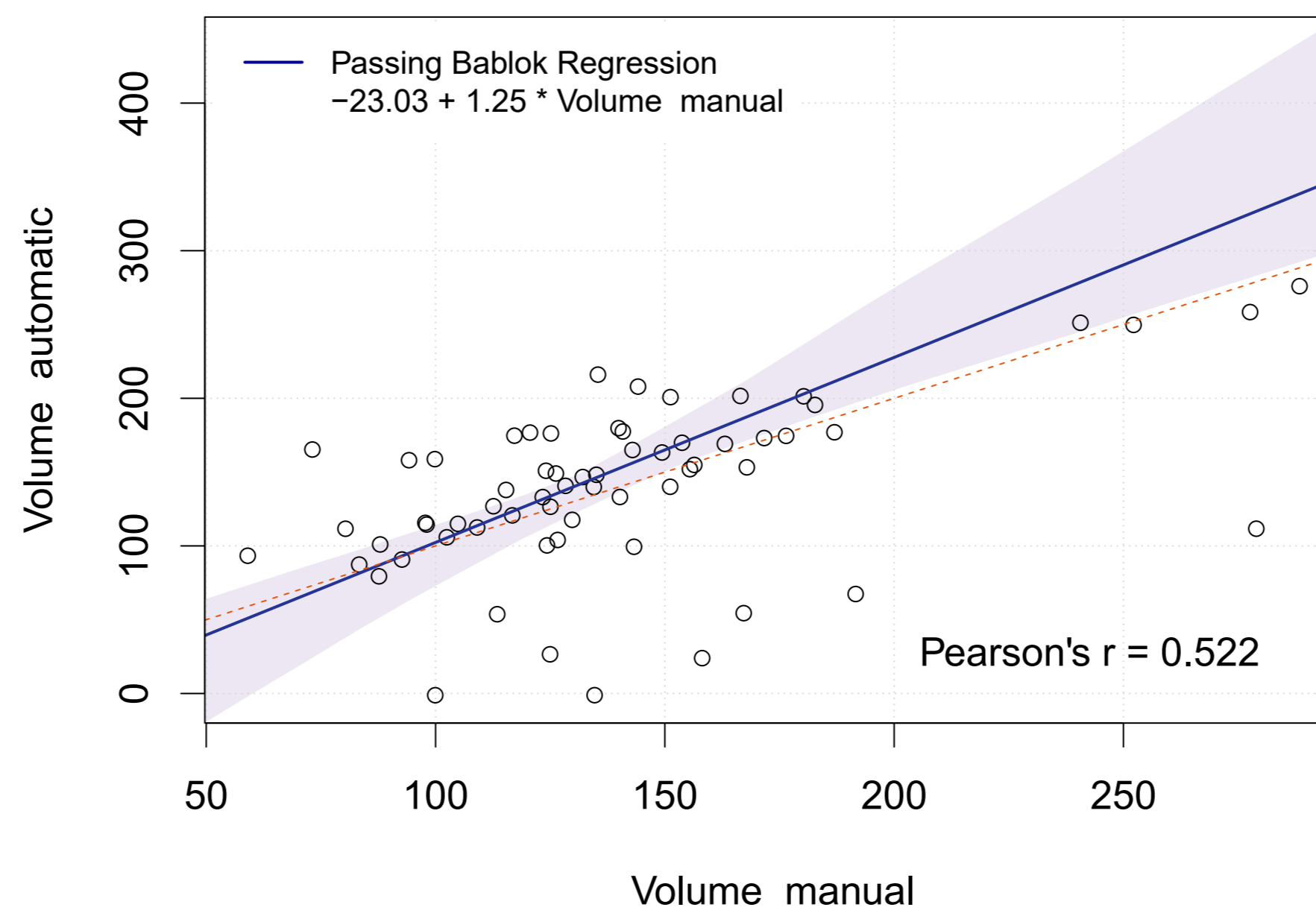

**RLL B30f**

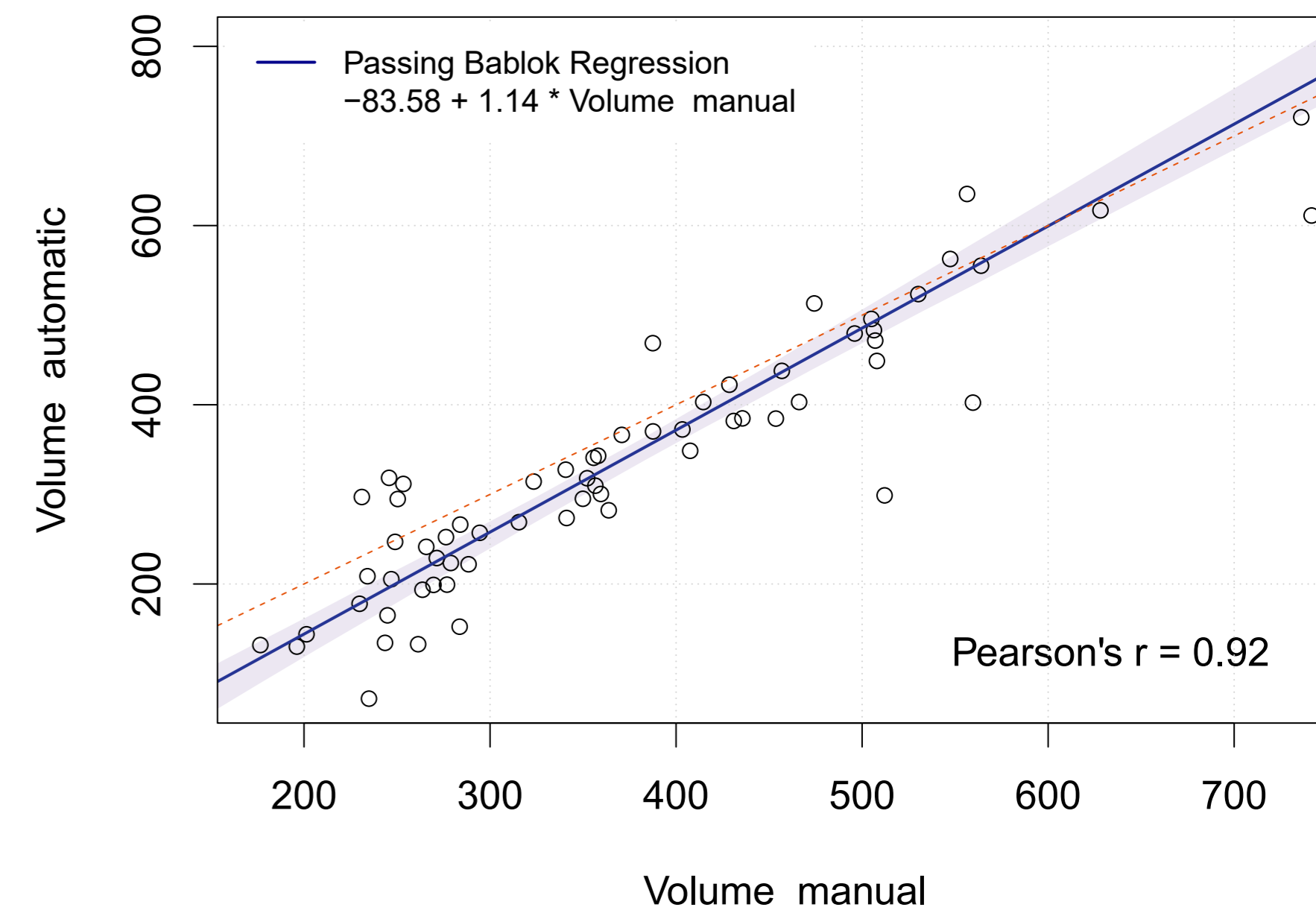

**LUL B30f**

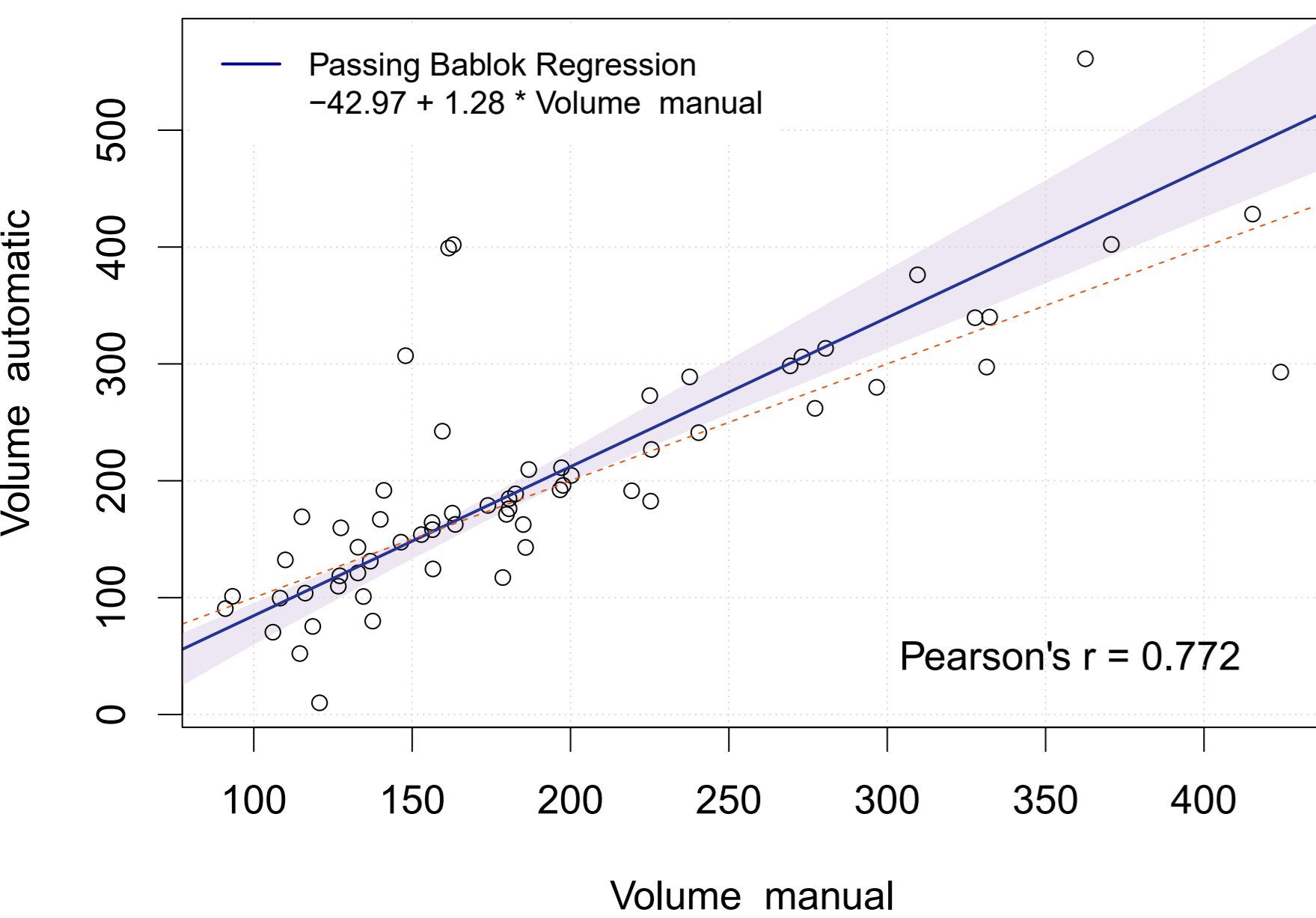

**LLi B30f**

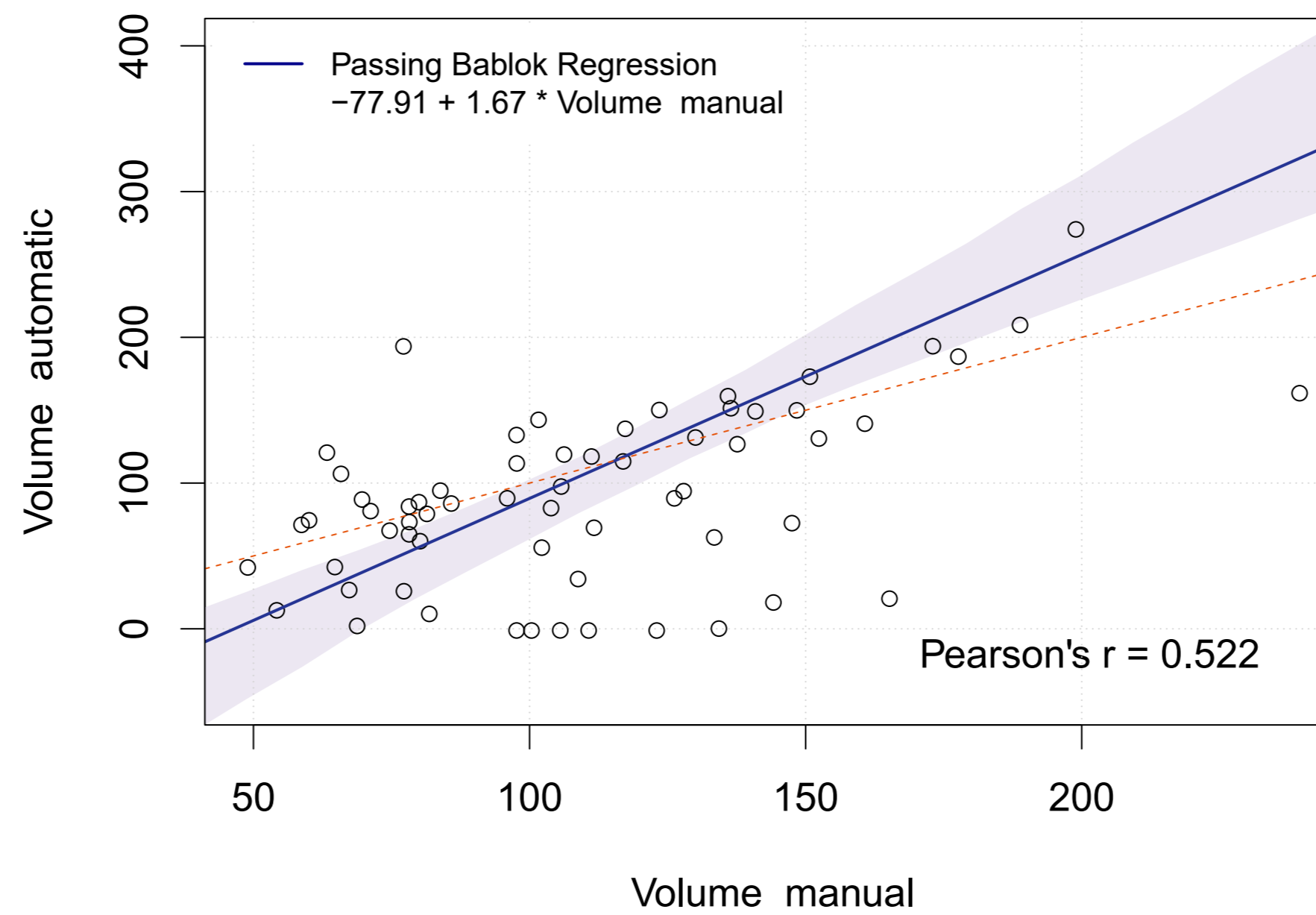

**LLL B30f**

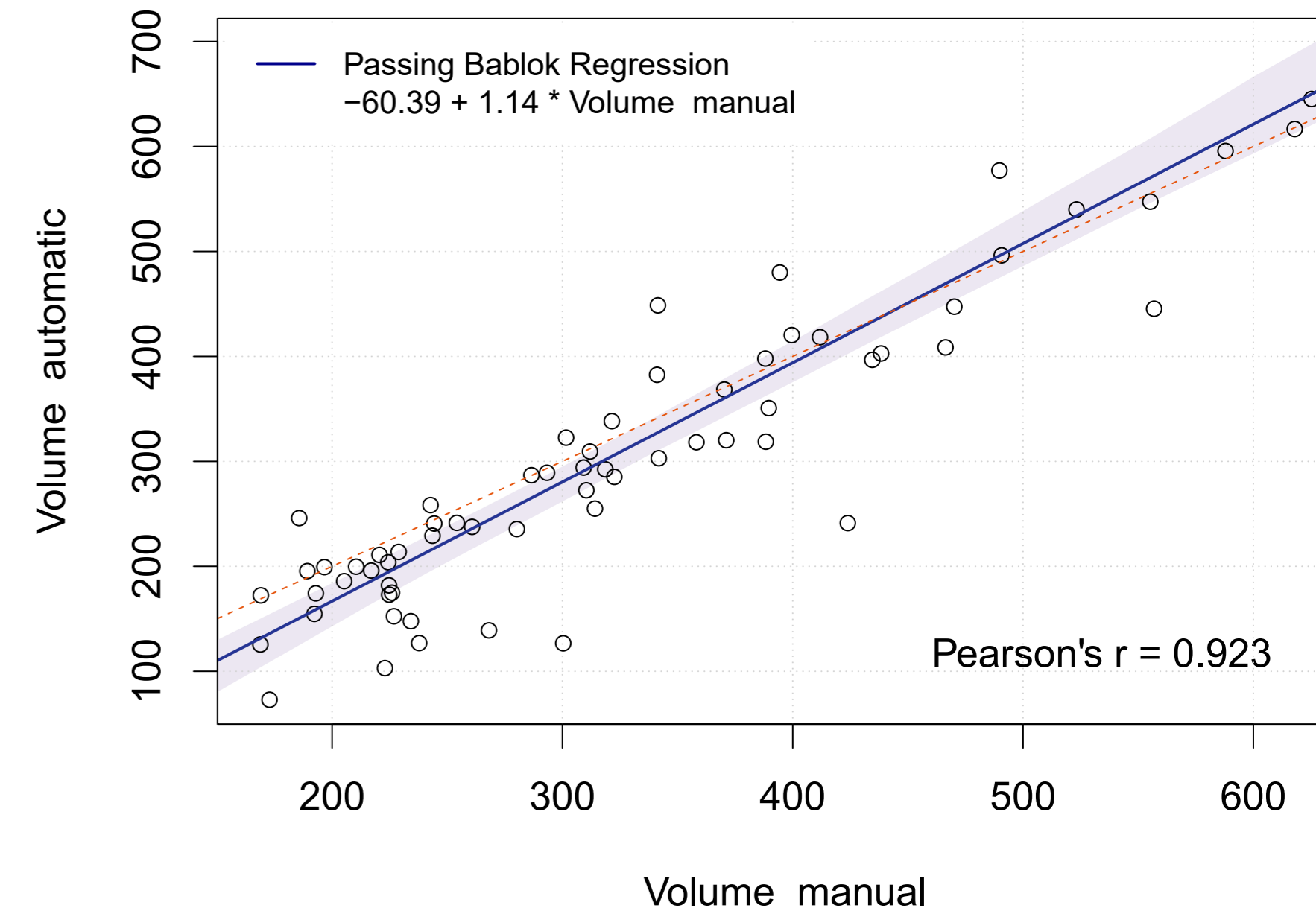

**LUL+LLi B30f**

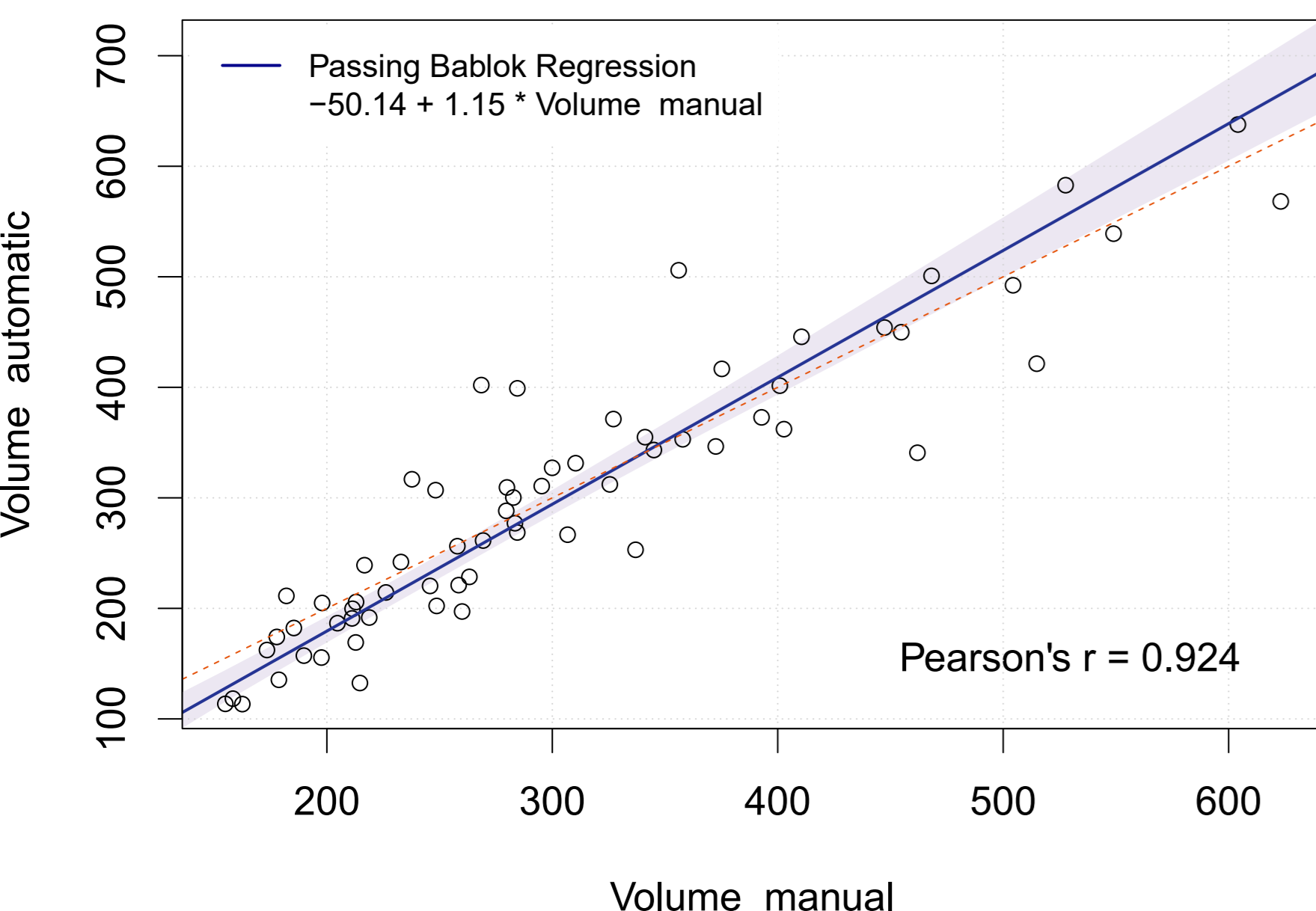

Supplement: S5 Fig — (PDF) [file pone.0194557.s005.pdf]

**RUL B30f**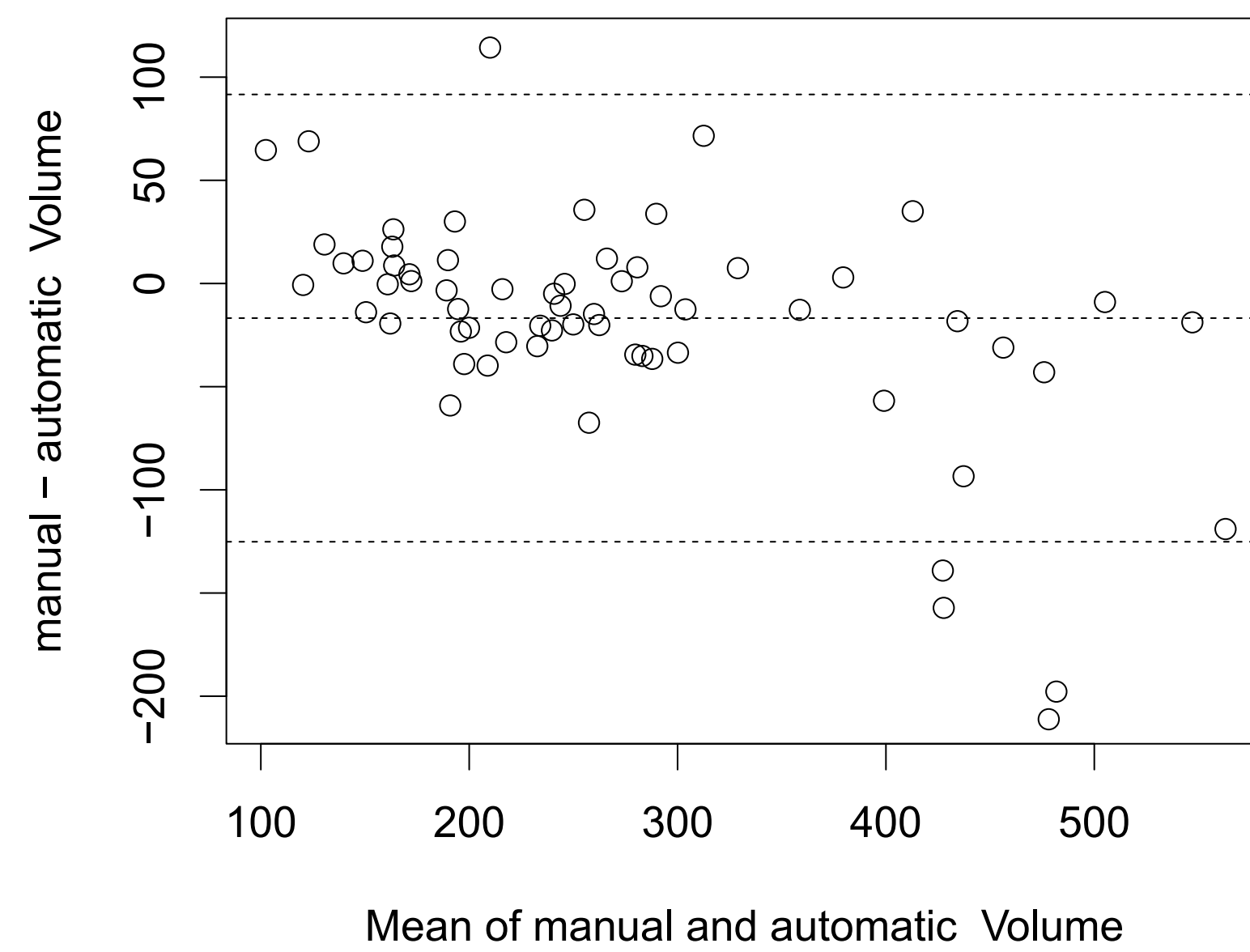**RML B30f**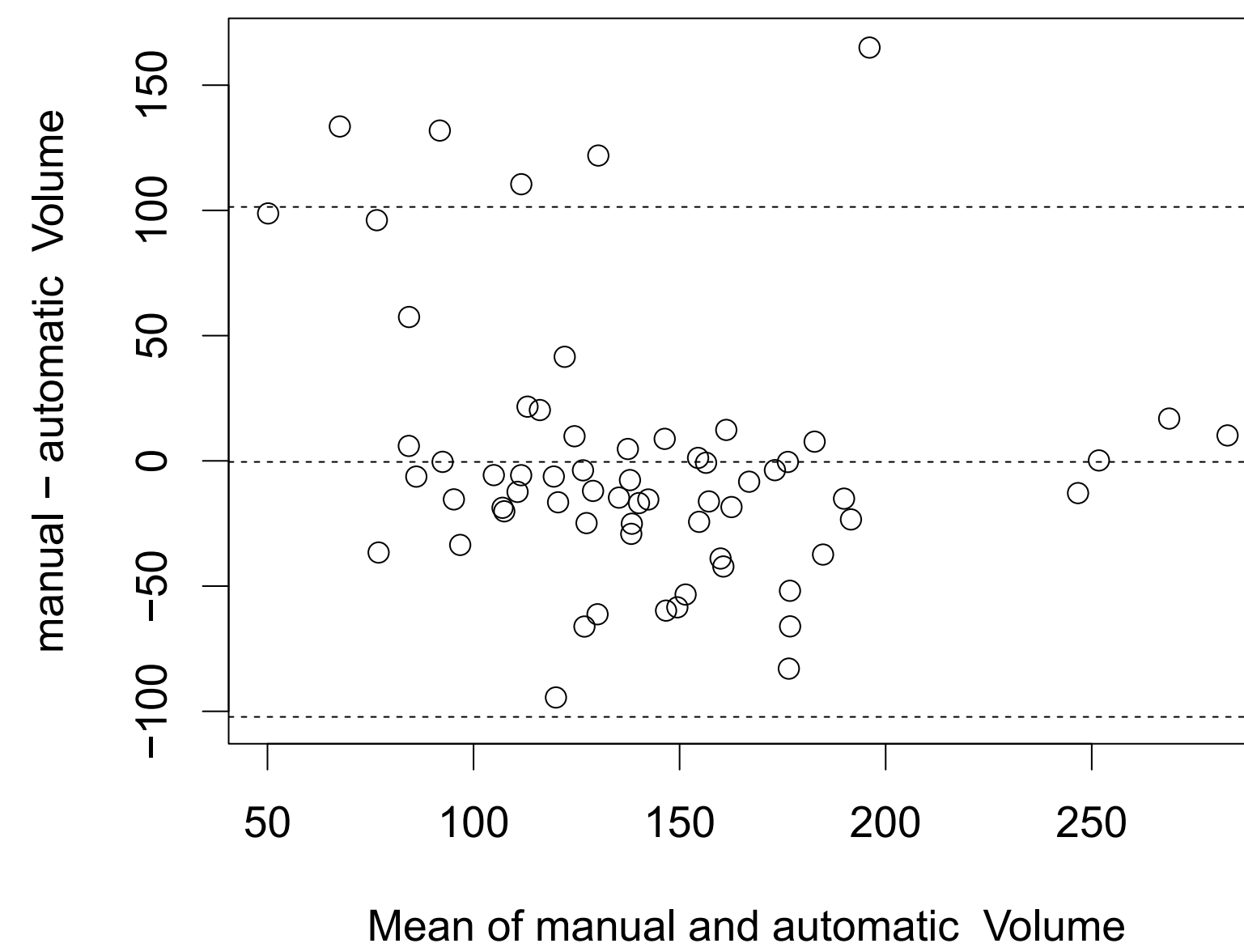**RLL B30f**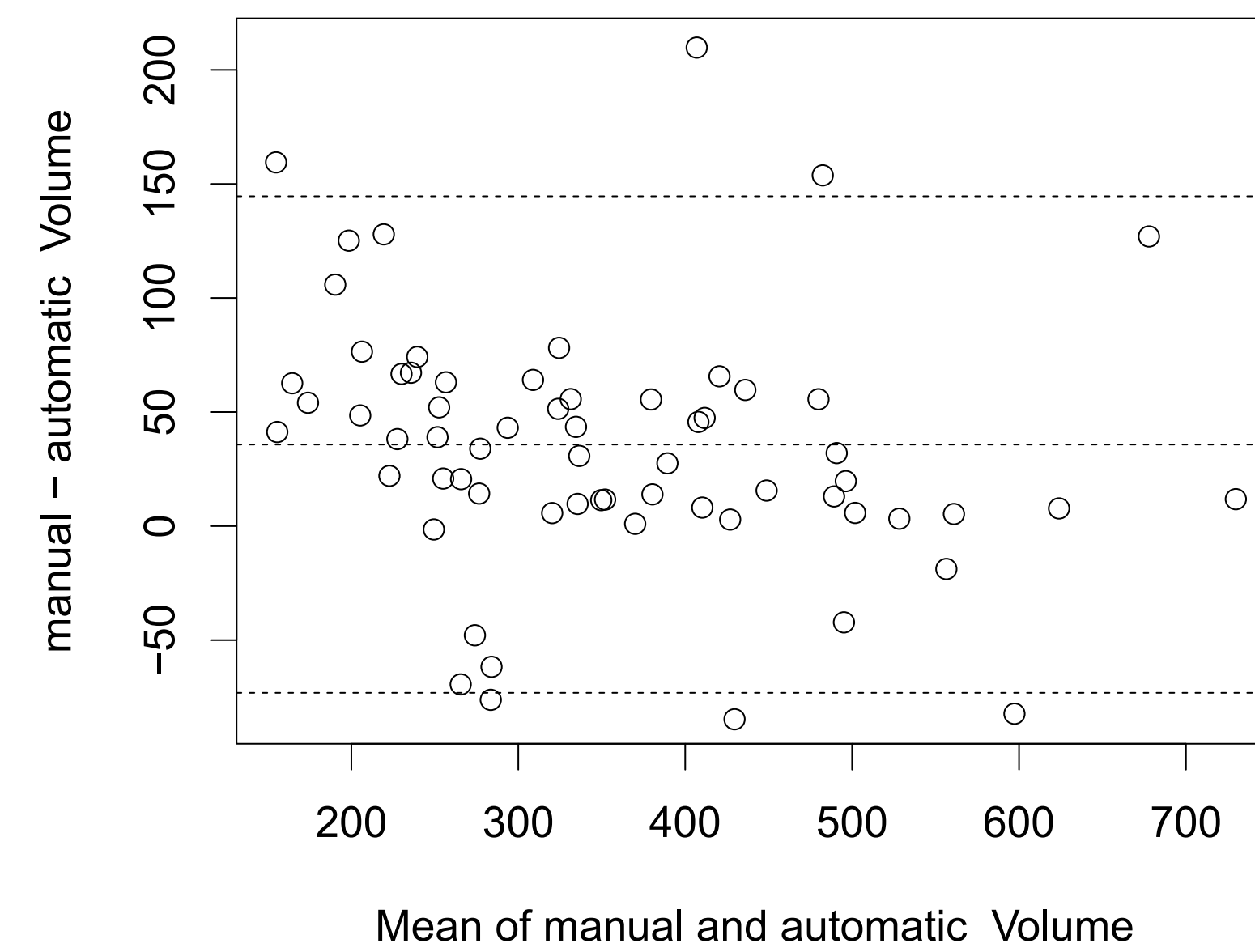**LUL B30f**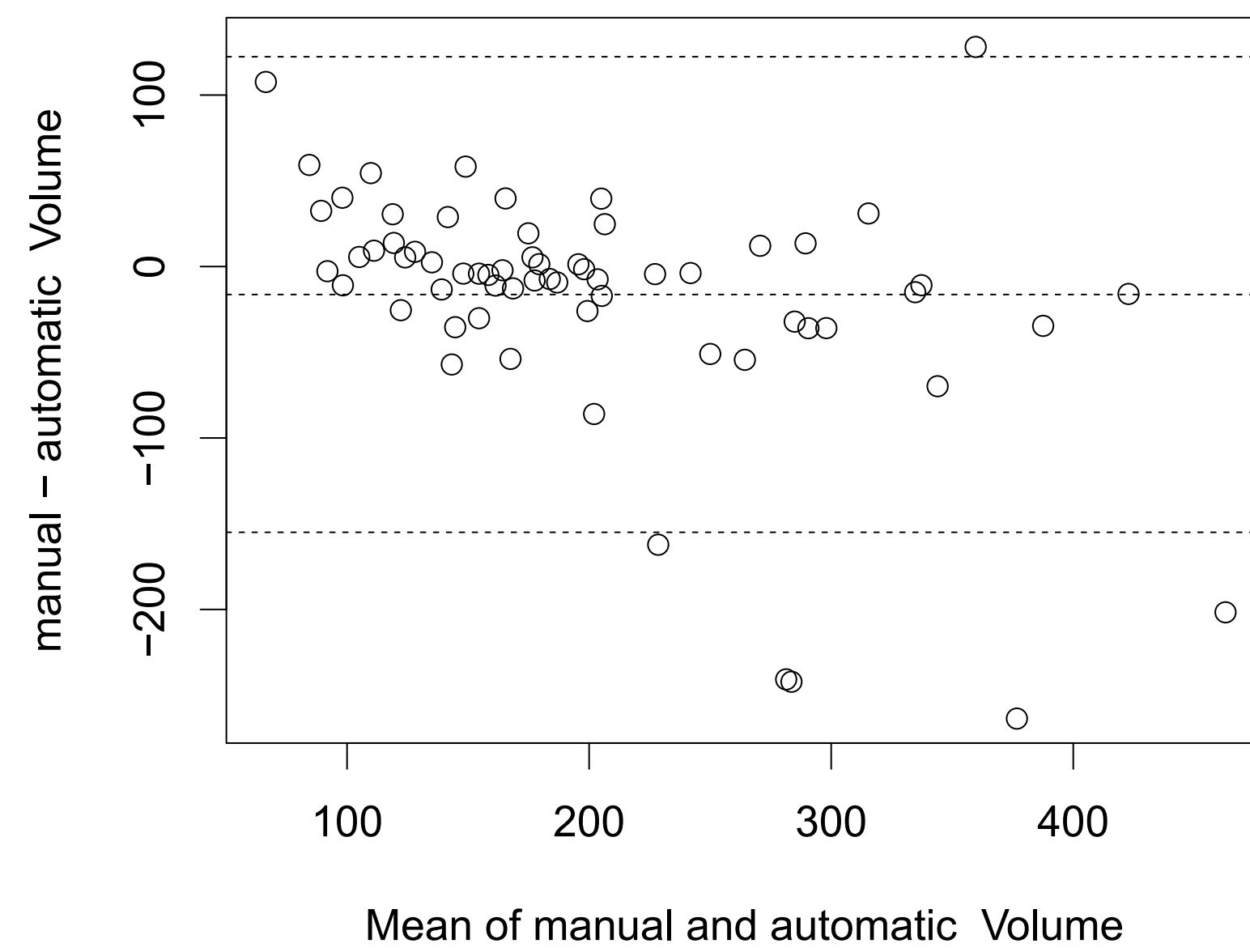**LLi B30f**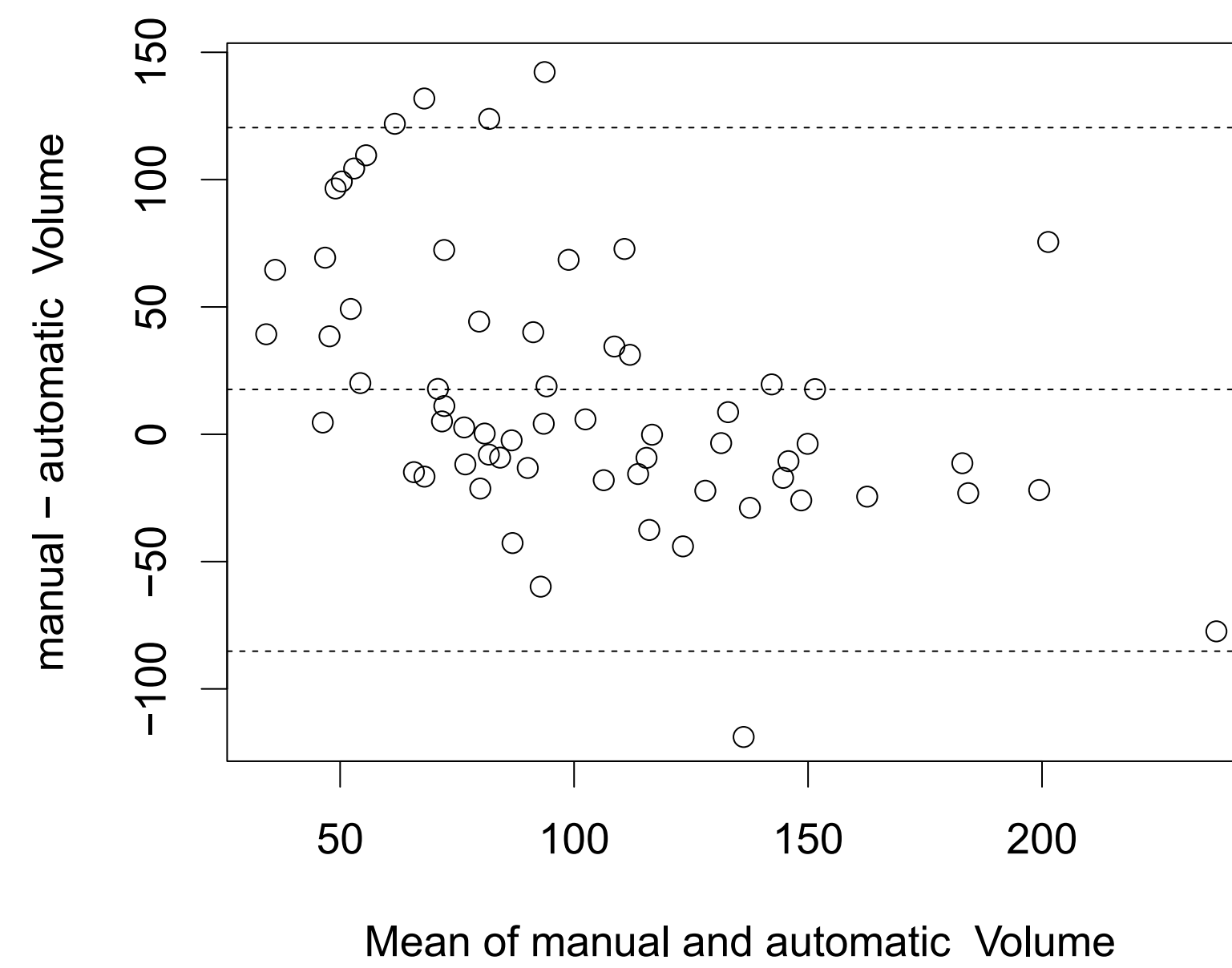**LLL B30f**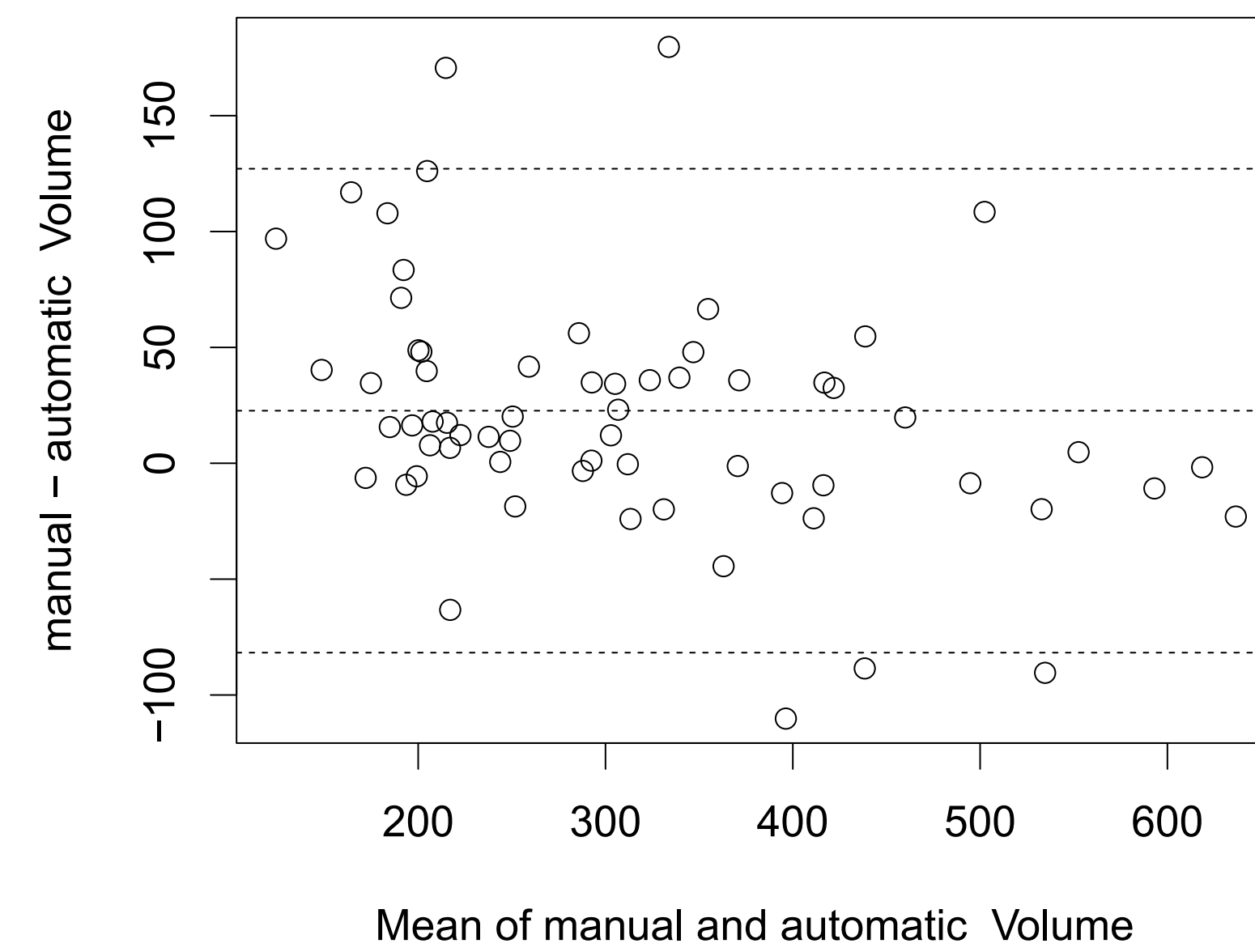**LUL+LLi B30f**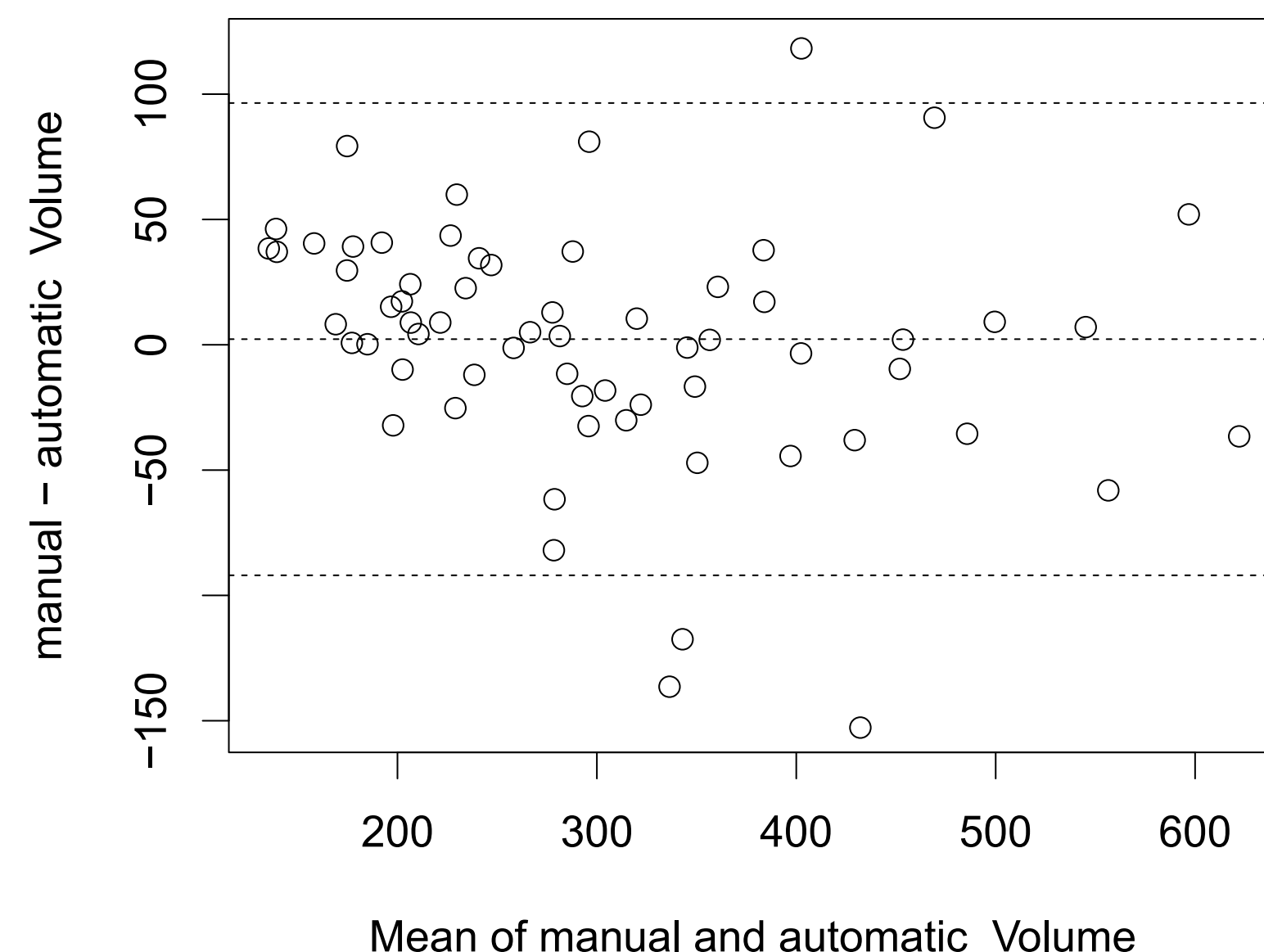

Supplement: S6 Fig — (PDF) [file pone.0194557.s006.pdf]

**RUL B60f**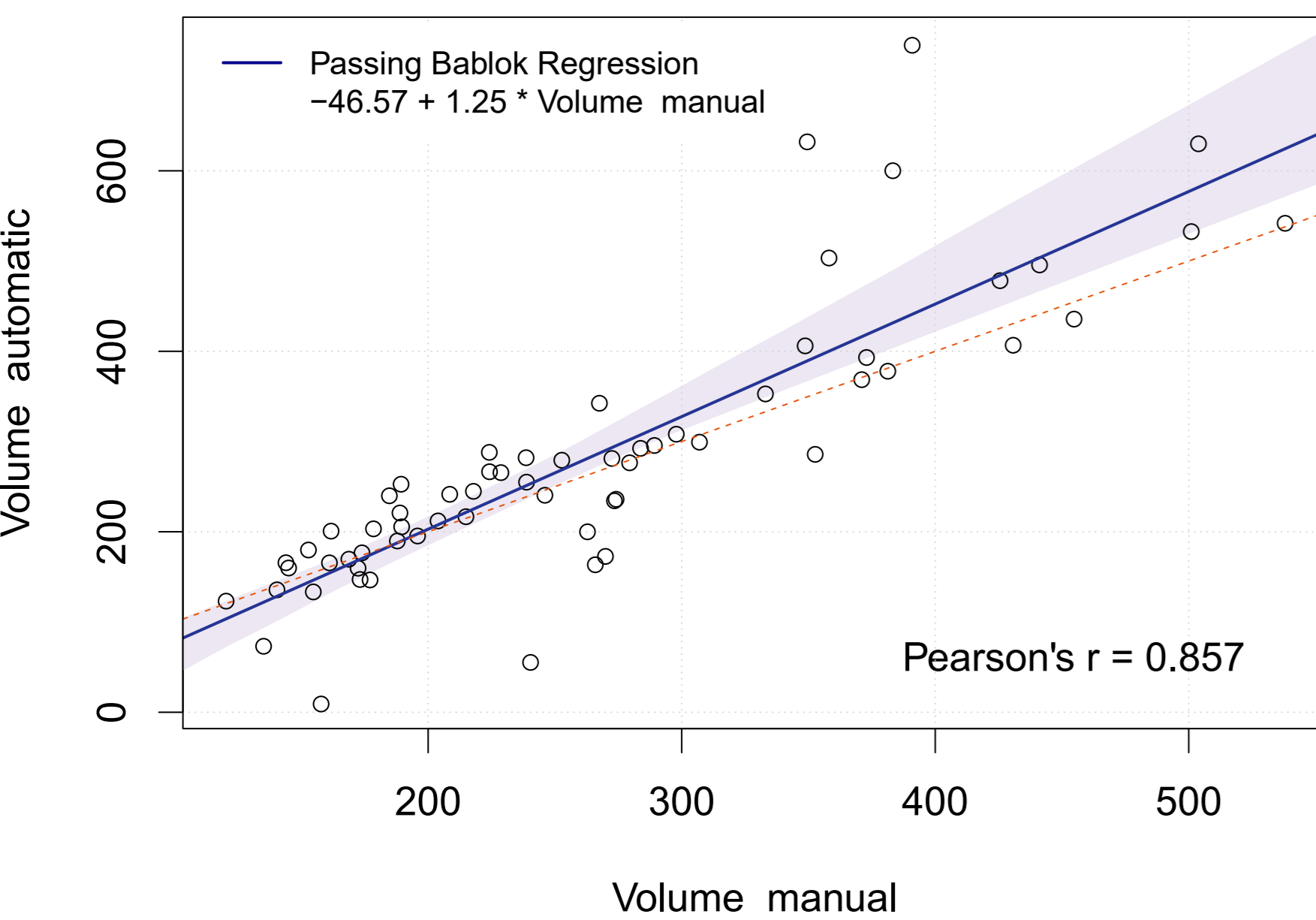**RML B60f**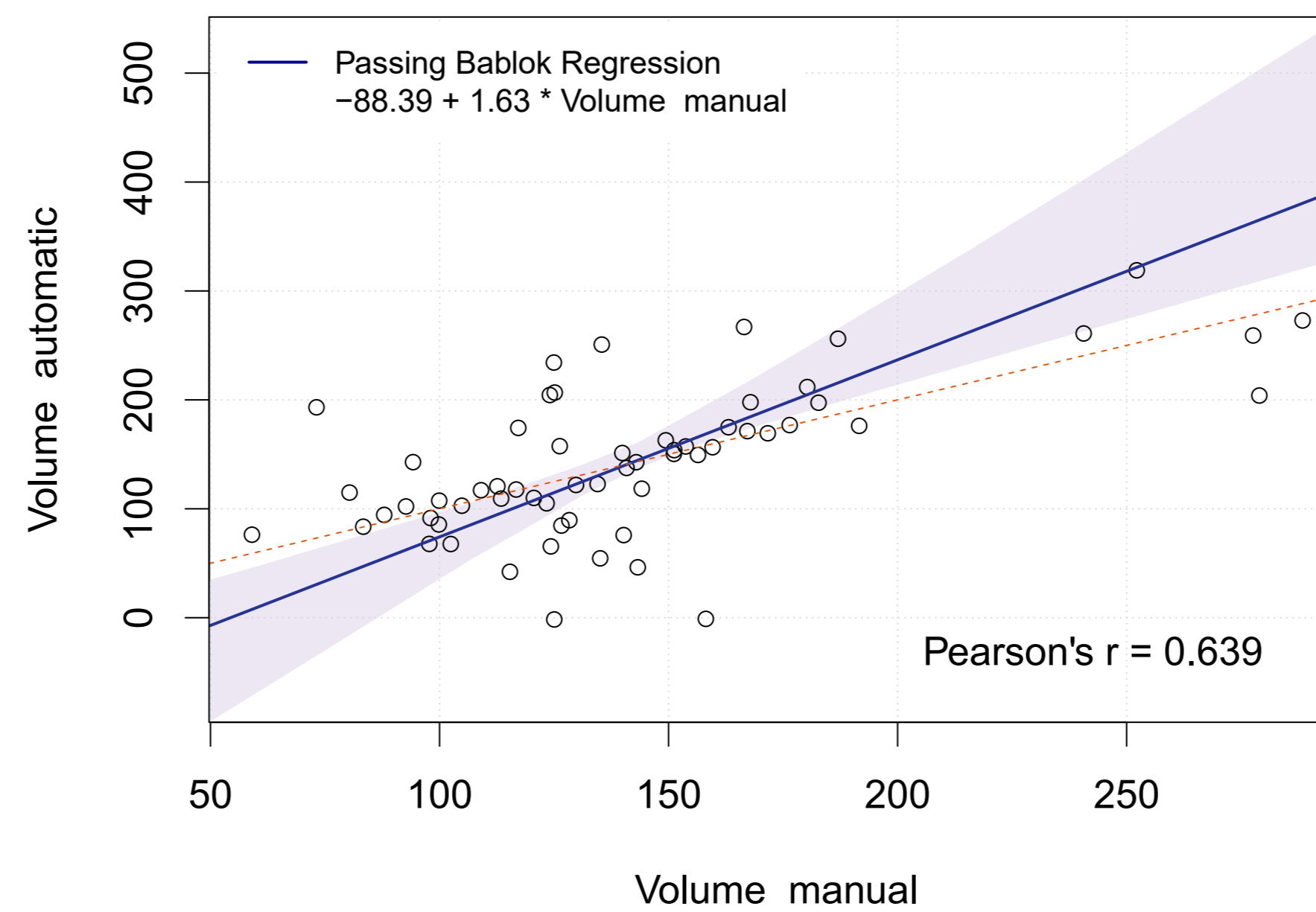**RLL B60f**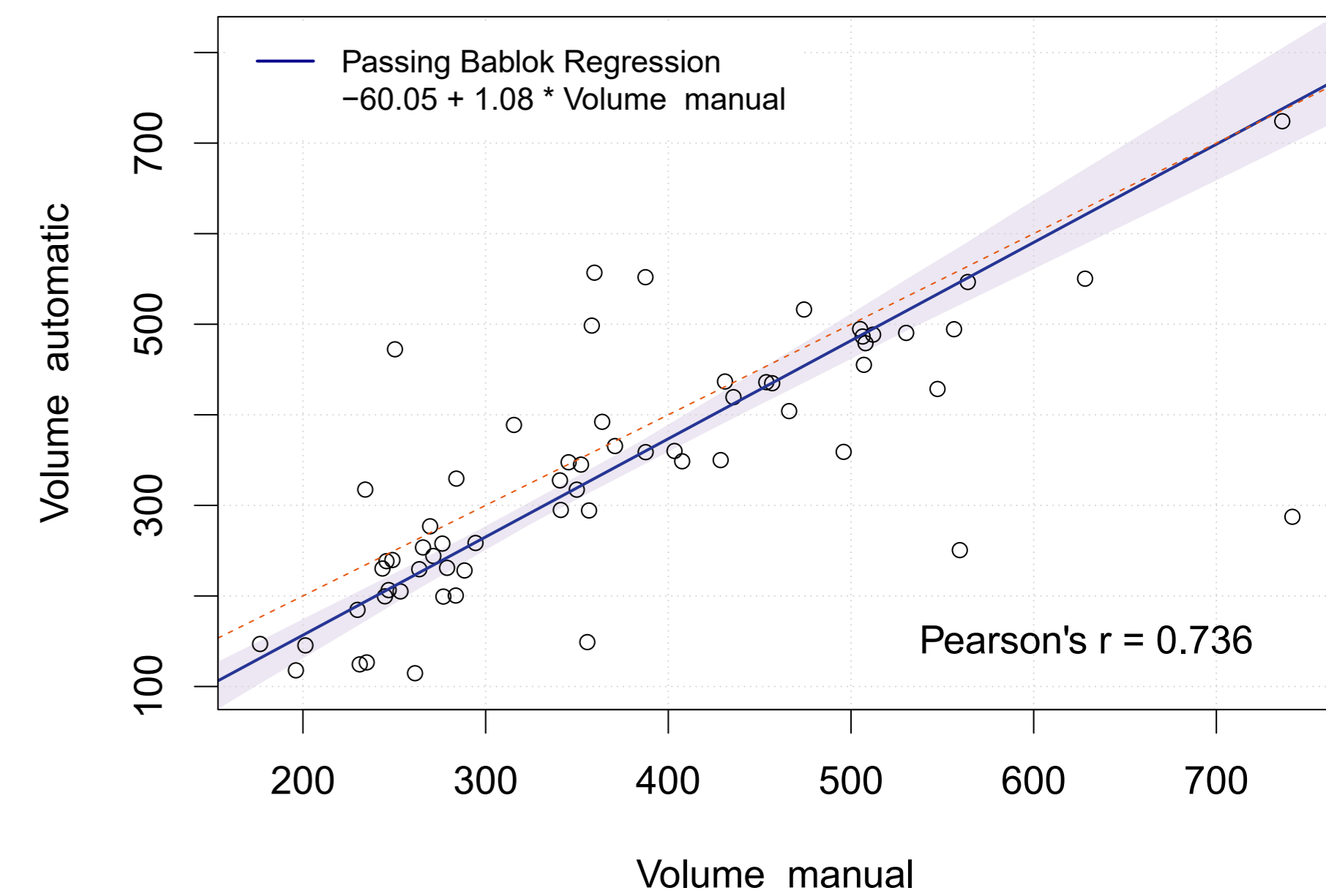**LUL B60f**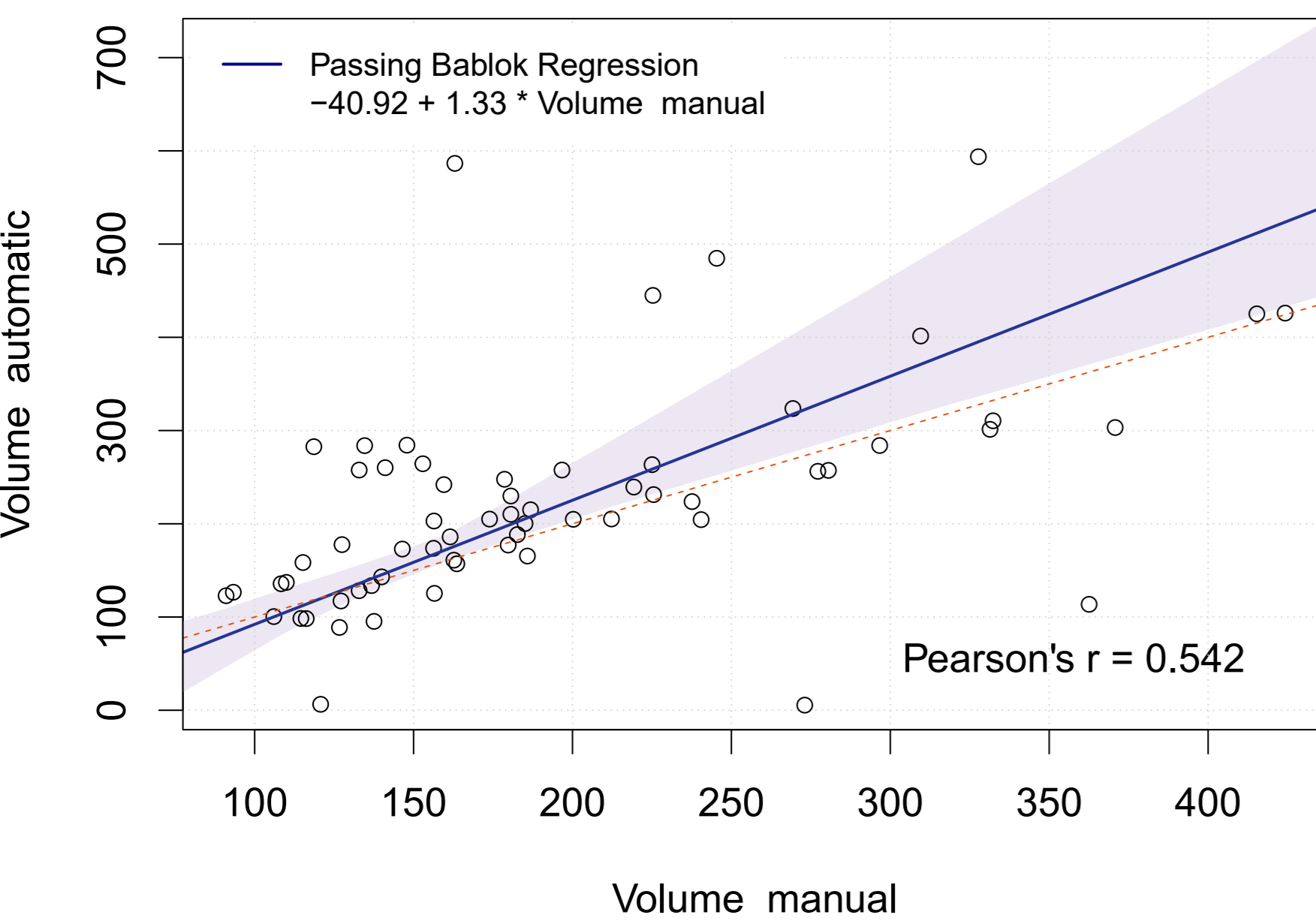**LLi B60f**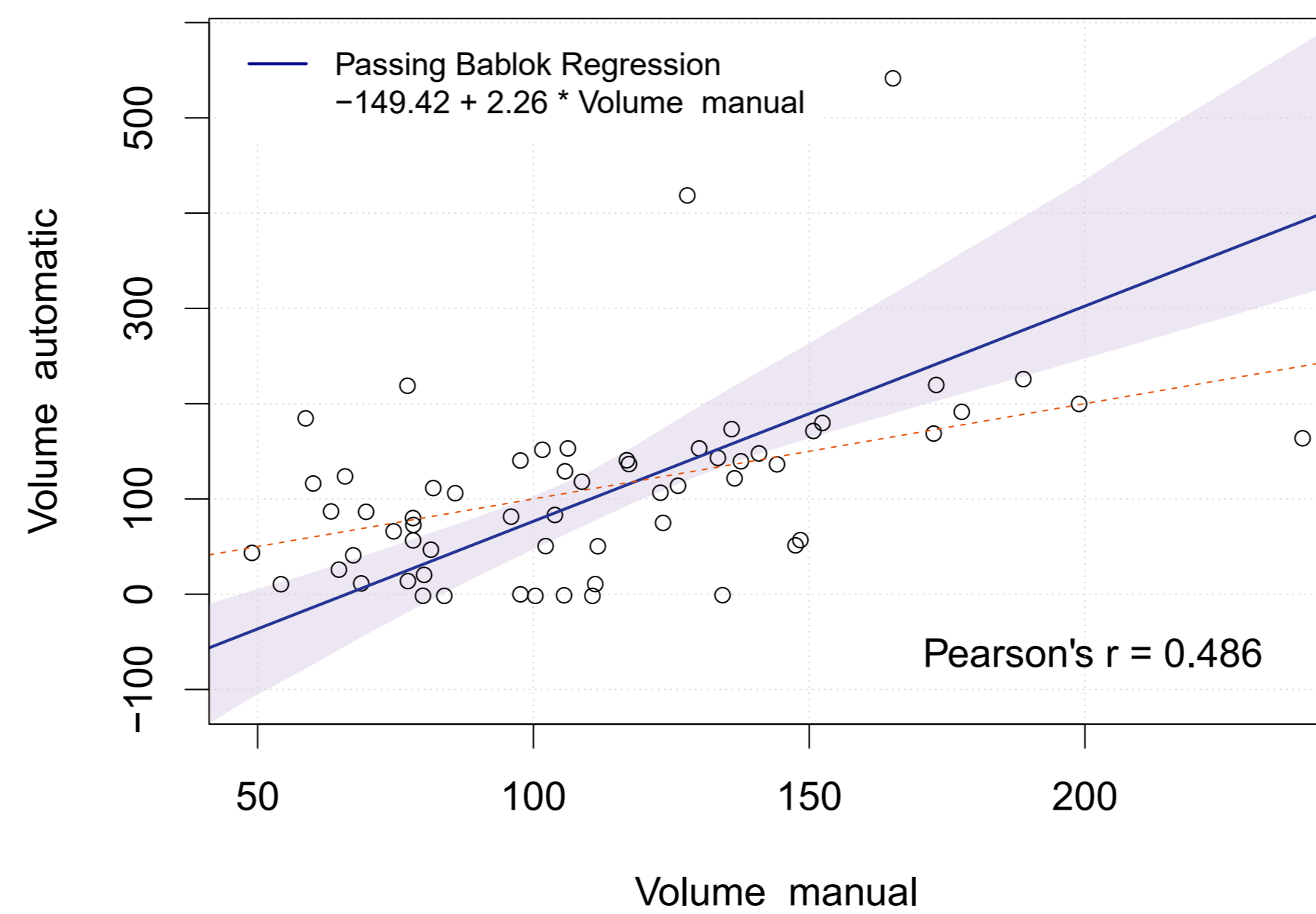**LLL B60f**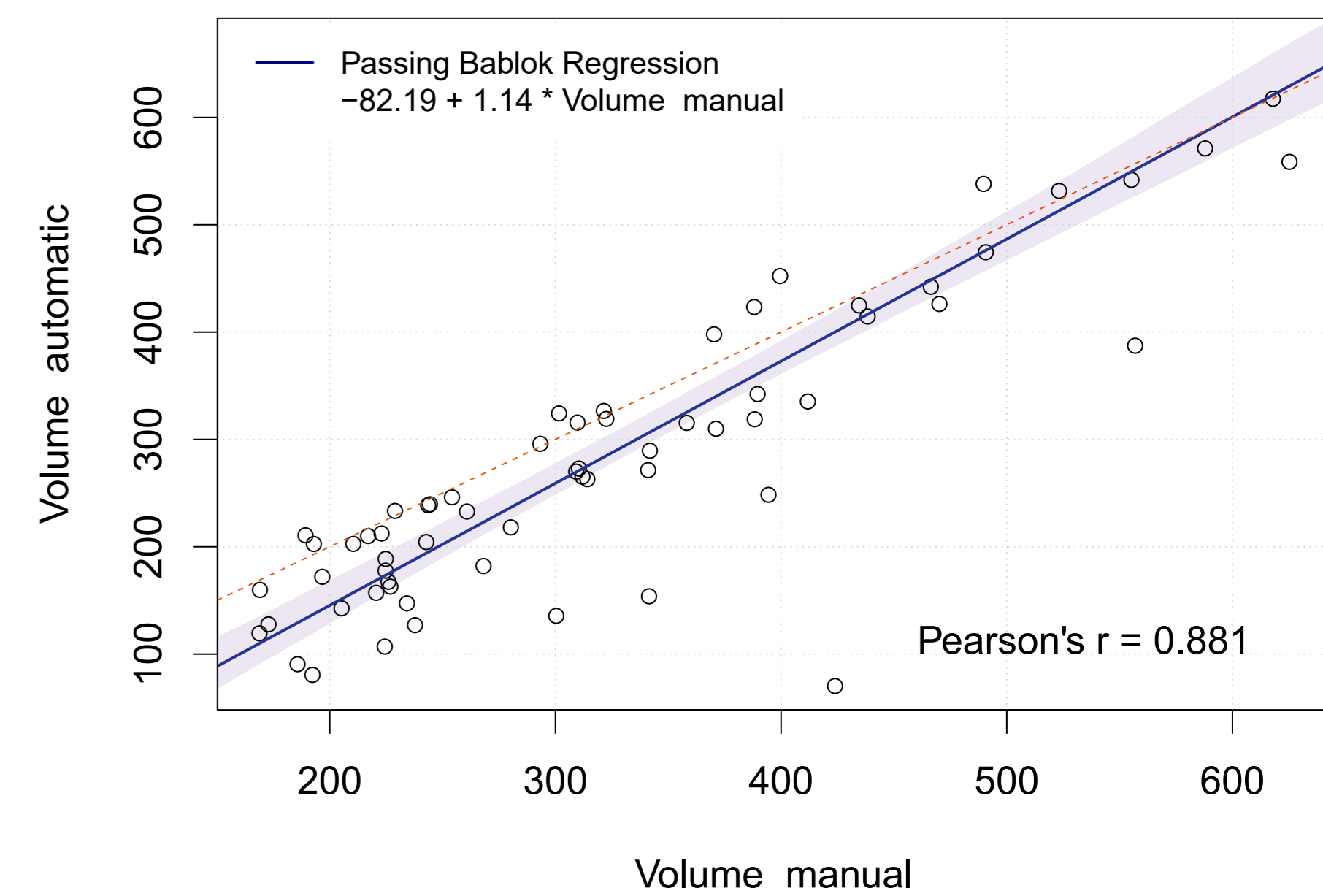**LUL+LLi B60f**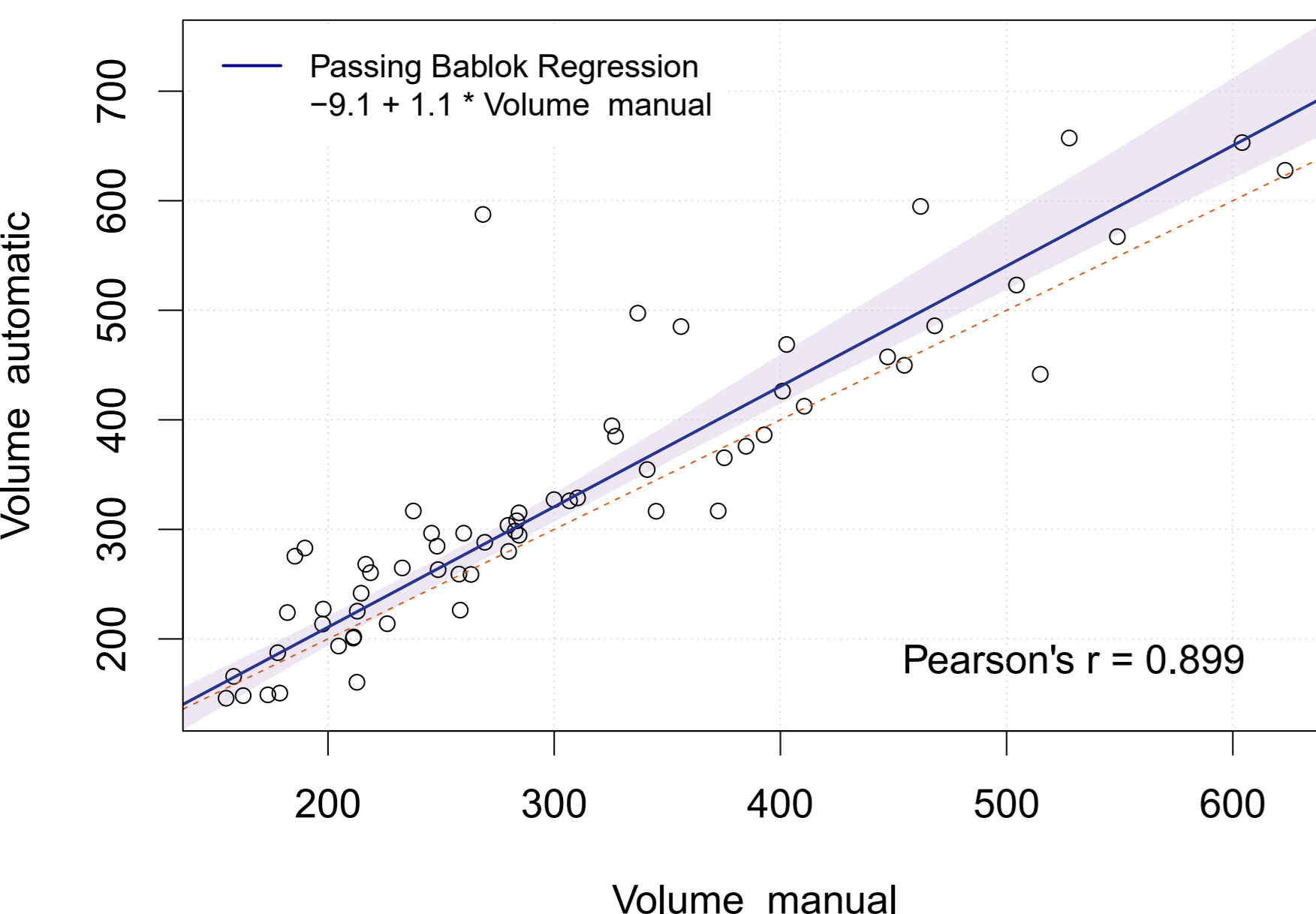

Supplement: S7 Fig — (PDF) [file pone.0194557.s007.pdf]

**RUL B60f**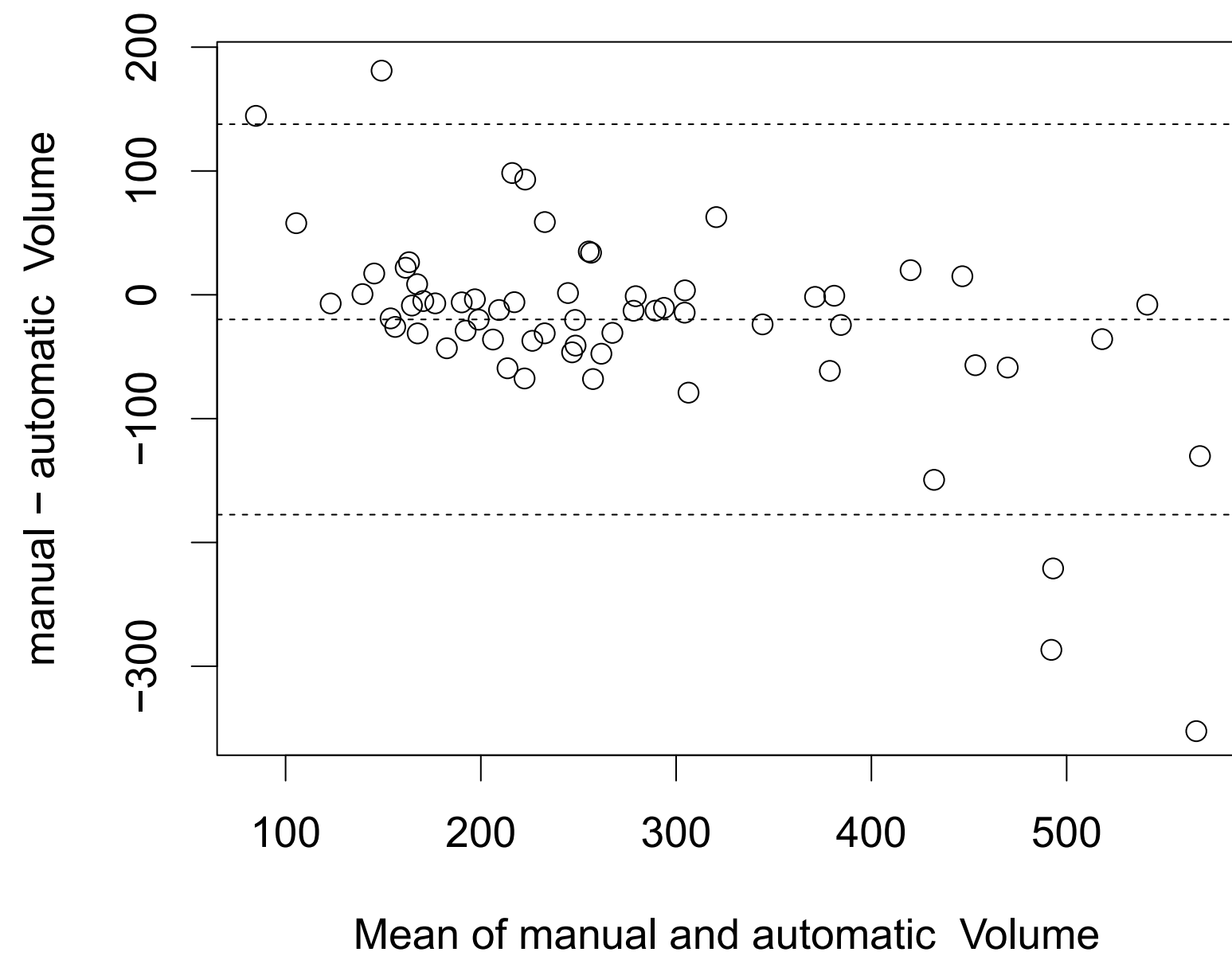**RML B60f**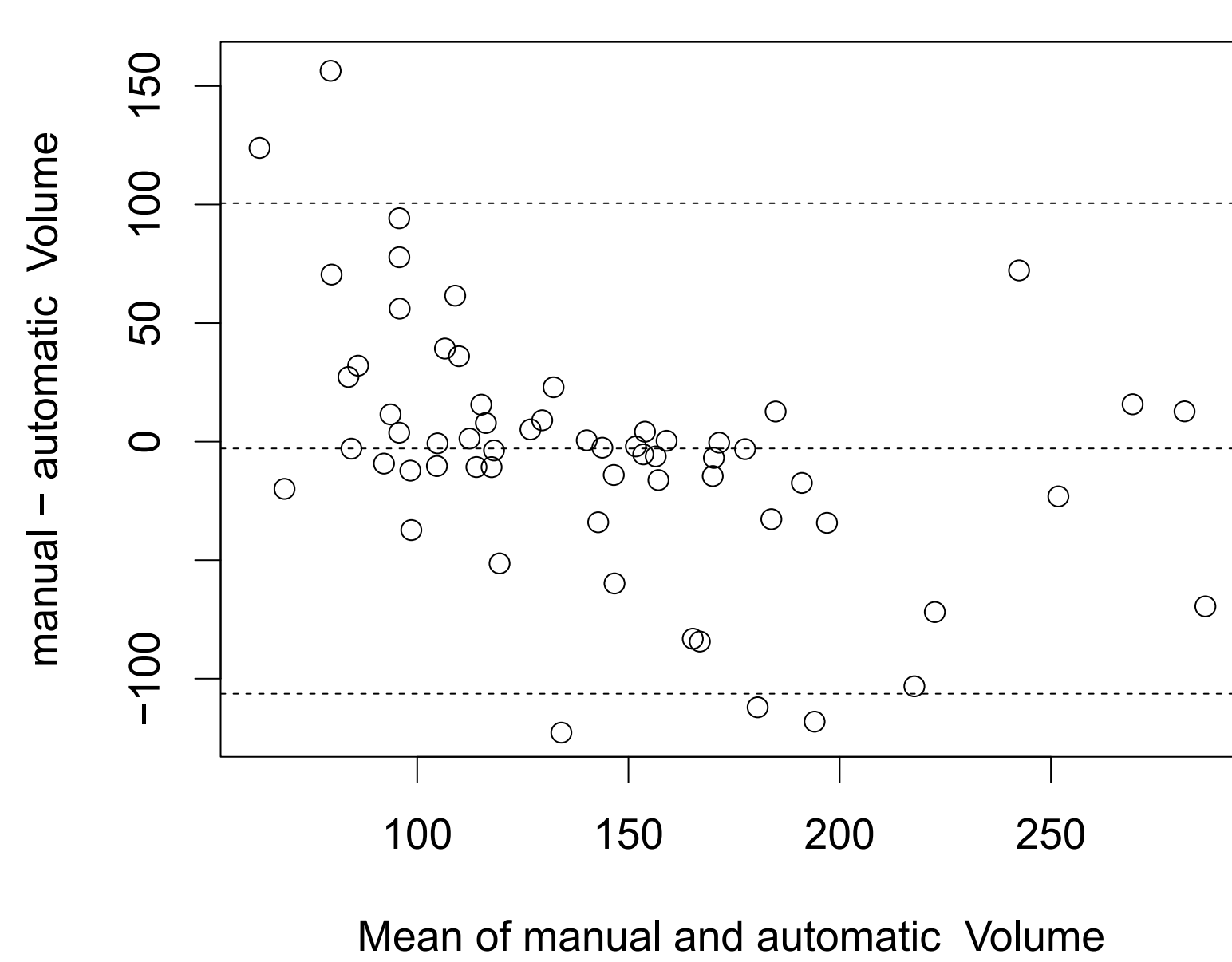**RLL B60f**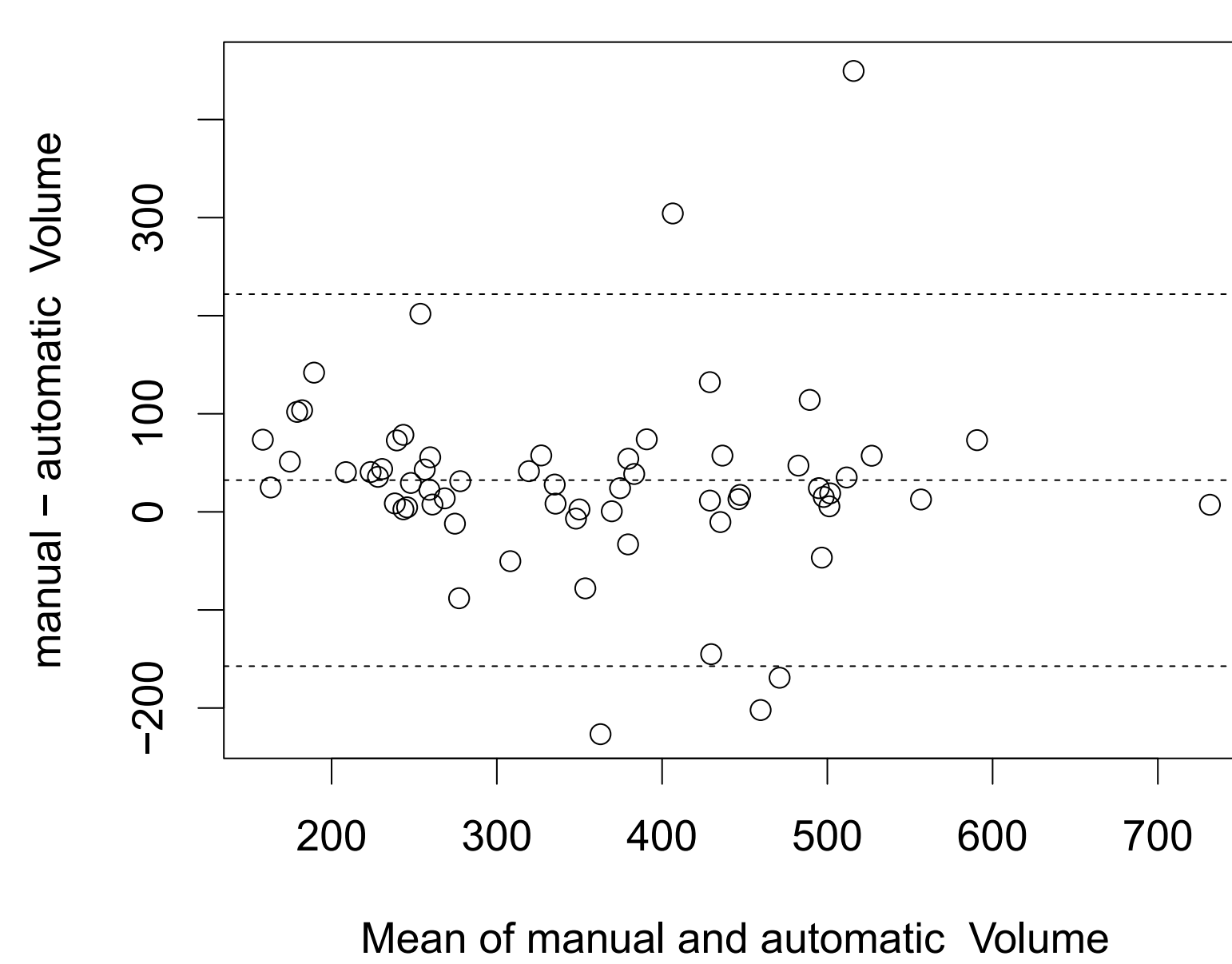**LUL B60f**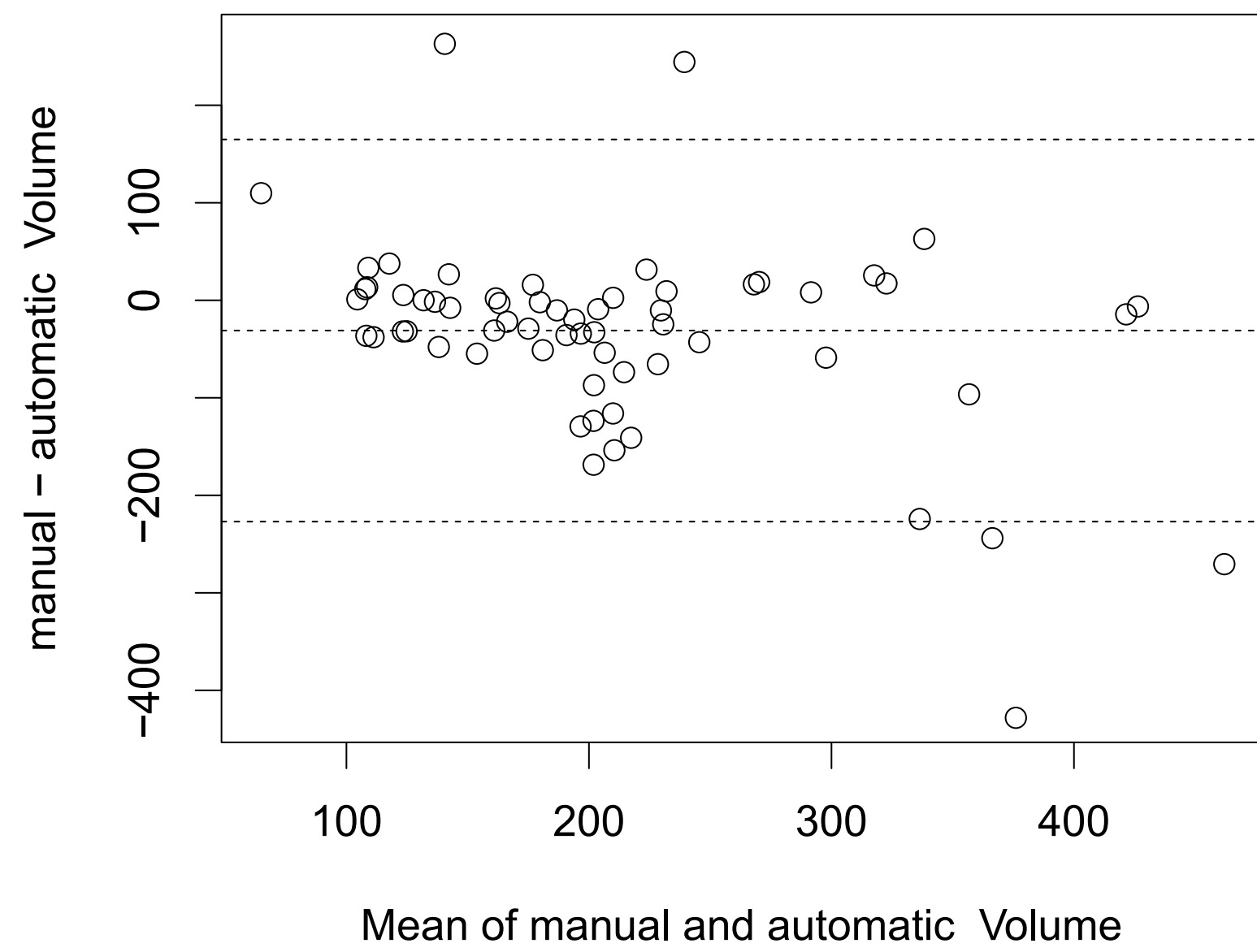**LLi B60f**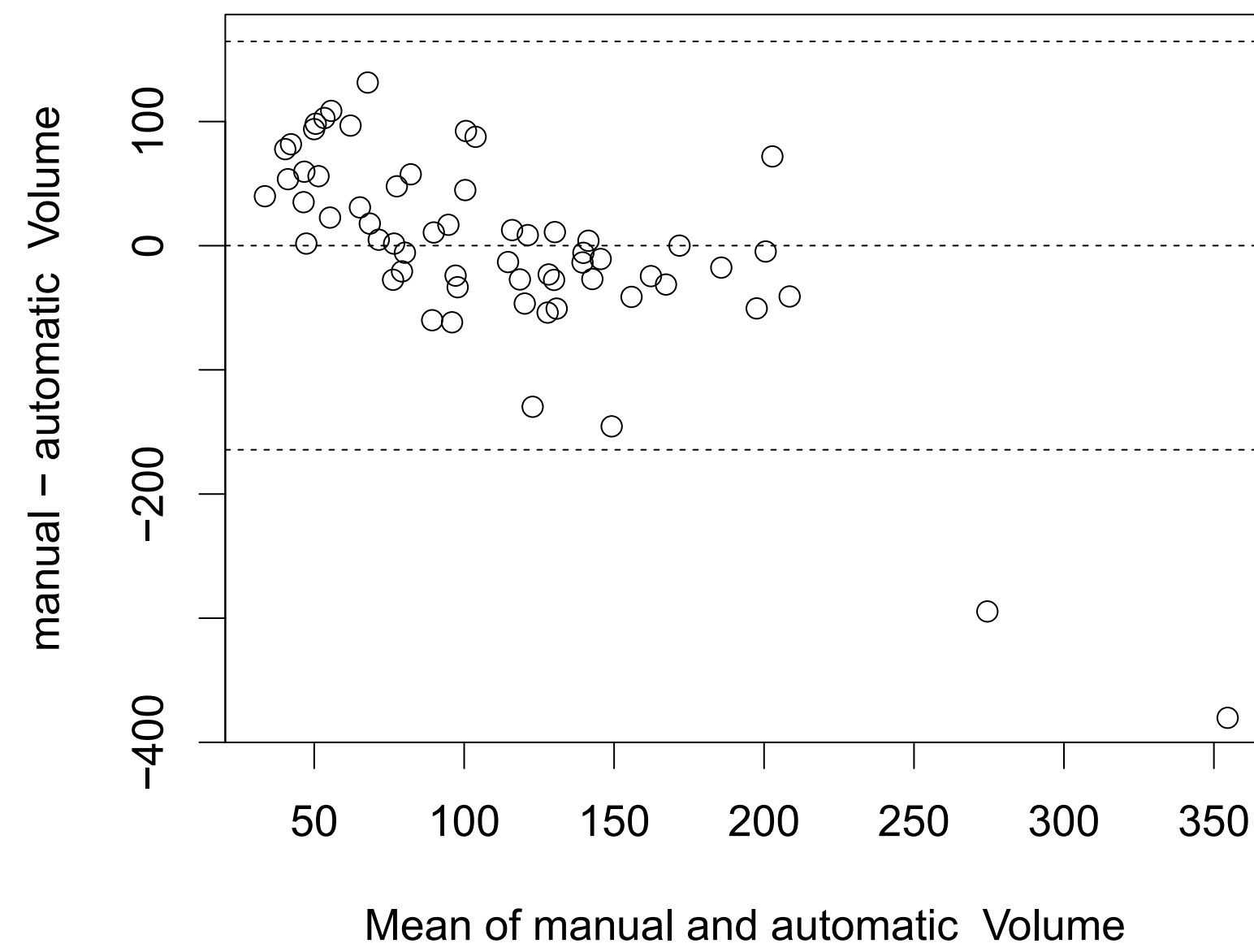**LLL B60f**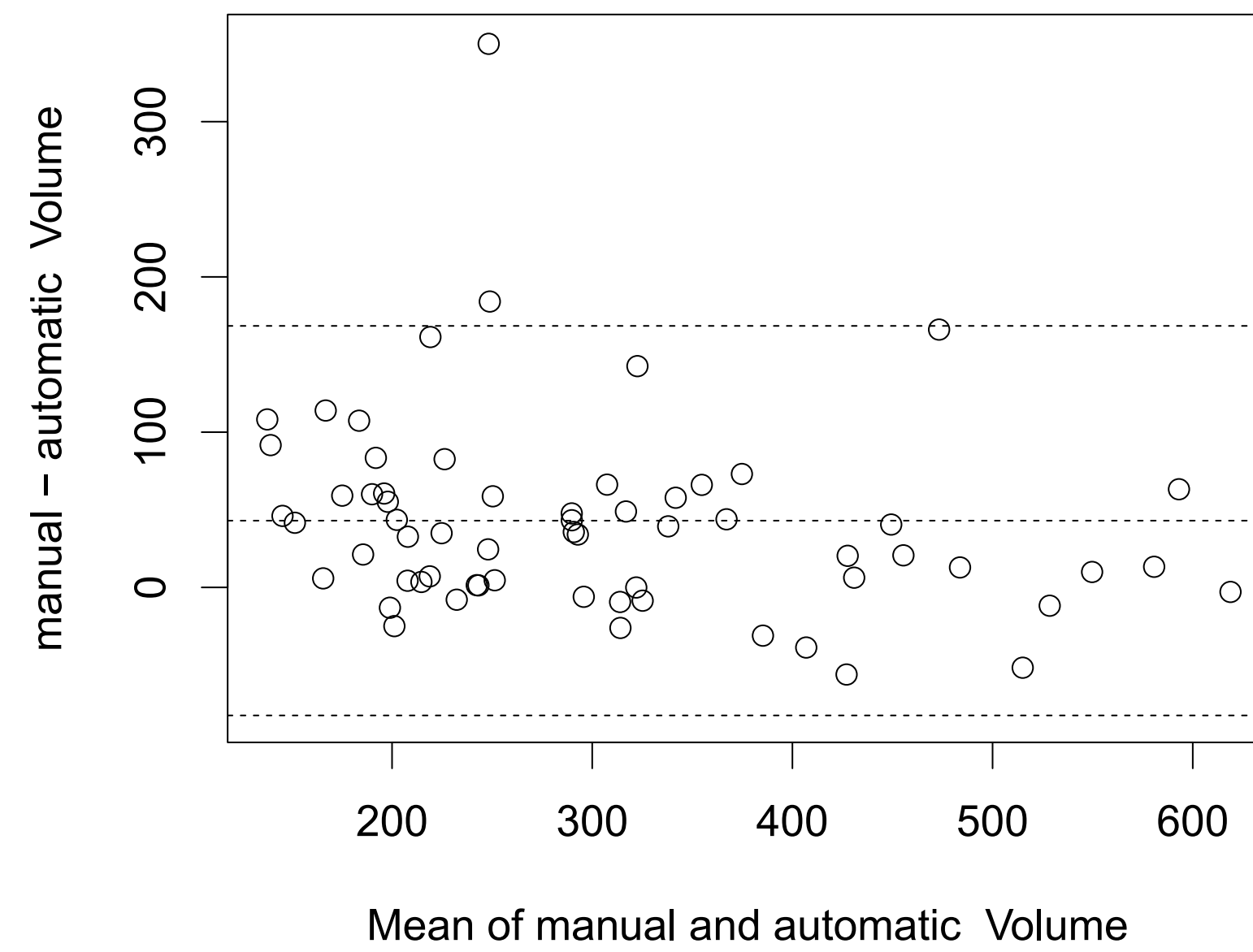**LUL+LLi B60f**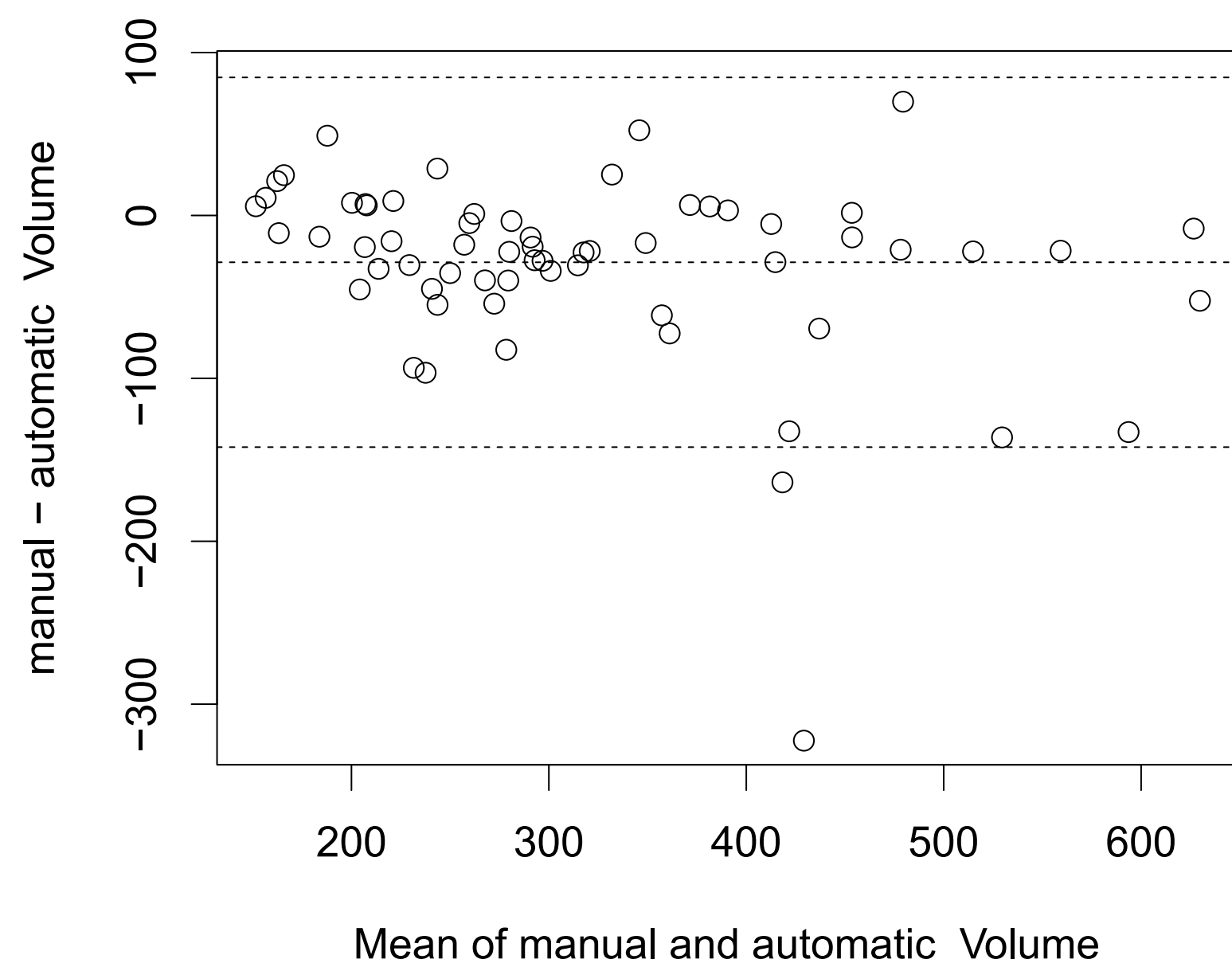

Supplement: S8 Fig — (PDF) [file pone.0194557.s008.pdf]

**RUL B30f**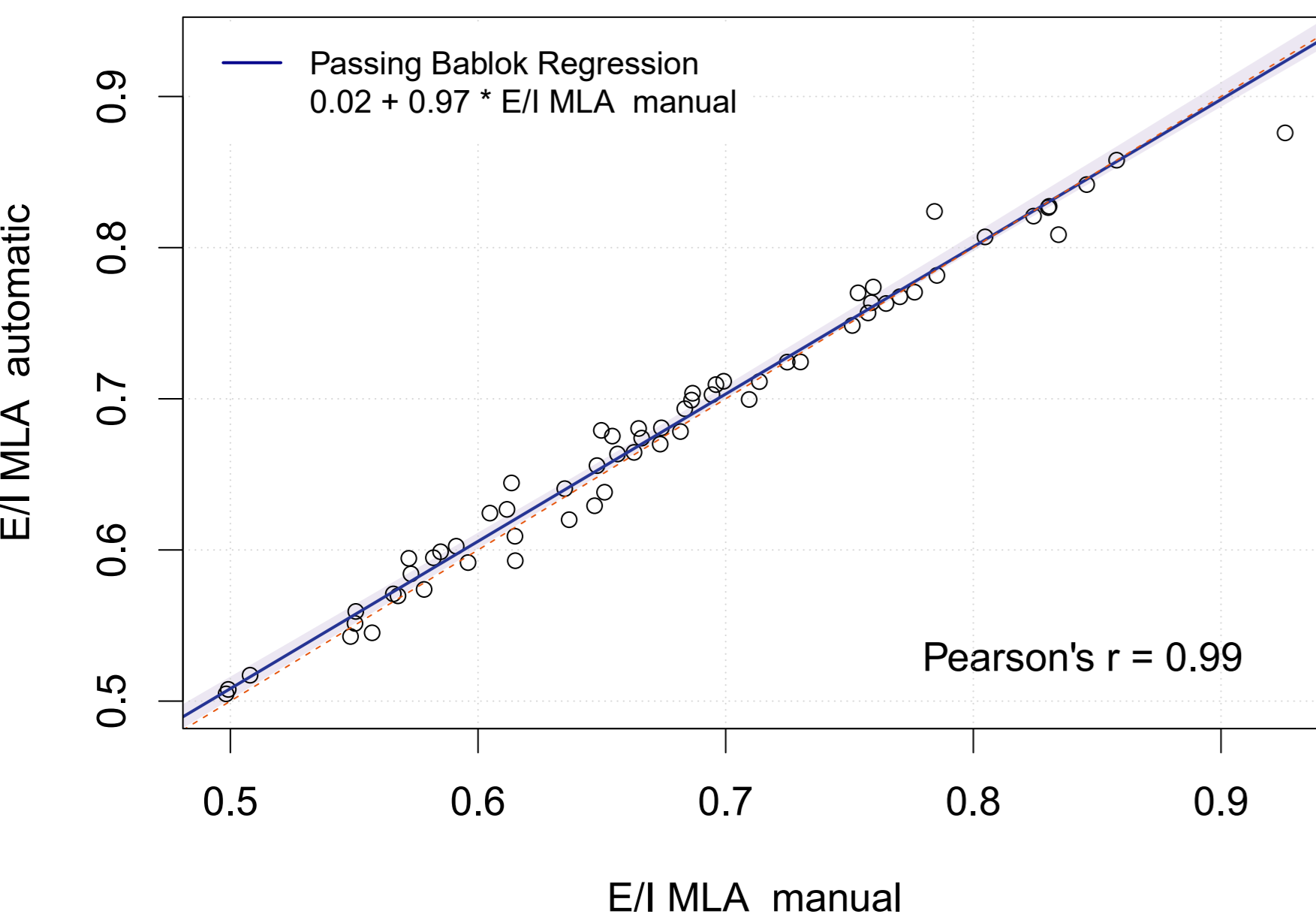**RML B30f**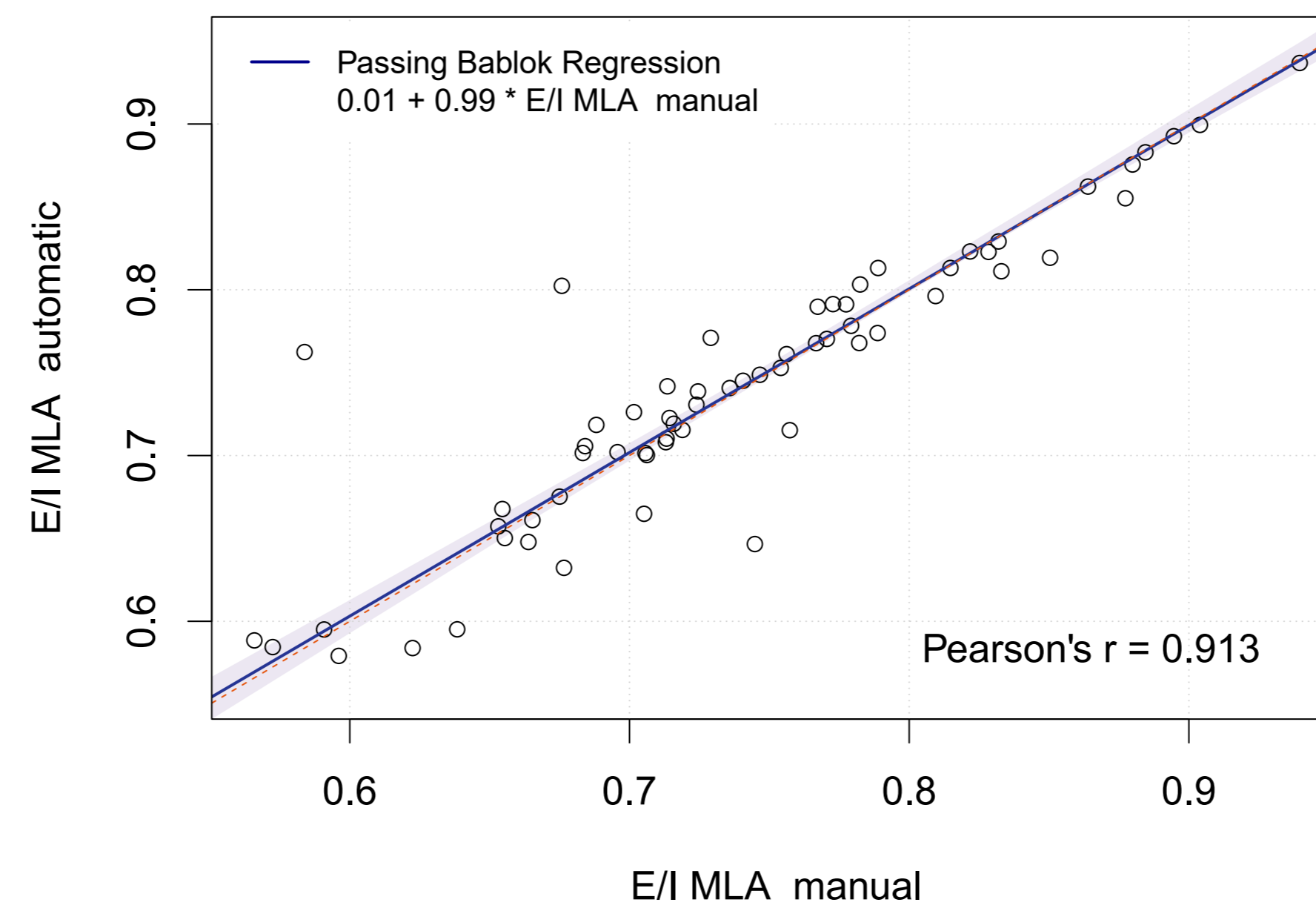**RLL B30f**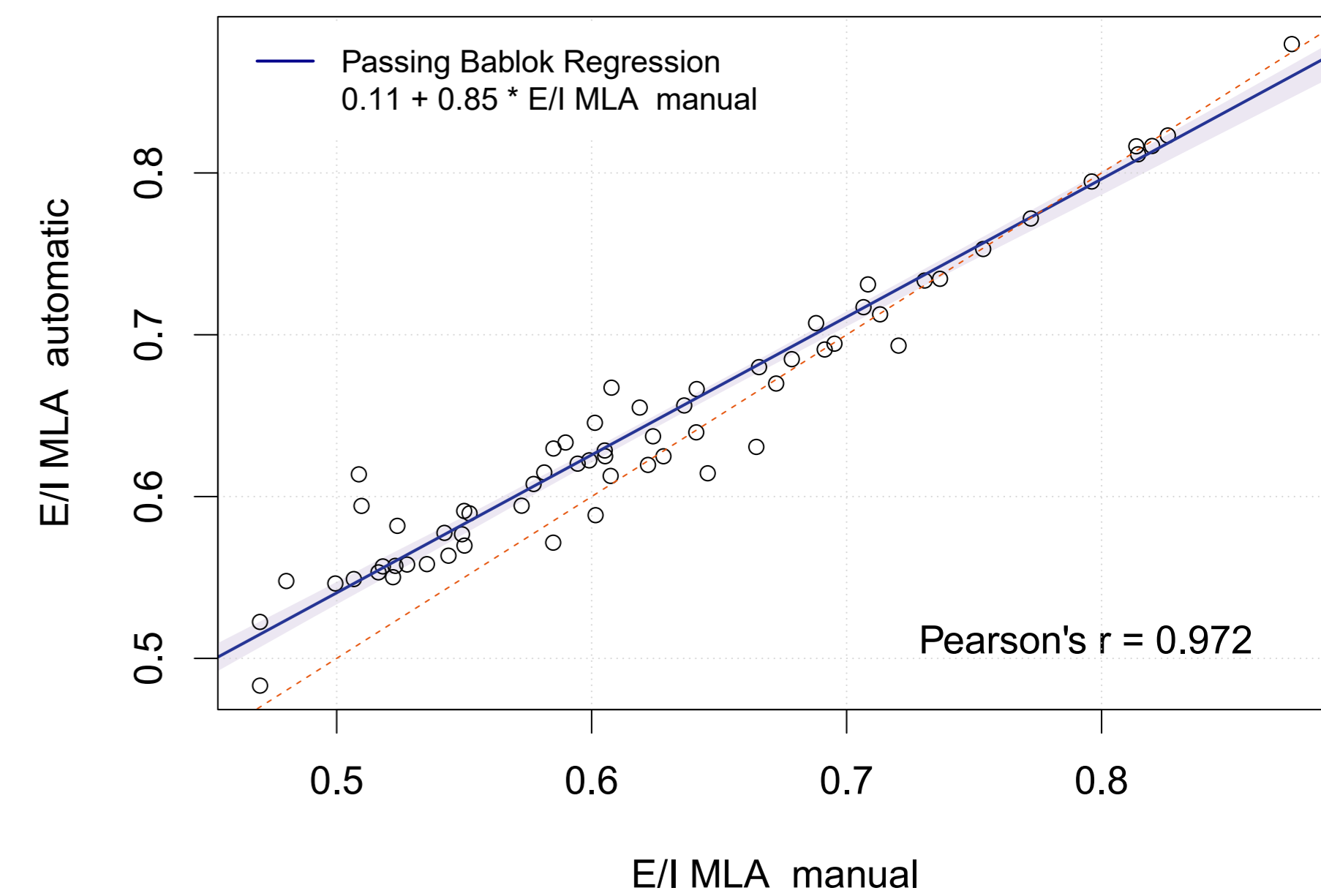**LUL B30f**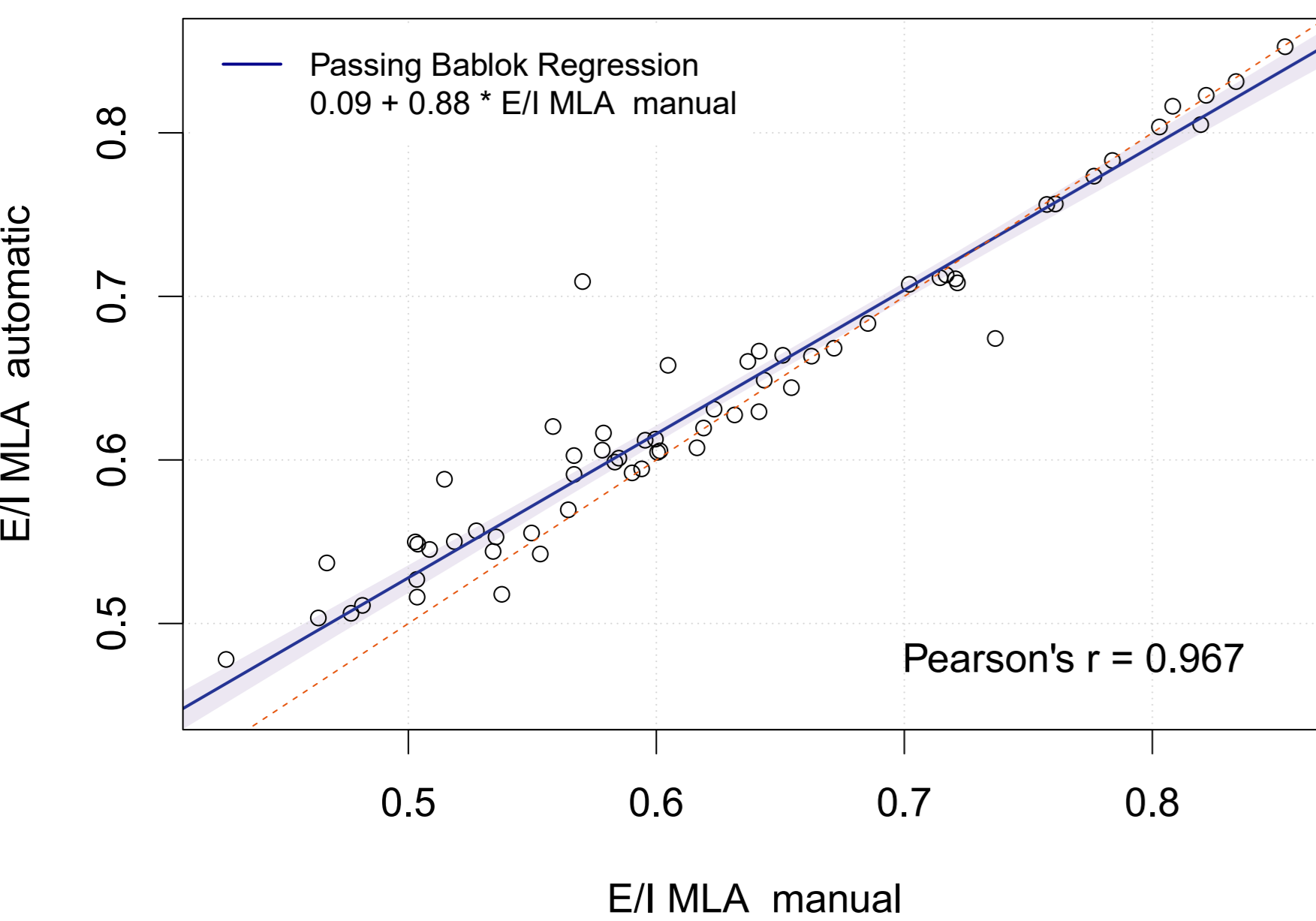**LLi B30f**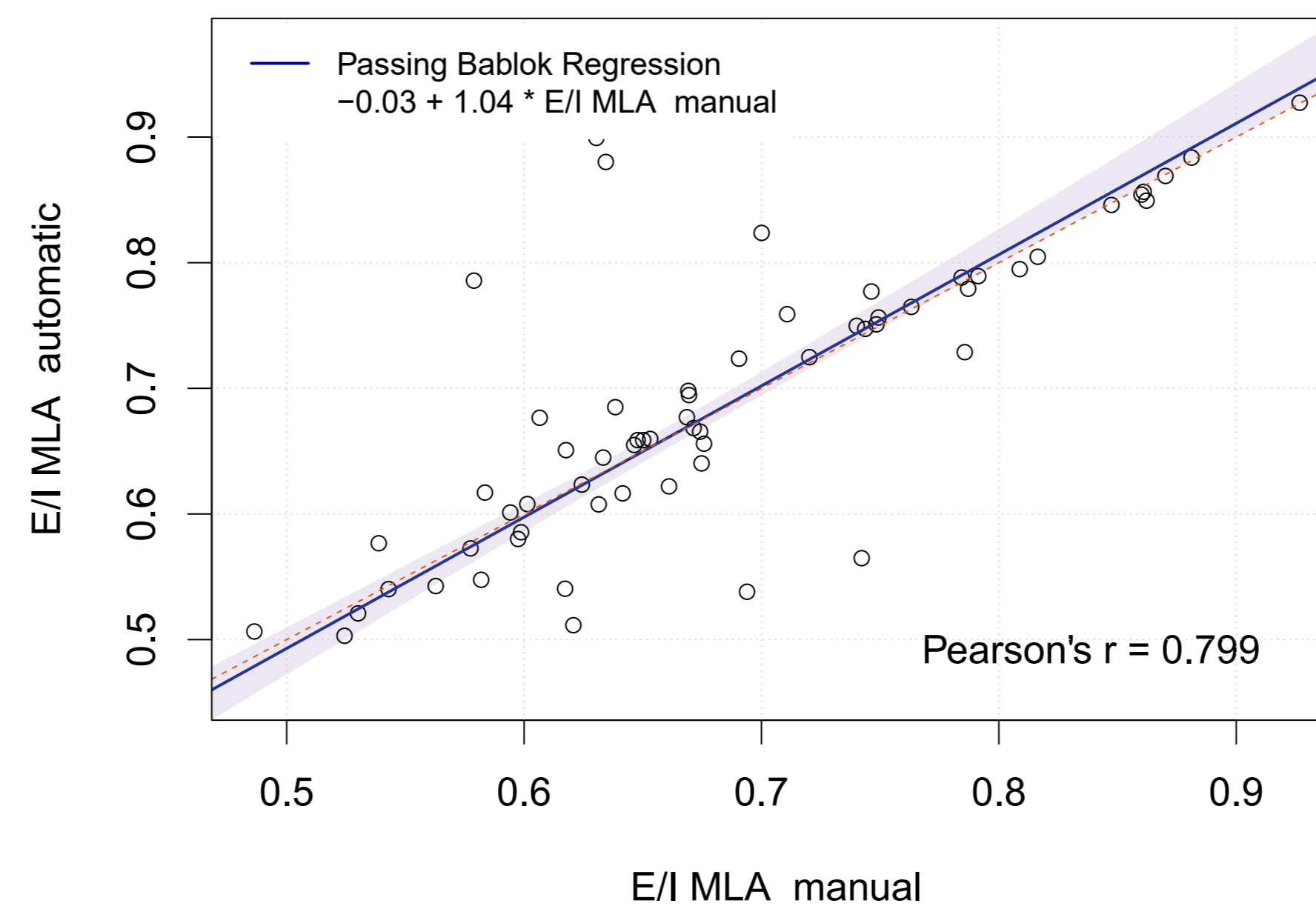**LLL B30f**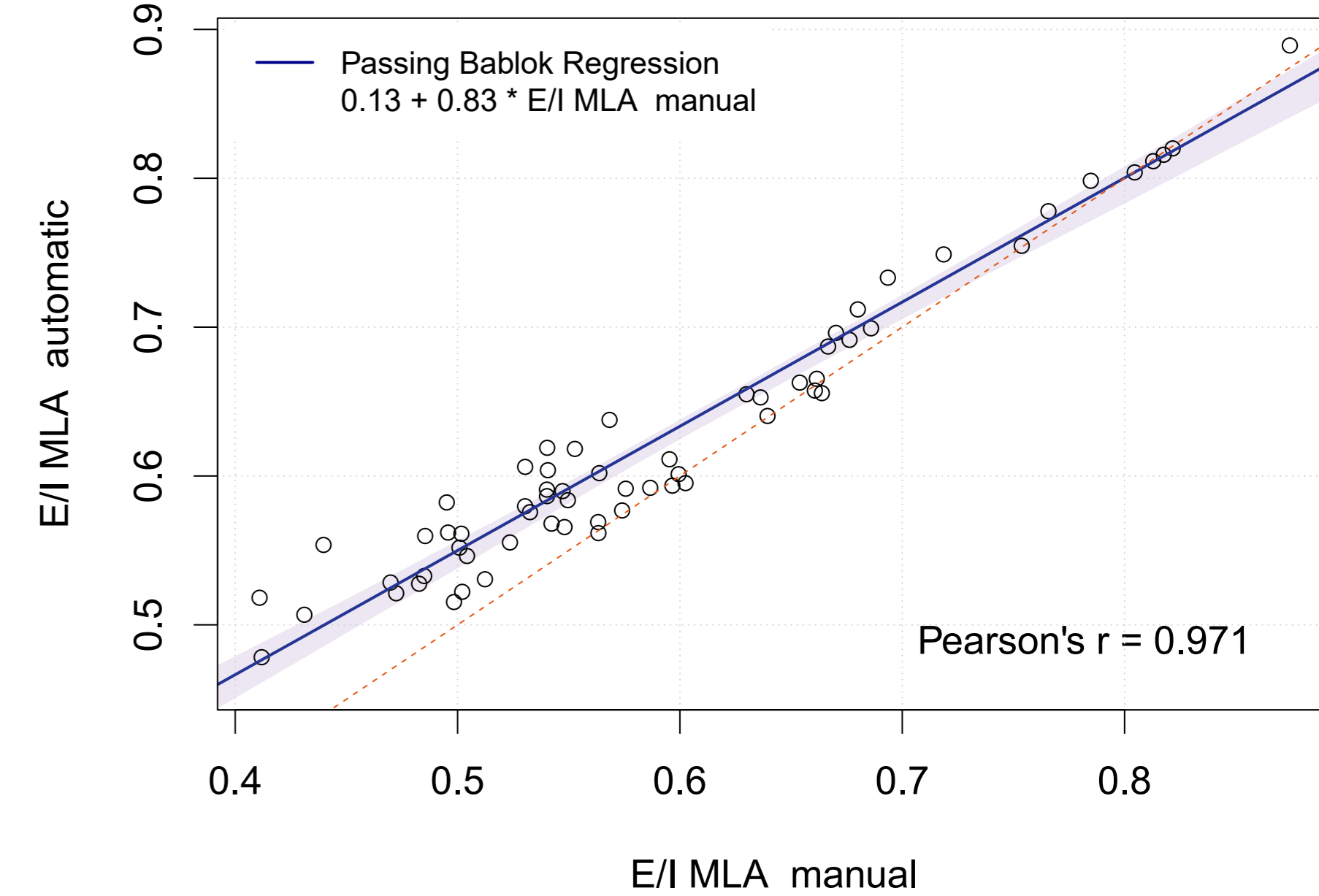**LUL+LLi B30f**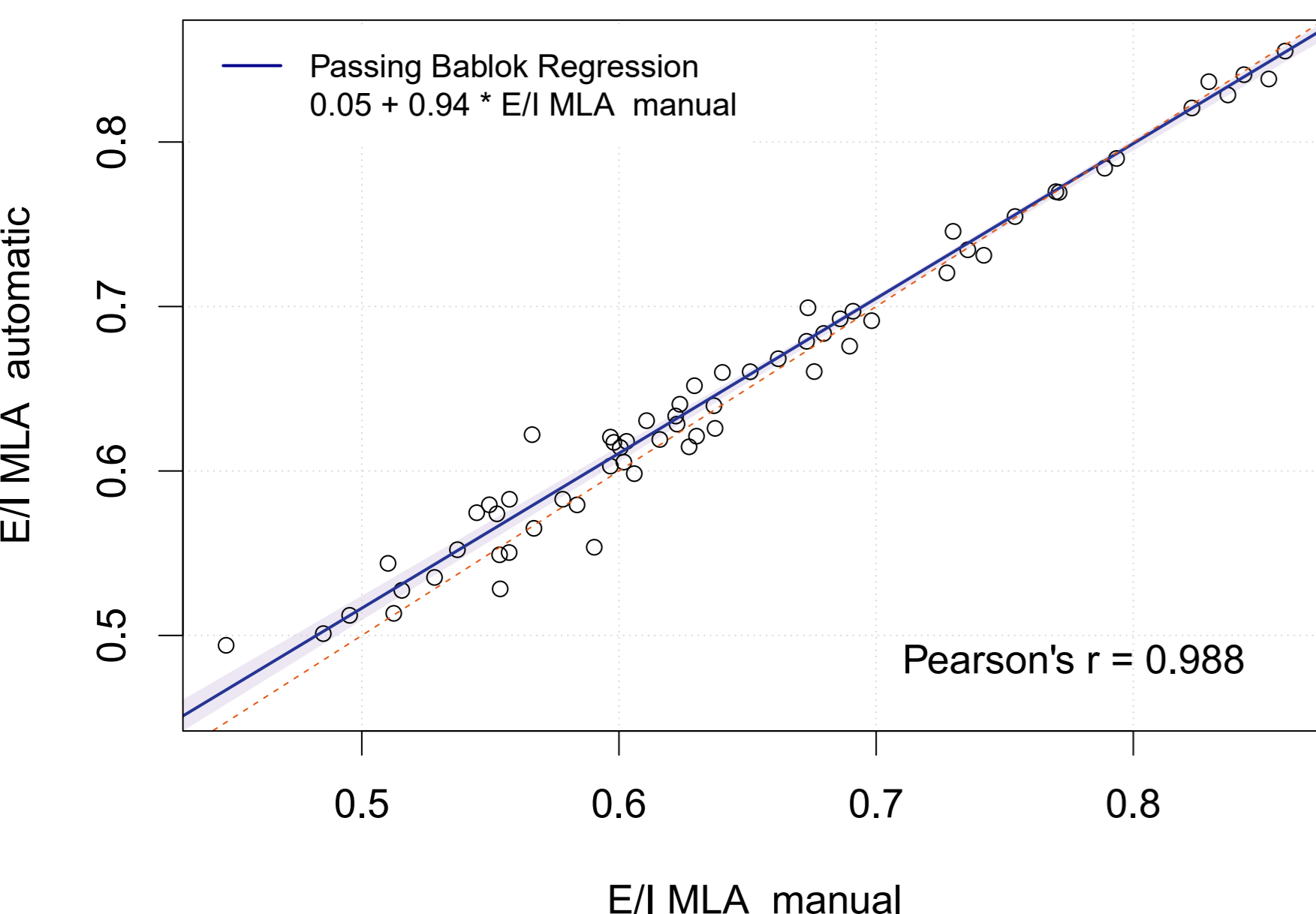

Supplement: S9 Fig — (PDF) [file pone.0194557.s009.pdf]

**RUL B30f**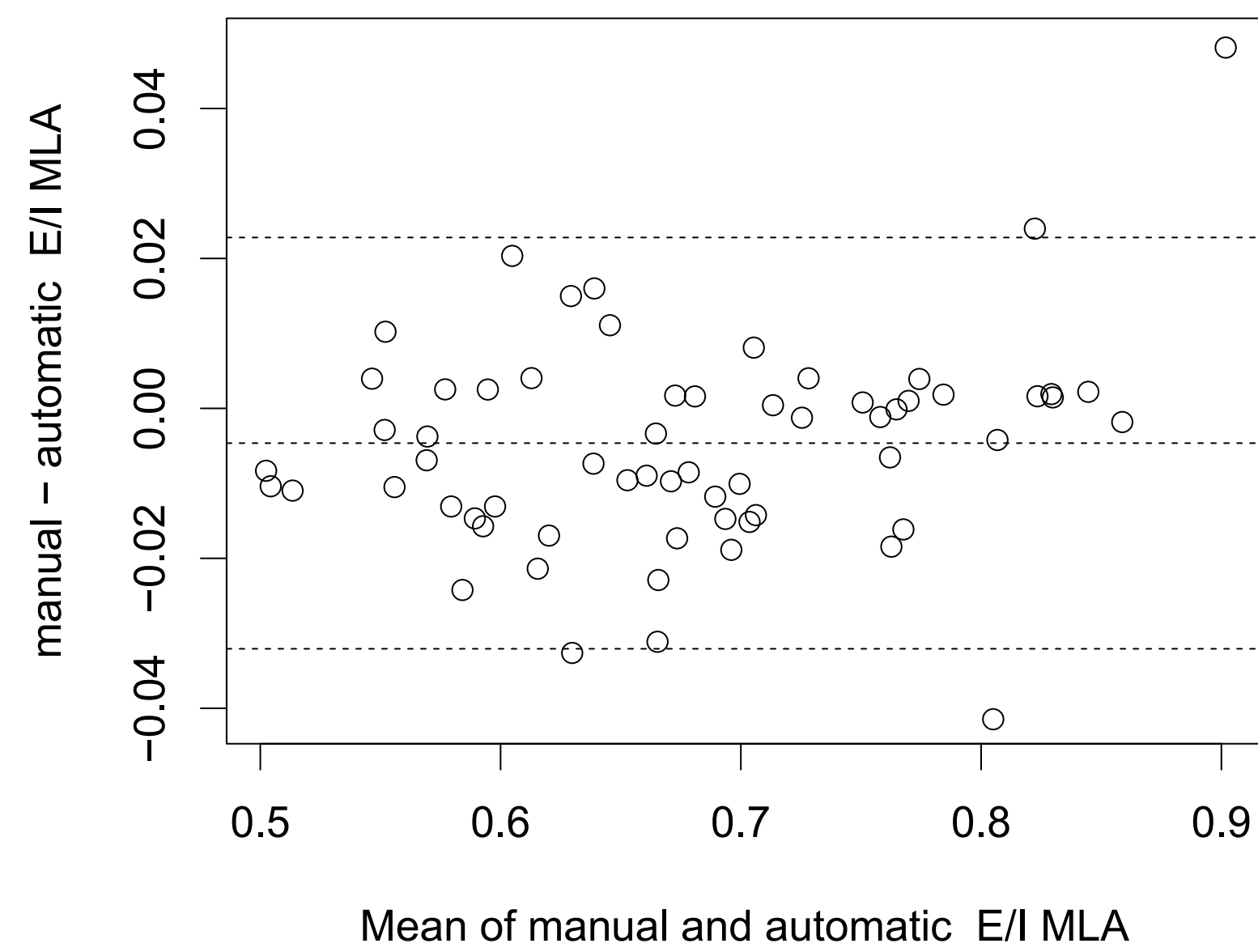**RML B30f**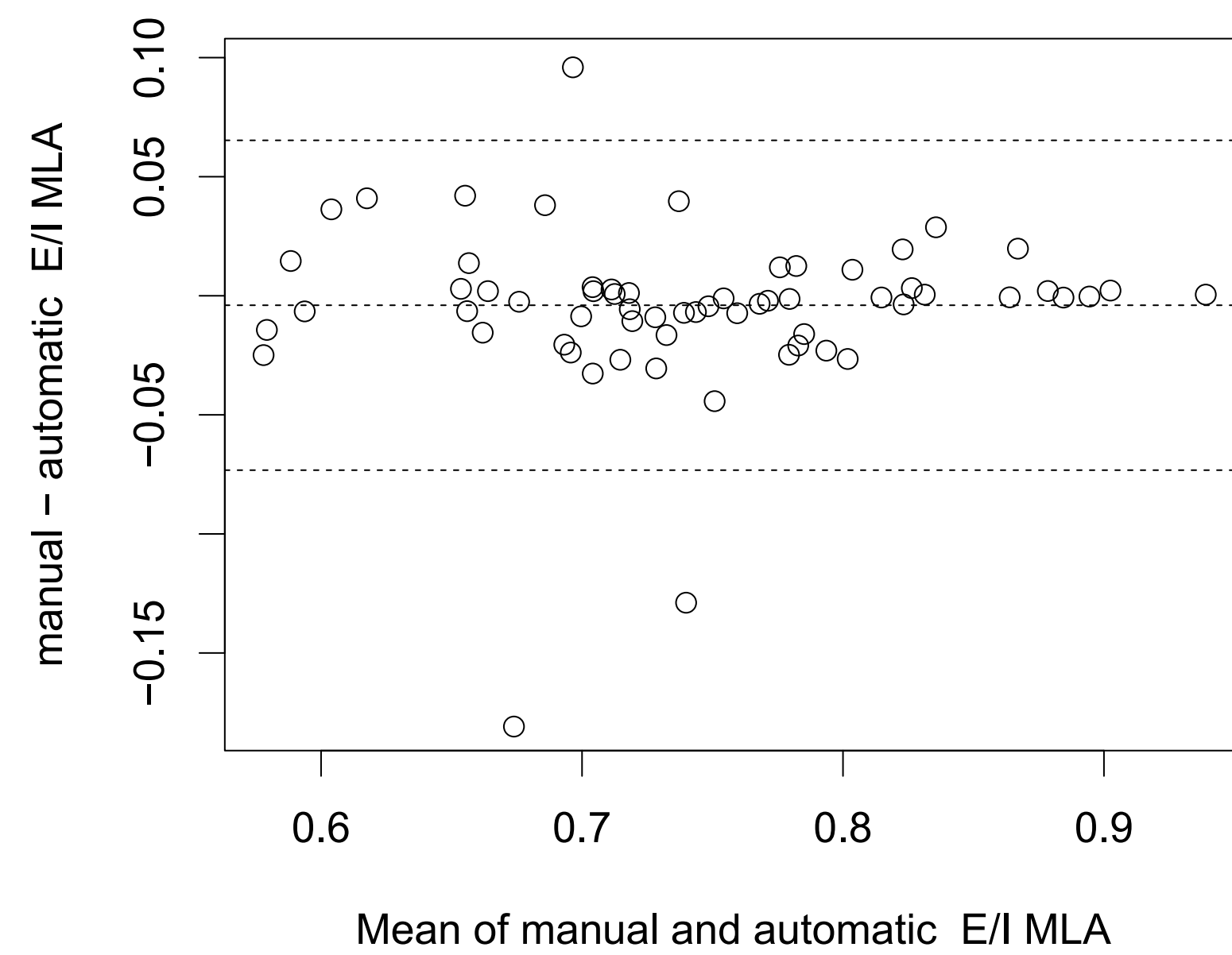**RLL B30f**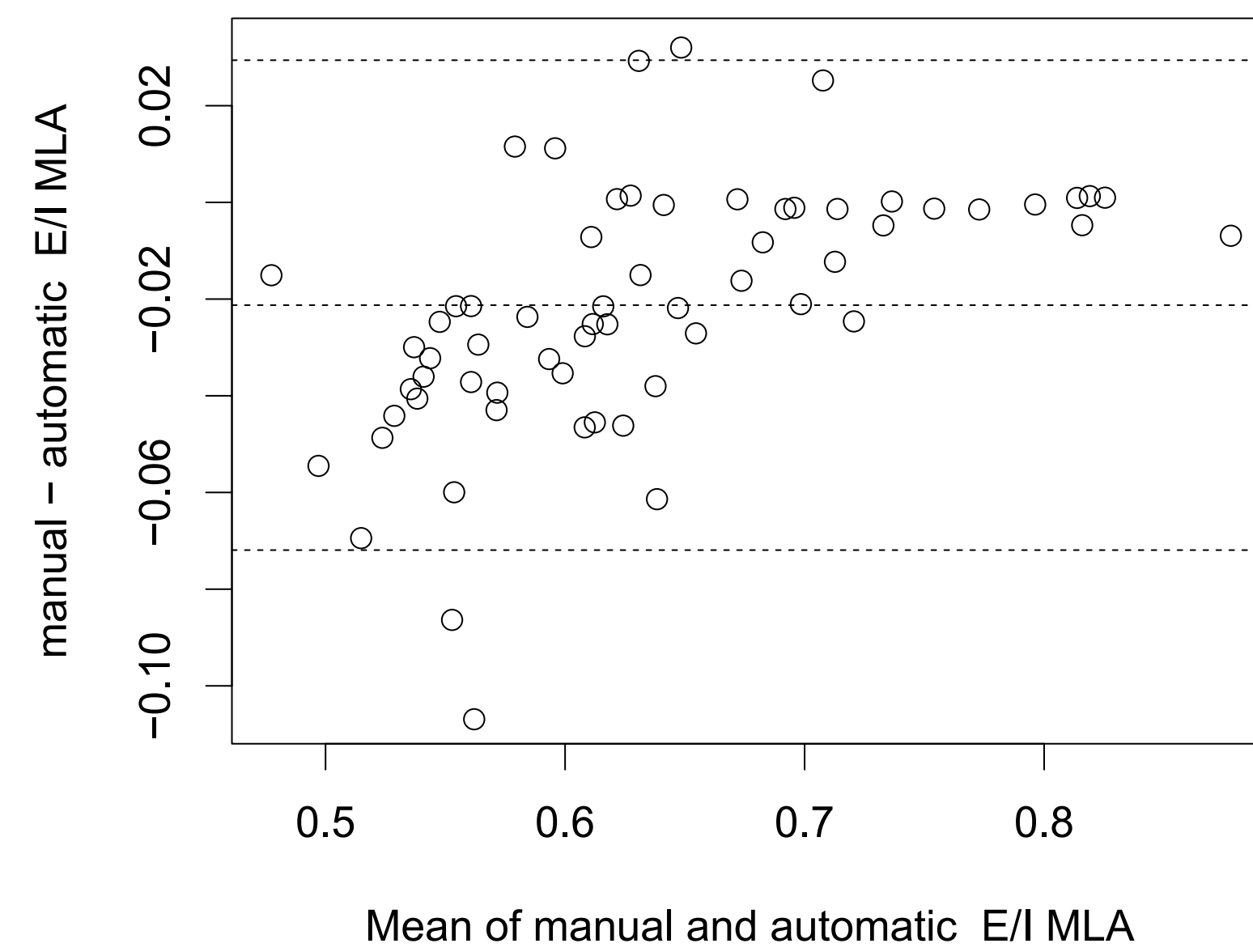**LUL B30f**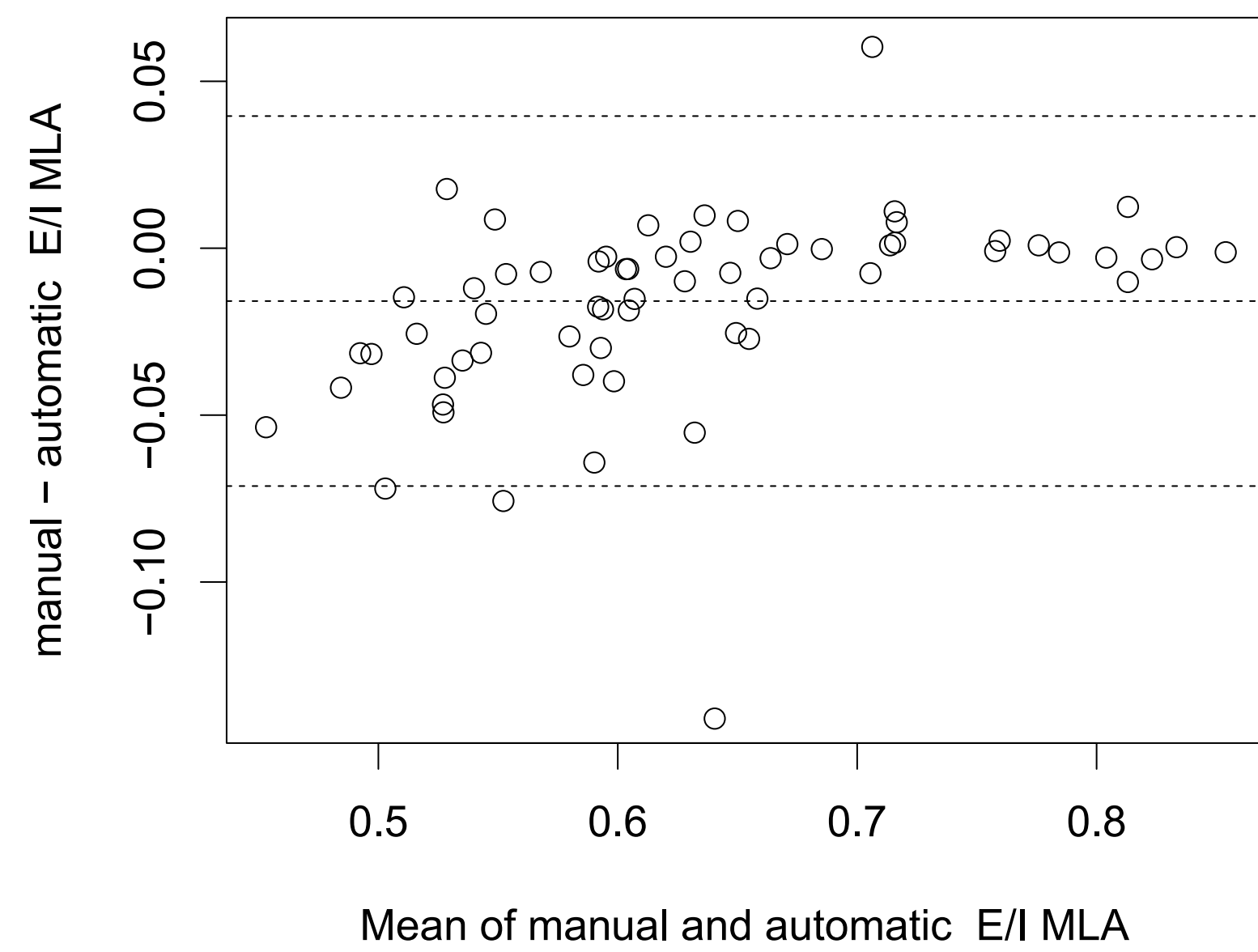**LLi B30f**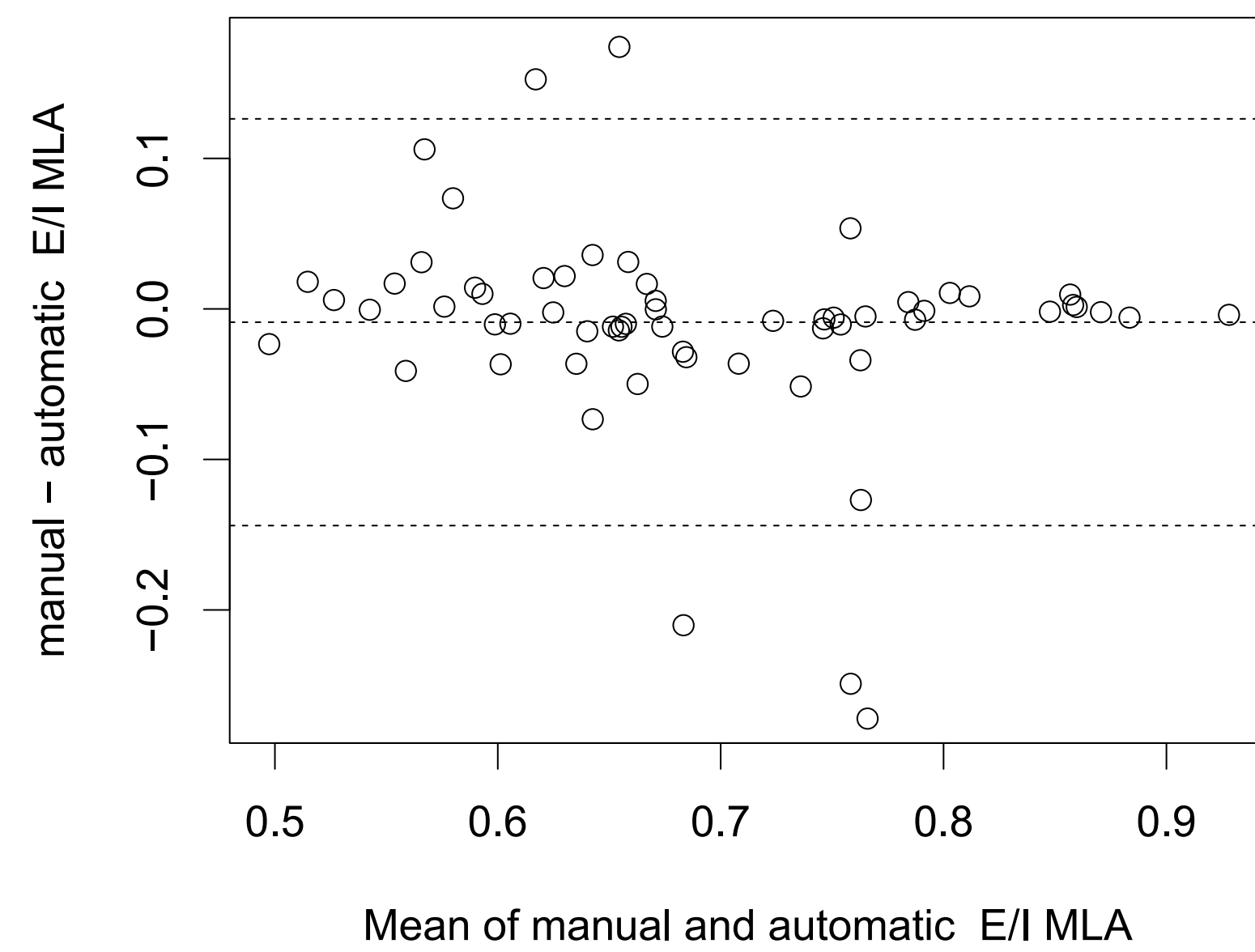**LLL B30f**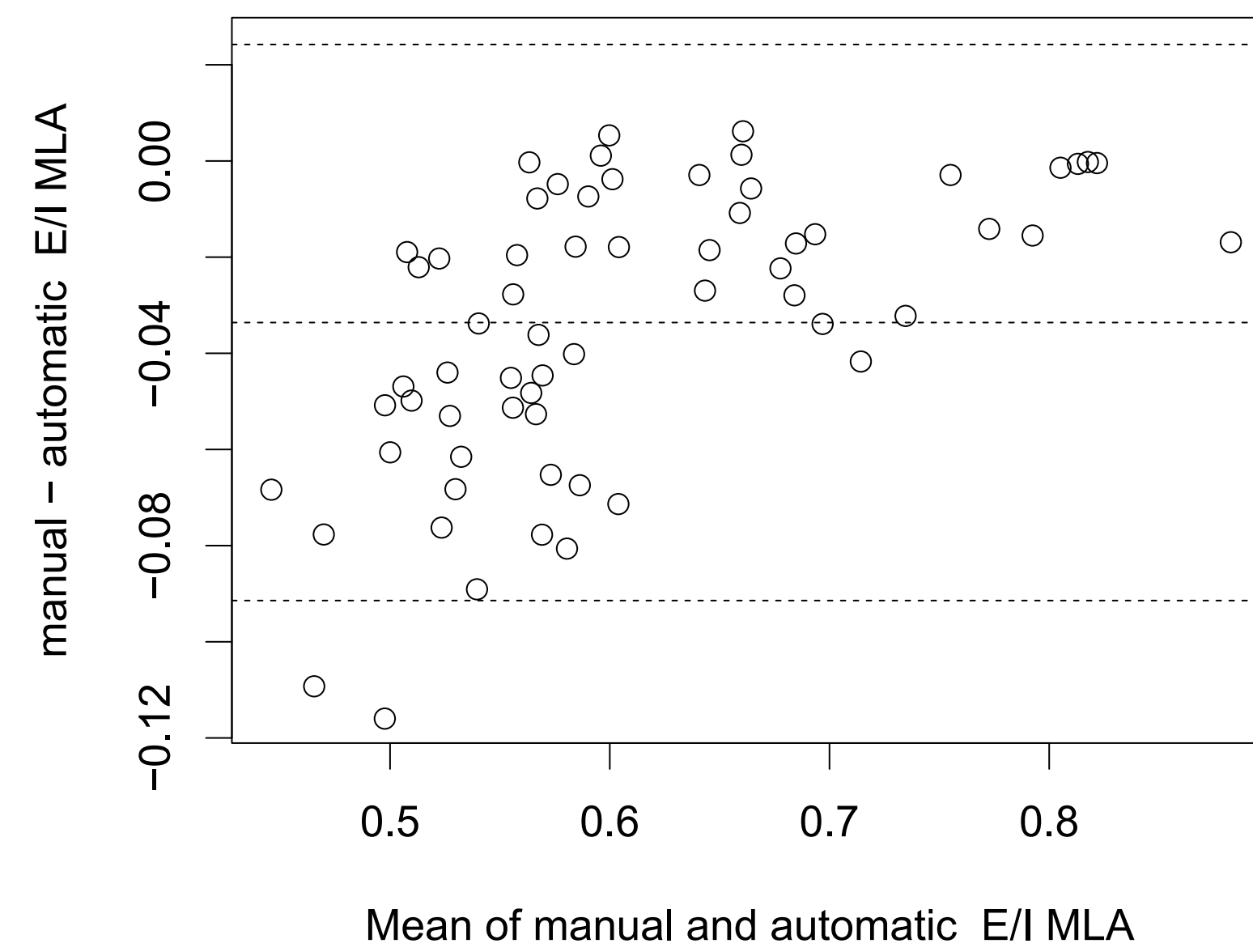**LUL+LLi B30f**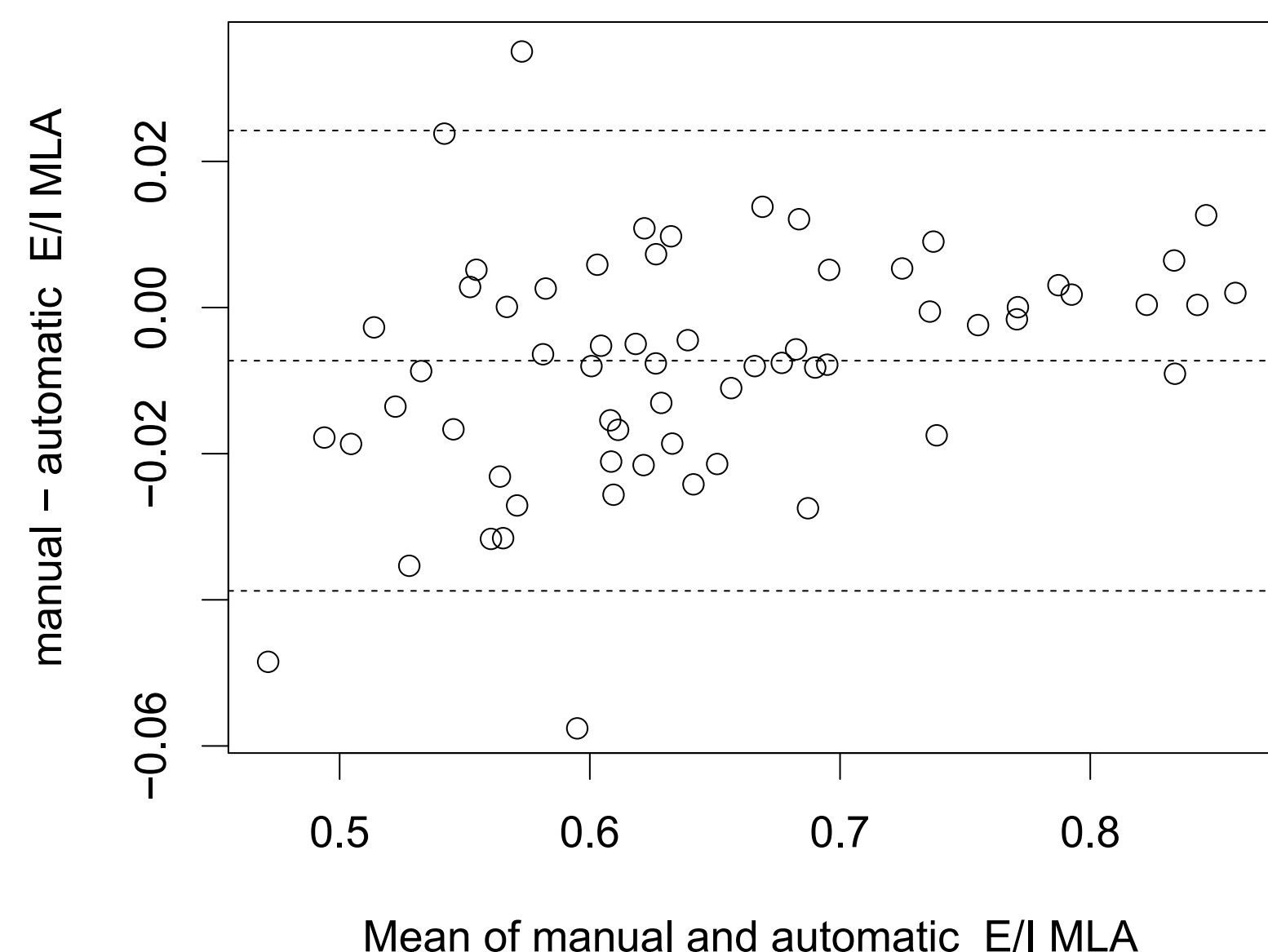

Supplement: S10 Fig — (PDF) [file pone.0194557.s010.pdf]

**RUL B60f**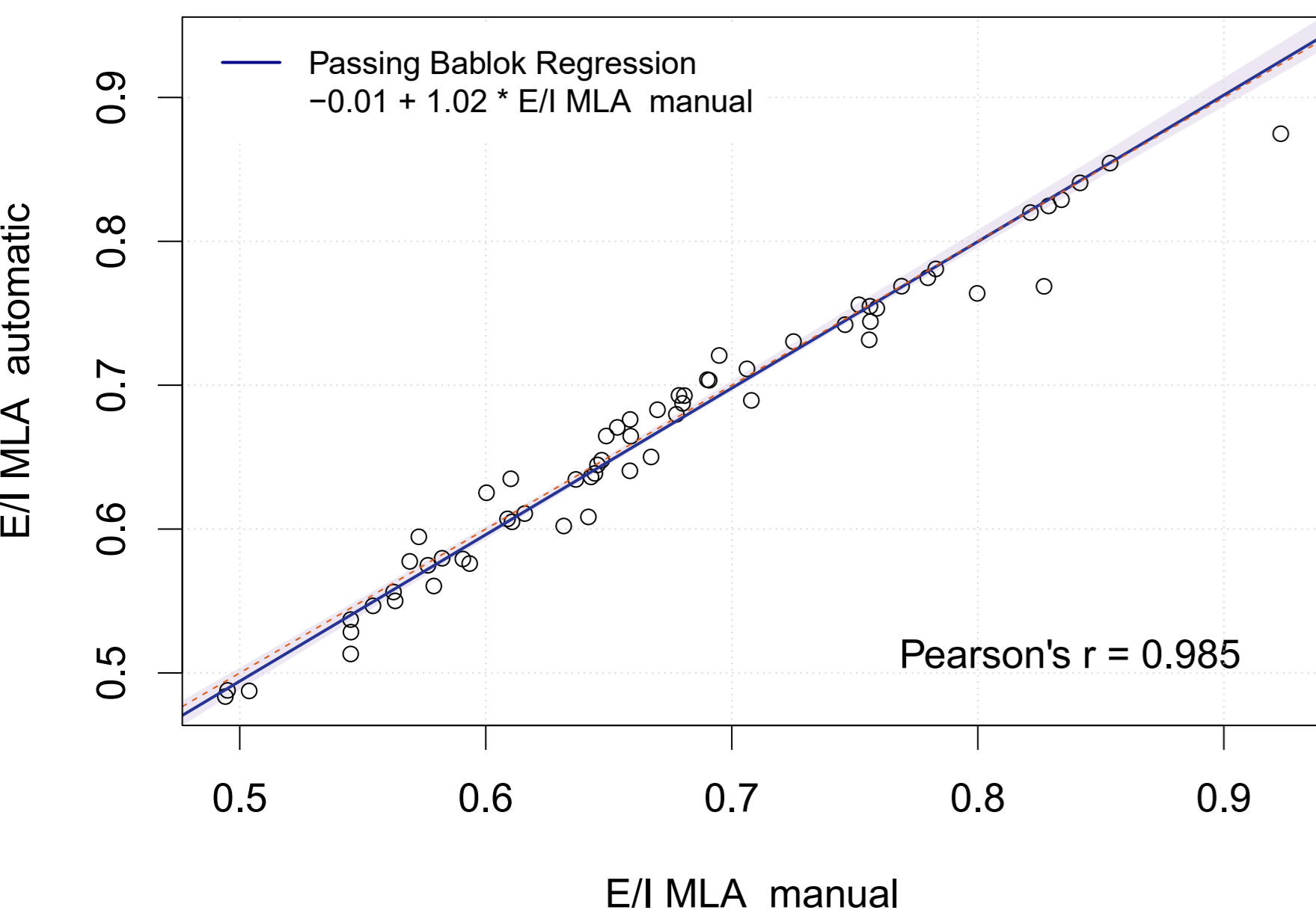**RML B60f**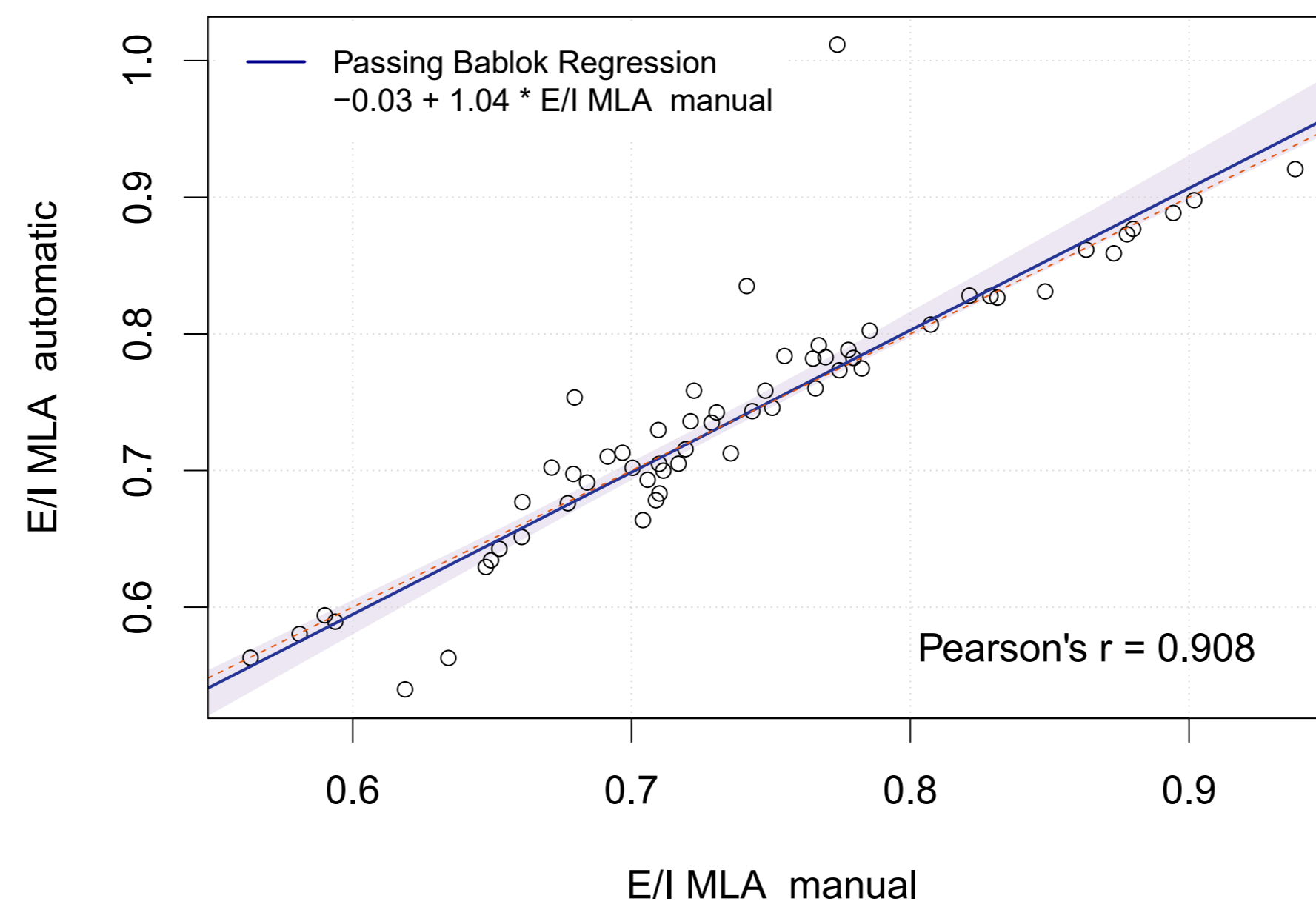**RLL B60f**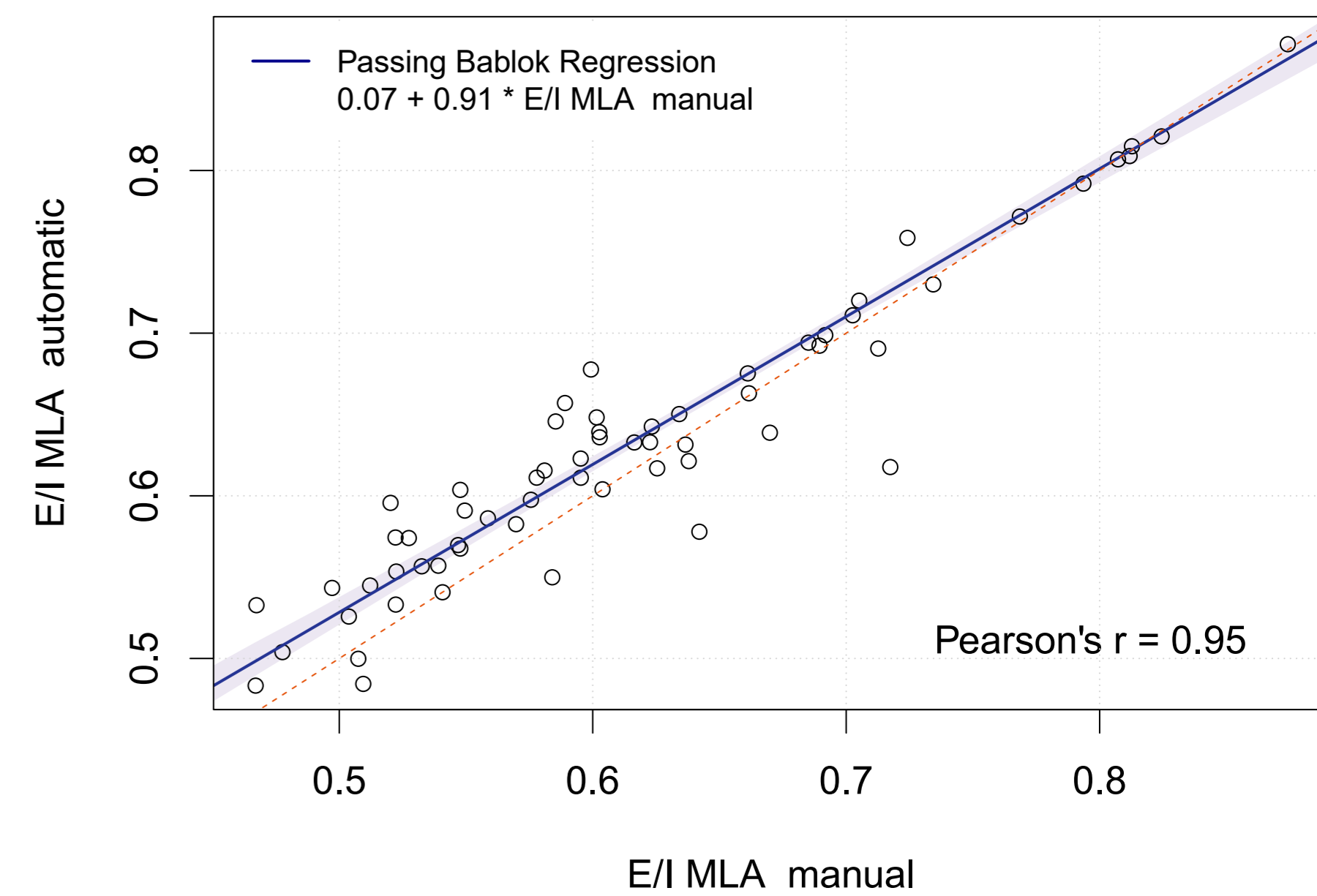**LUL B60f**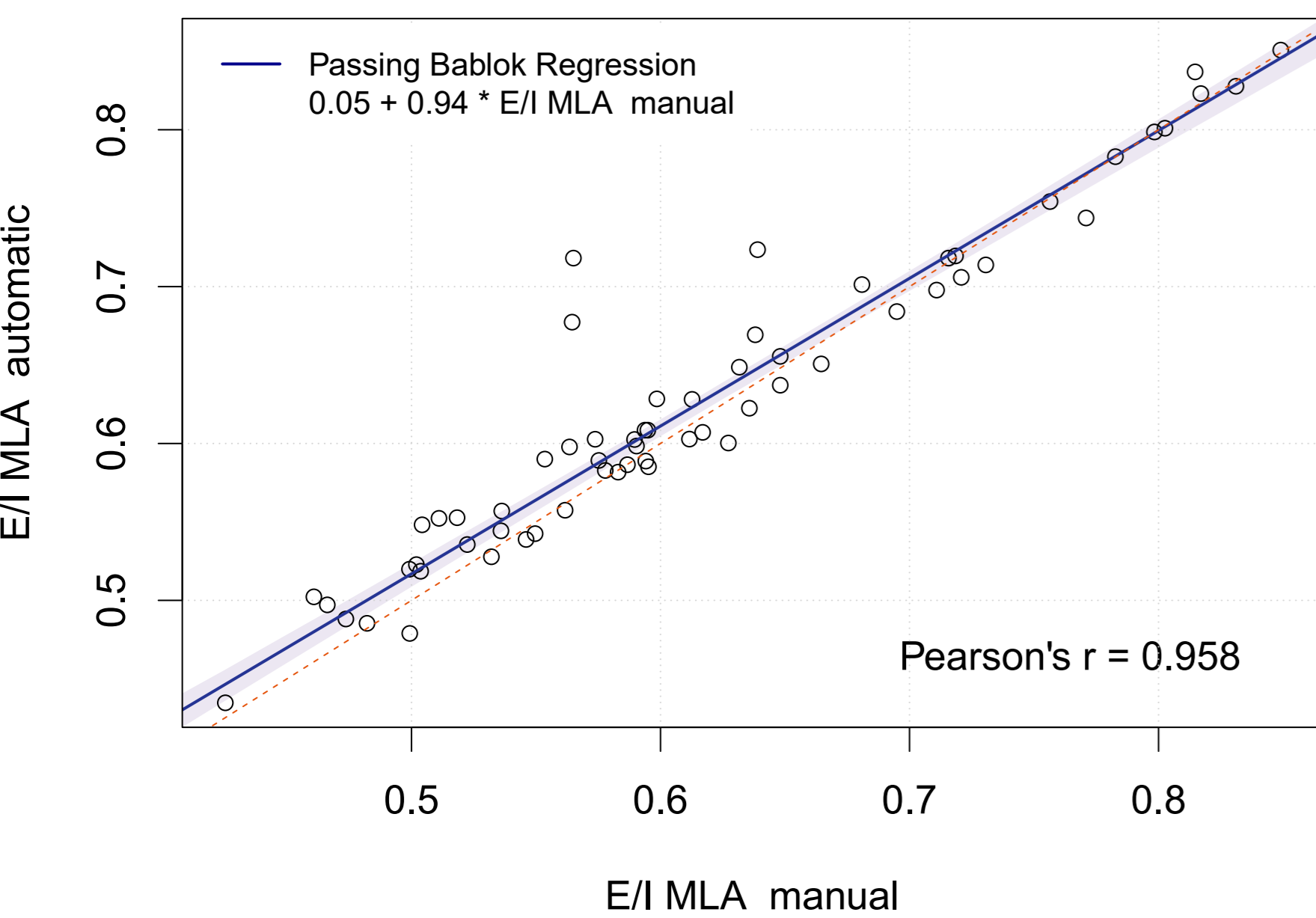**LLi B60f**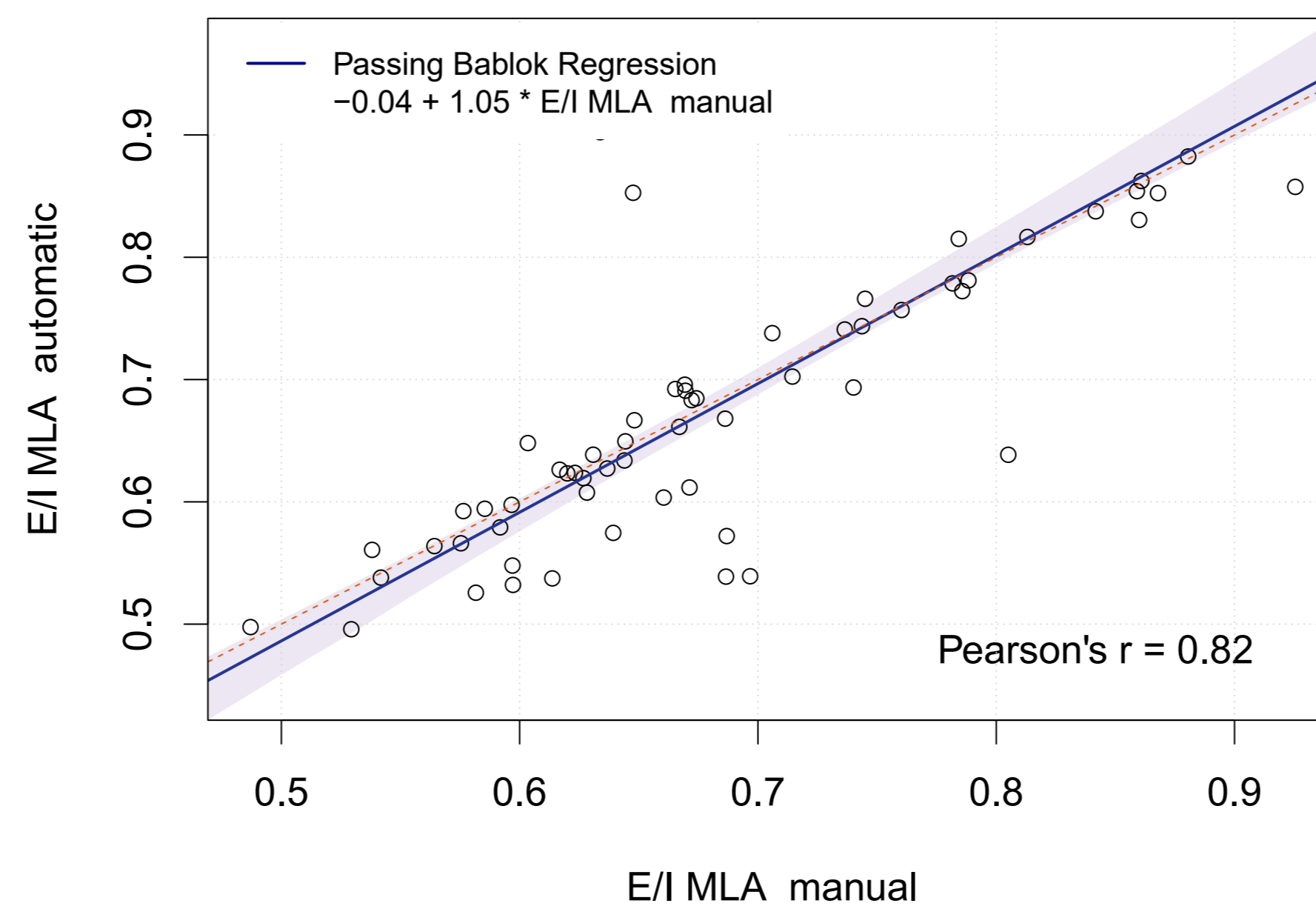**LLL B60f**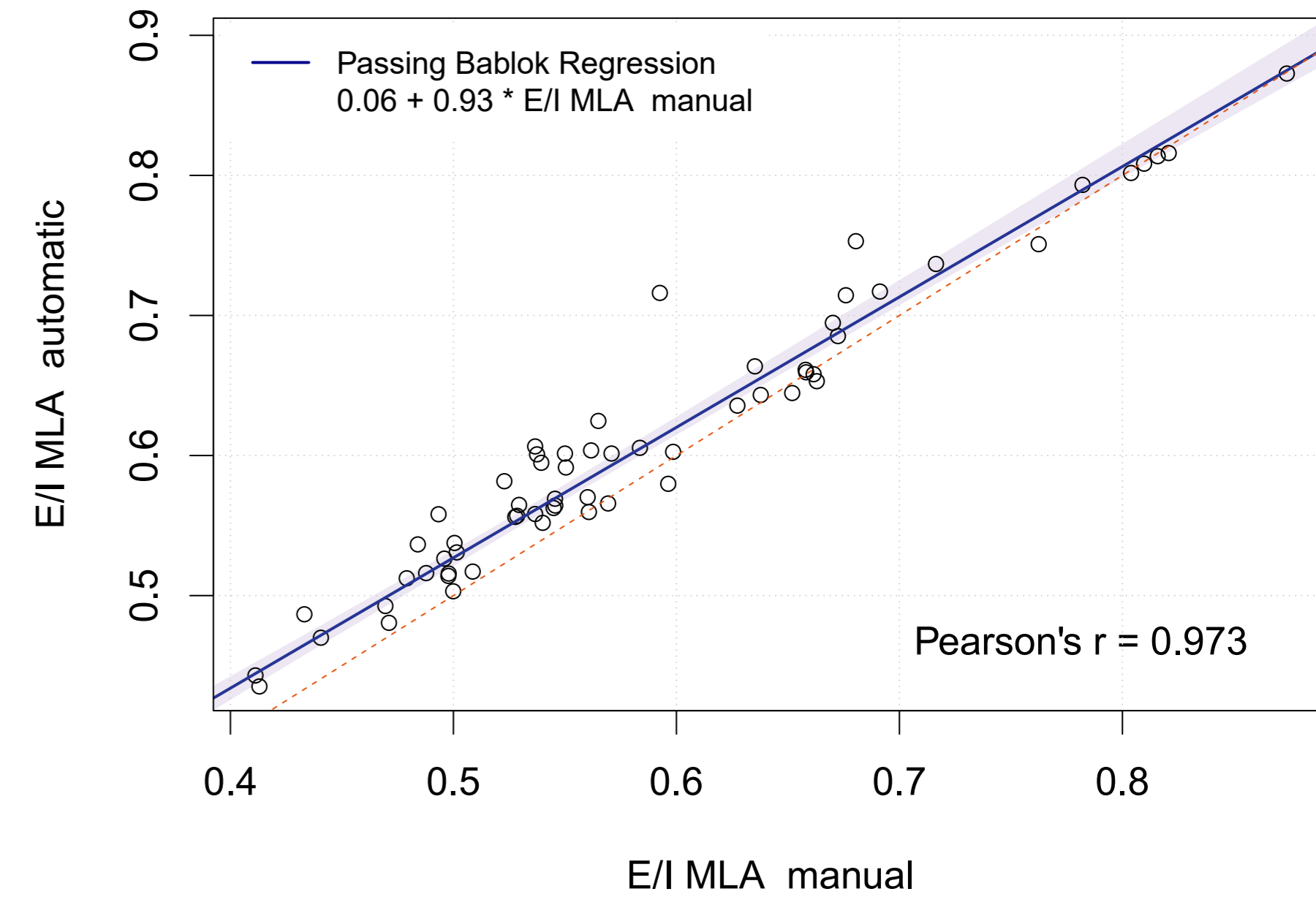**LUL+LLi B60f**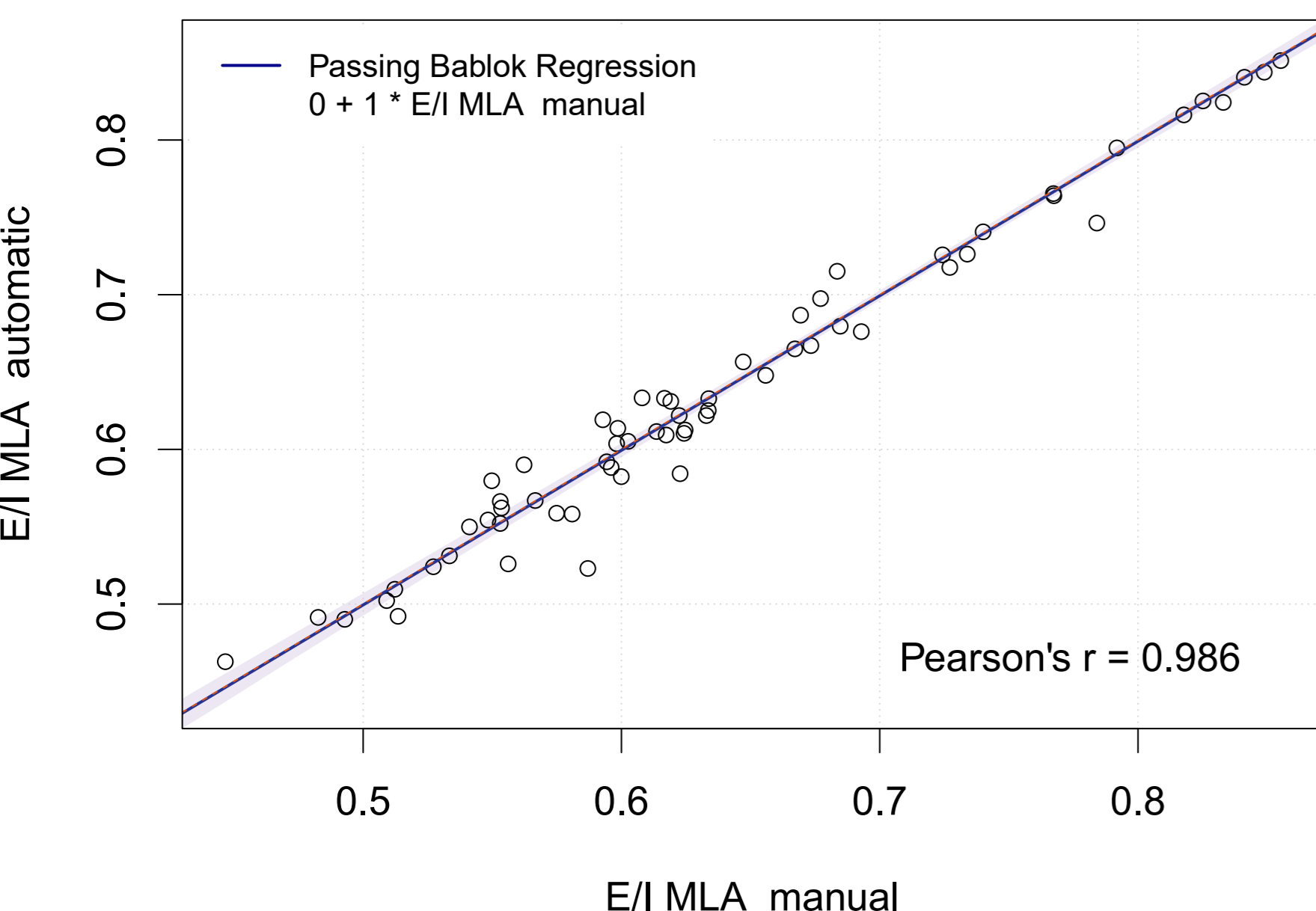

Supplement: S11 Fig — (PDF) [file pone.0194557.s011.pdf]

**RUL B60f**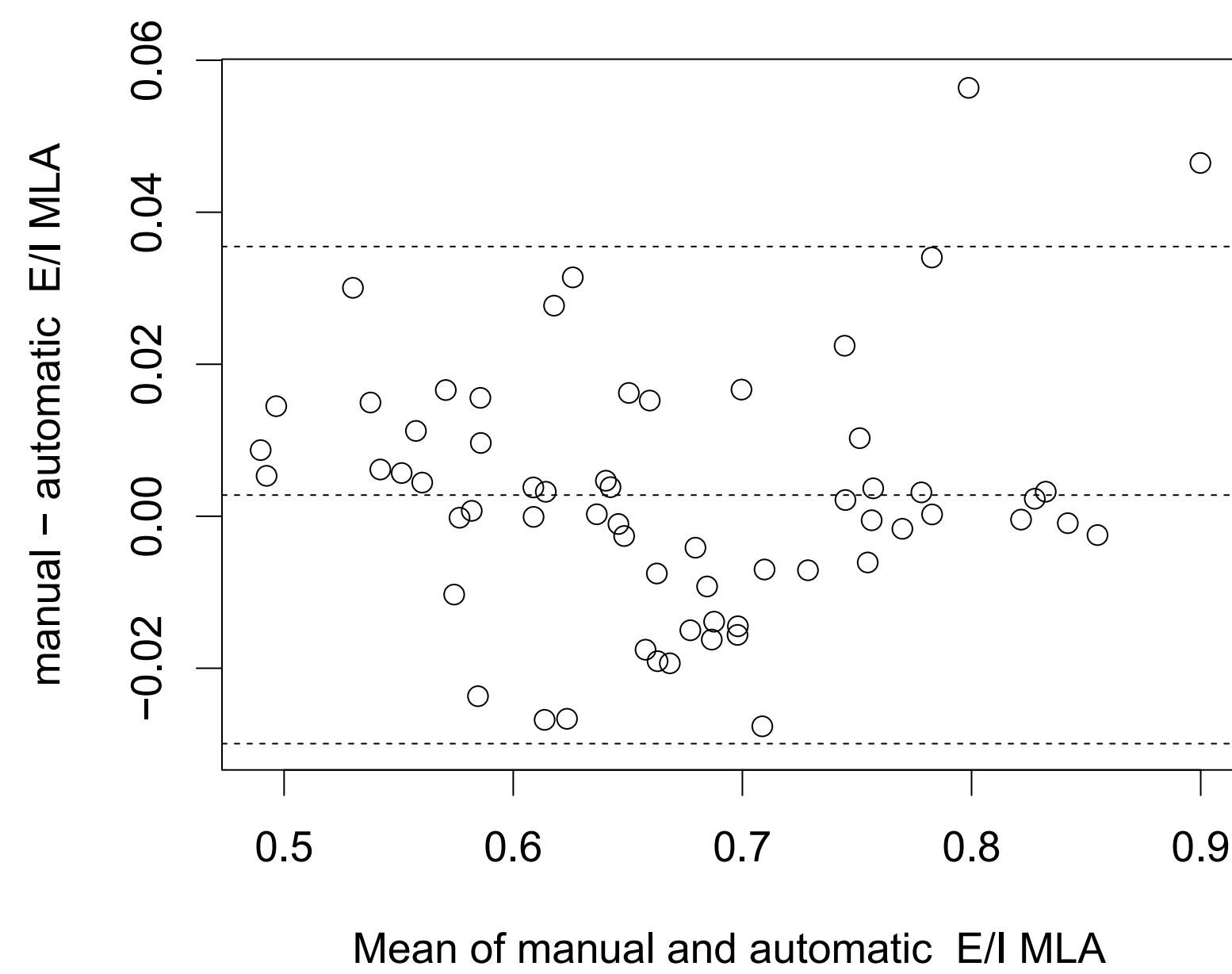**RML B60f**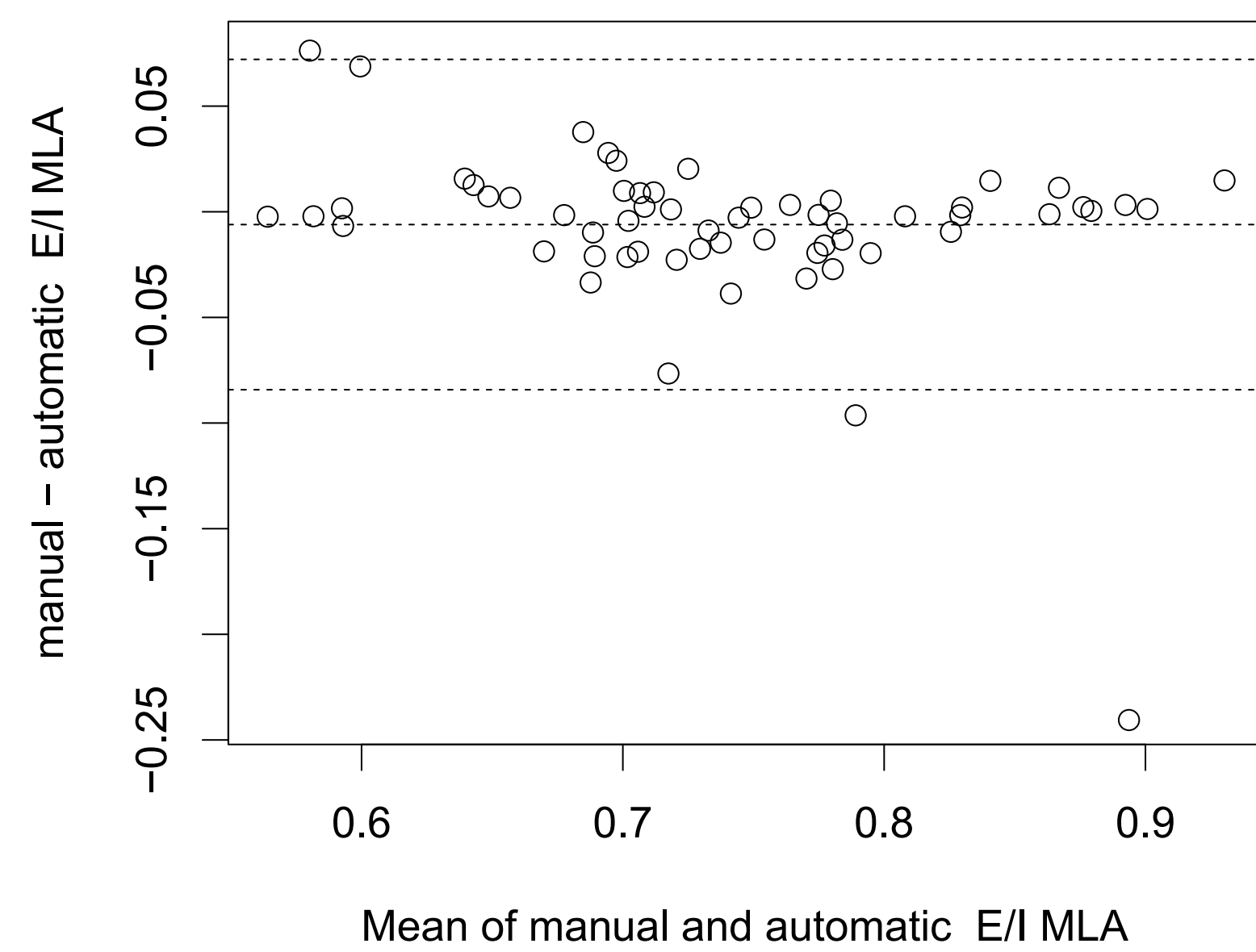**RLL B60f**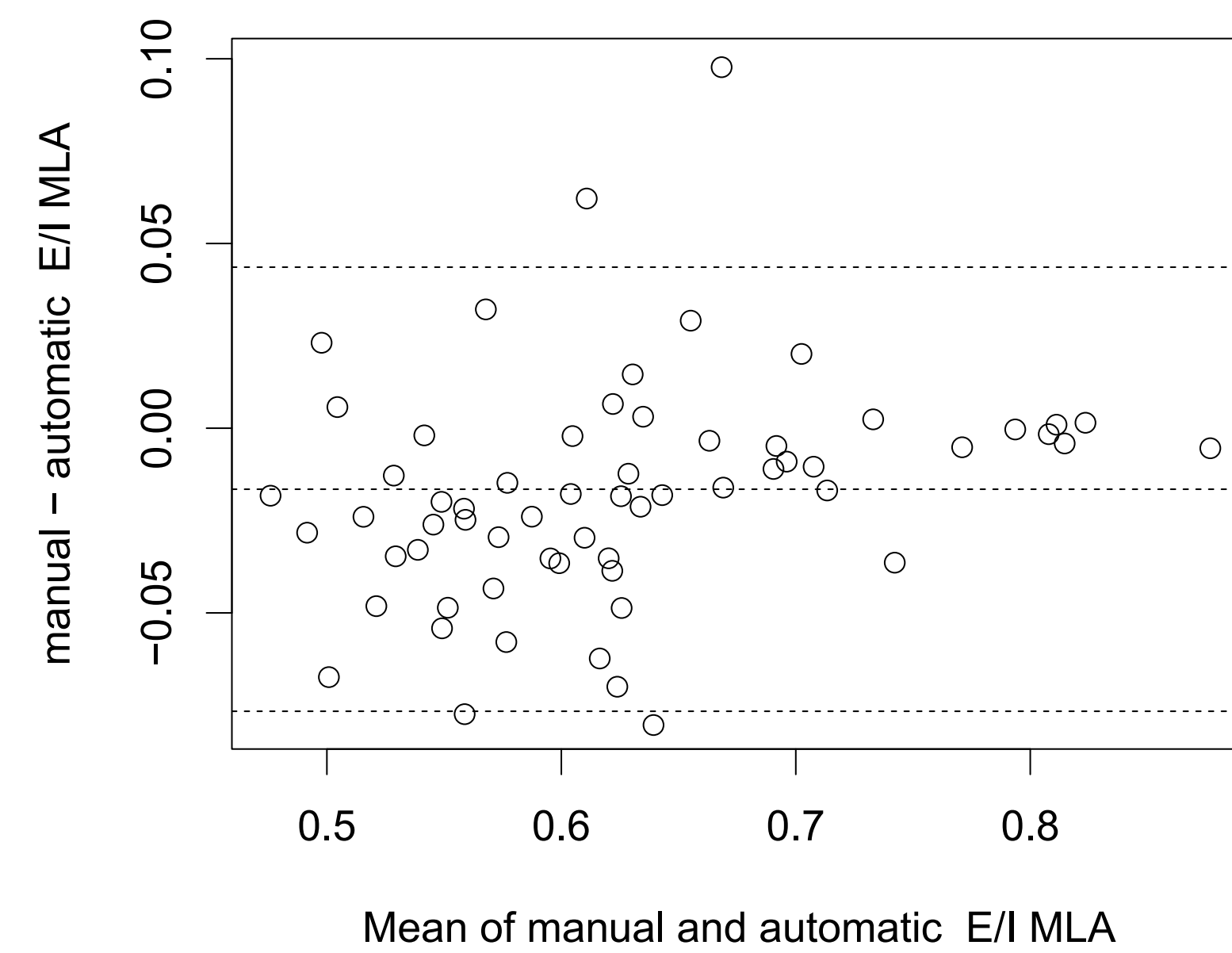**LUL B60f**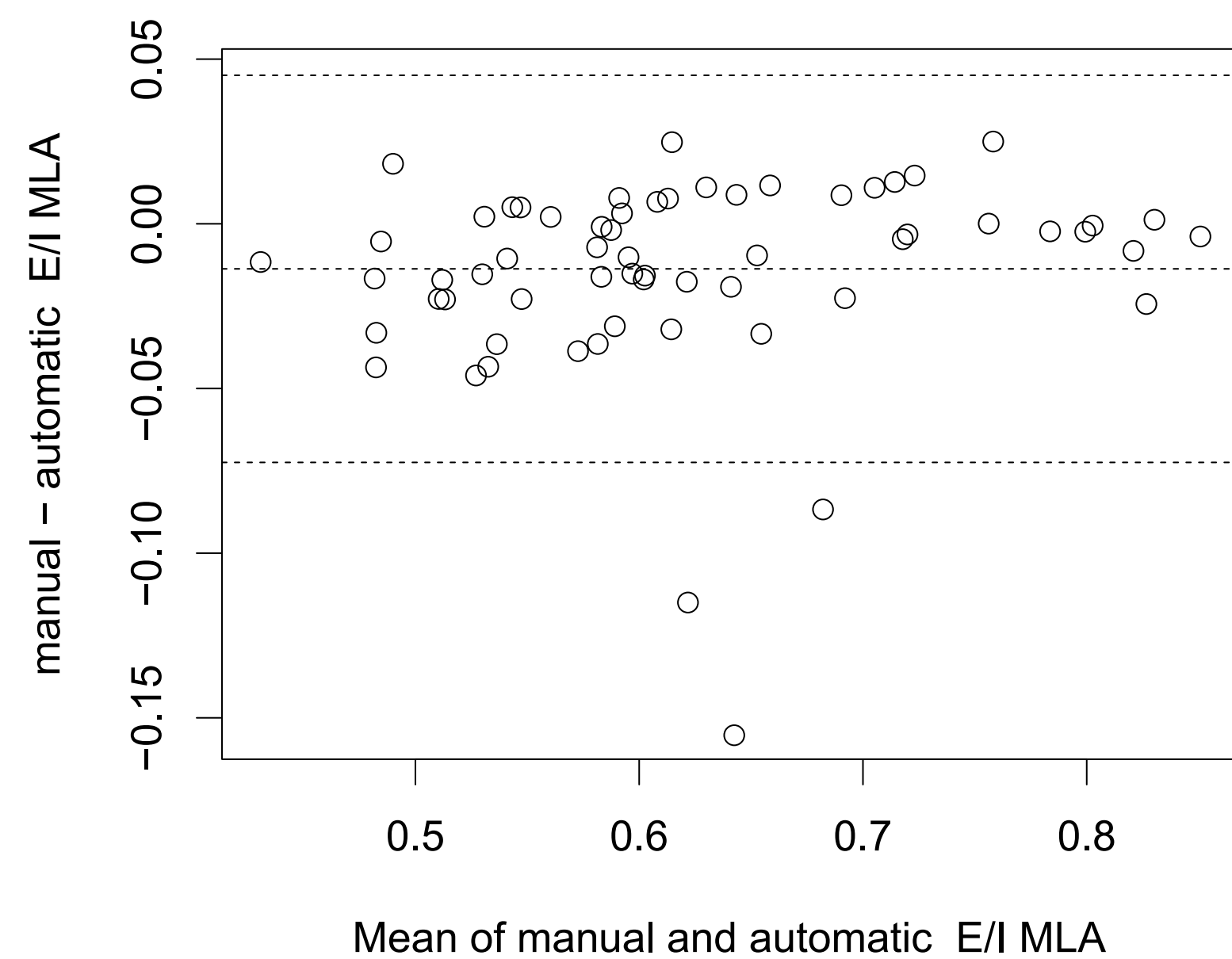**LLi B60f**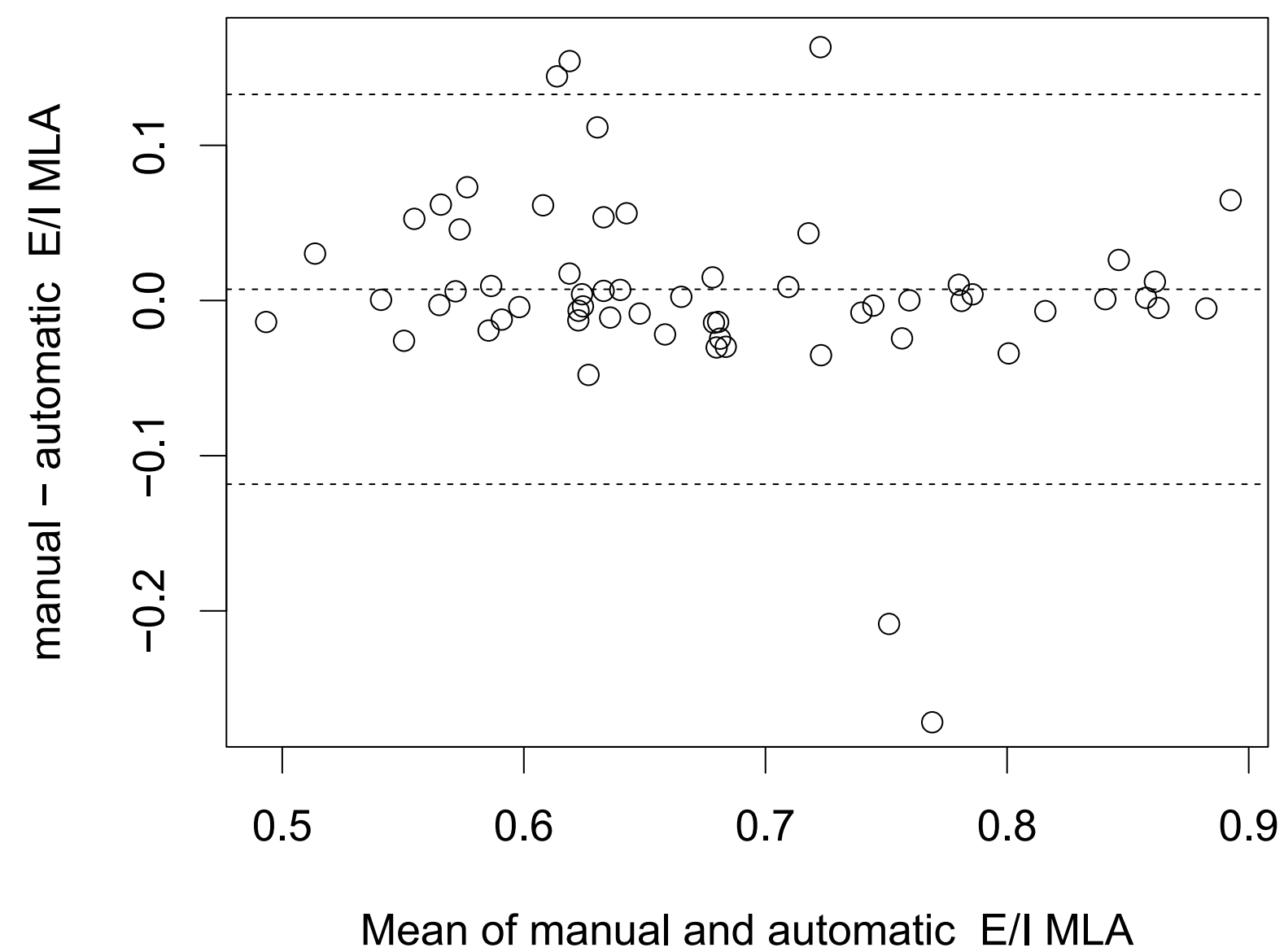**LLL B60f**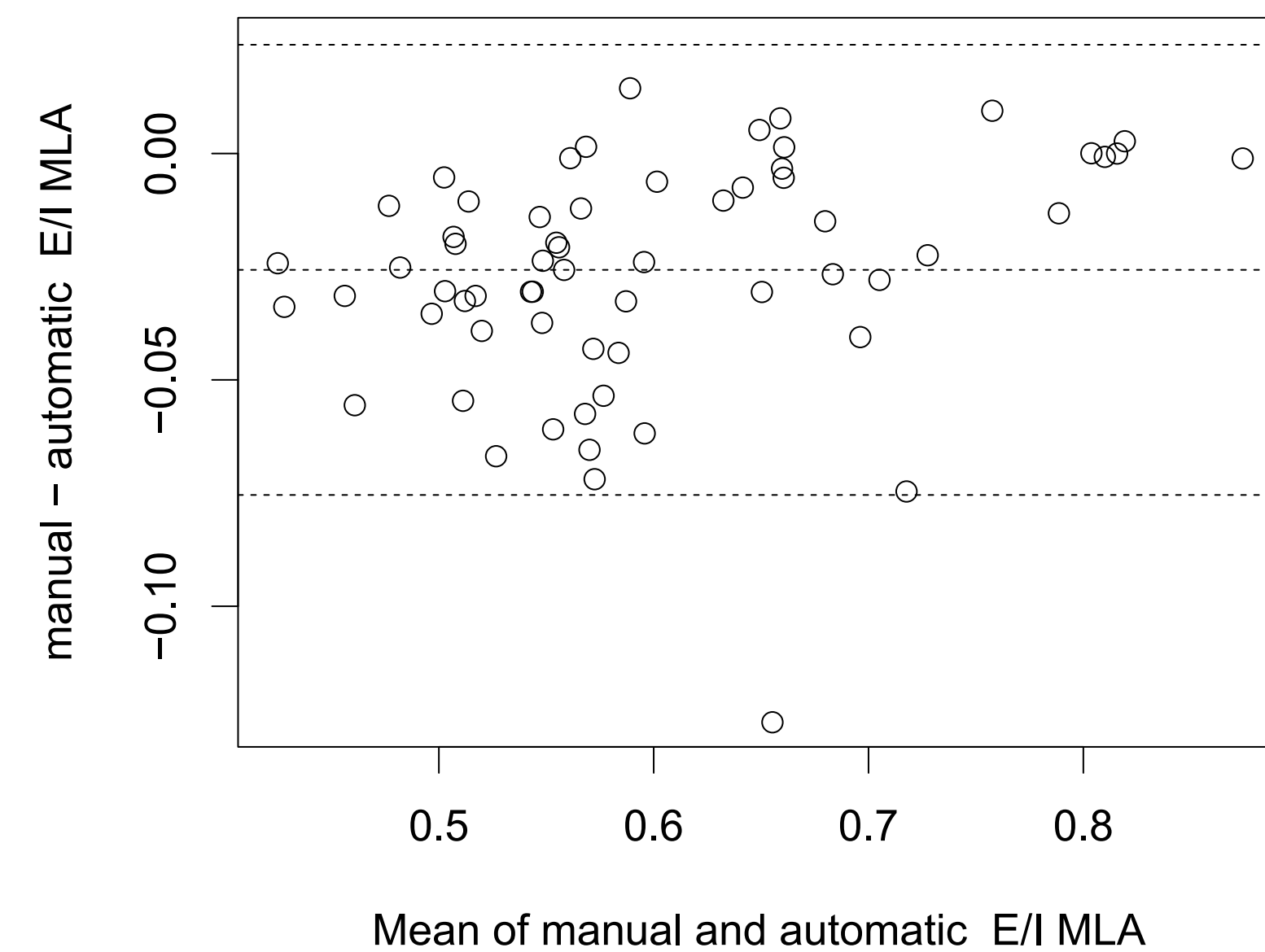**LUL+LLi B60f**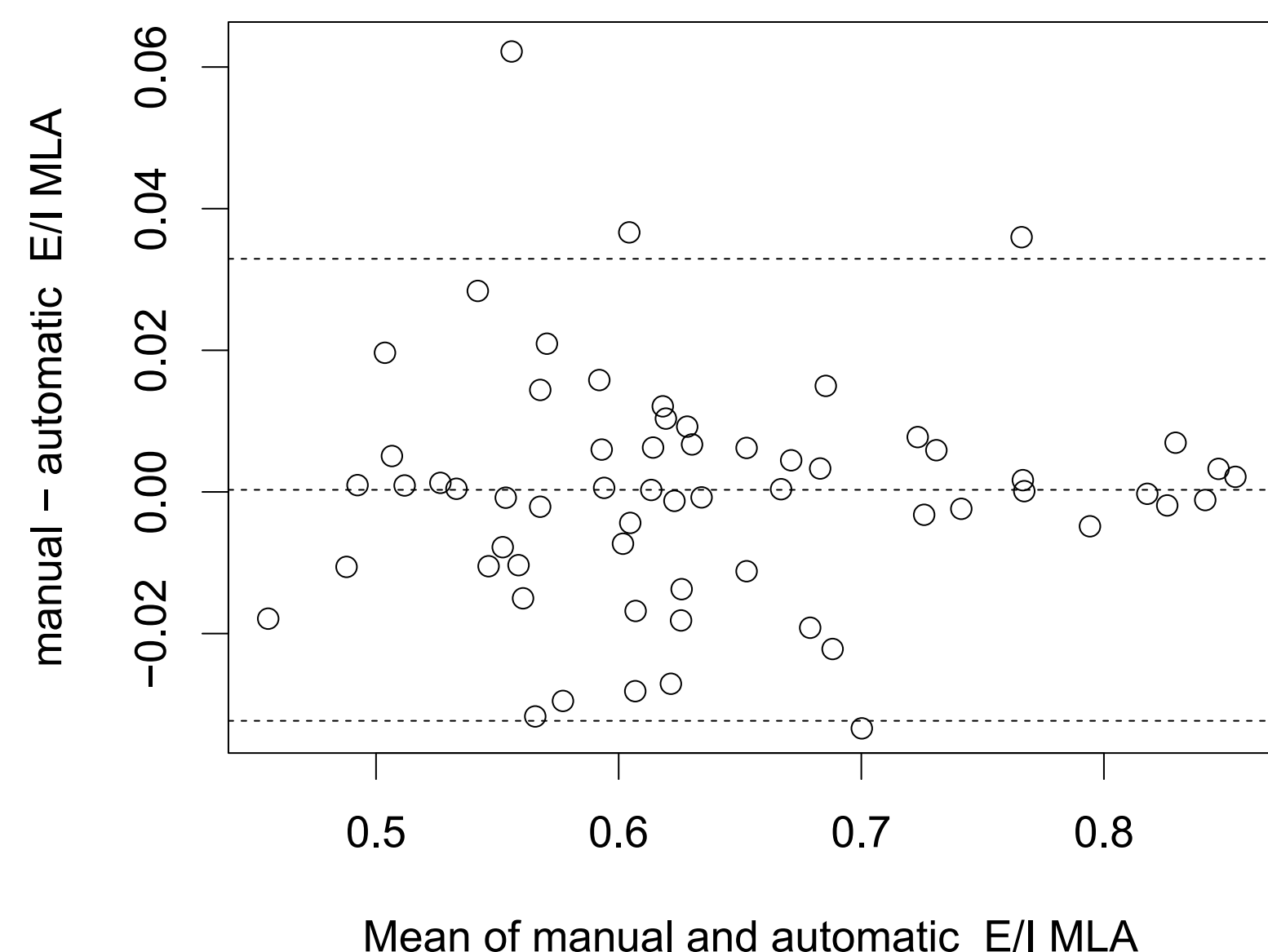

Supplement: S12 Fig — (PDF) [file pone.0194557.s012.pdf]
