# Supplementary material for: Validation of automated lobe segmentation on paired inspiratory-expiratory chest CT in 8-14 year-old children with cystic fibrosis
Source: PLoS One. 2018 Apr 9;13(4):e0194557. doi: 10.1371/journal.pone.0194557 (PMC5890971; doi:10.1371/journal.pone.0194557)
Supplement: S2 Table — Overlap calculated by the Jaccard index for fully automatic and manually corrected segmentation are given for inspiration (Insp) and expiration (Exp) and each for B30f and B60f kernel at baseline, 3, 12 and 24 months. The last column summarizes all time points. Mean and standard deviations (mean±sd) are separately calculated for the right upper (RUL), middle (RML) and lower lobe (RLL), the left upper lobe (LUL), the lingula (LLi), the left lower lobe (LLL), and also combining left upper lobe and lingula into one lobe (LUL+LLi). (PDF) [file pone.0194557.s014.pdf]

S2 Table. Jaccard index of overlap between fully automatic and manually corrected segmentation of lung.

**Jaccard index**			**Baseline**	**3 months**	**12 months**	**24 months**	**Overall**
**RUL**	Insp	B30f	0.96±0.02	0.95±0.02	0.95±0.03	0.96±0.03	0.95±0.02
	Insp	B60f	0.96±0.02	0.95±0.02	0.93±0.09	0.95±0.02	0.95±0.05
	Exp	B30f	0.72±0.17	0.79±0.12	0.79±0.09	0.82±0.11	0.78±0.13
	Exp	B60f	0.68±0.22	0.74±0.18	0.79±0.11	0.81±0.15	0.75±0.17
**RML**	Insp	B30f	0.89±0.03	0.87±0.04	0.89±0.03	0.87±0.07	0.88±0.05
	Insp	B60f	0.86±0.05	0.86±0.04	0.8±0.22	0.86±0.04	0.84±0.12
	Exp	B30f	0.66±0.22	0.66±0.23	0.6±0.29	0.73±0.16	0.66±0.23
	Exp	B60f	0.6±0.24	0.54±0.31	0.59±0.28	0.68±0.28	0.6±0.28
**RLL**	Insp	B30f	0.96±0.01	0.96±0.01	0.96±0.02	0.96±0.02	0.96±0.01
	Insp	B60f	0.95±0.02	0.95±0.02	0.94±0.03	0.96±0.01	0.95±0.02
	Exp	B30f	0.77±0.1	0.77±0.18	0.79±0.11	0.85±0.11	0.79±0.13
	Exp	B60f	0.77±0.17	0.74±0.17	0.78±0.13	0.8±0.16	0.77±0.16
**LUL**	Insp	B30f	0.93±0.02	0.9±0.09	0.91±0.03	0.92±0.04	0.92±0.05
	Insp	B60f	0.93±0.02	0.88±0.09	0.91±0.08	0.9±0.11	0.91±0.08
	Exp	B30f	0.6±0.21	0.69±0.15	0.74±0.13	0.74±0.14	0.69±0.16
	Exp	B60f	0.58±0.21	0.62±0.21	0.7±0.24	0.71±0.23	0.65±0.22
**LLi**	Insp	B30f	0.82±0.06	0.74±0.22	0.79±0.06	0.79±0.08	0.78±0.13
	Insp	B60f	0.78±0.12	0.68±0.2	0.74±0.21	0.73±0.21	0.73±0.19
	Exp	B30f	0.26±0.24	0.5±0.26	0.52±0.29	0.61±0.23	0.47±0.28
	Exp	B60f	0.32±0.29	0.38±0.28	0.46±0.34	0.61±0.2	0.44±0.29
**LLL**	Insp	B30f	0.97±0.01	0.94±0.1	0.96±0.03	0.96±0.02	0.96±0.05
	Insp	B60f	0.95±0.06	0.95±0.03	0.94±0.07	0.95±0.05	0.95±0.05
	Exp	B30f	0.67±0.15	0.73±0.16	0.78±0.14	0.82±0.14	0.75±0.16
	Exp	B60f	0.66±0.2	0.73±0.14	0.78±0.15	0.85±0.1	0.76±0.17
**LUL+LLi**	Insp	B30f	0.96±0.01	0.94±0.09	0.96±0.03	.96±0.02	0.95±0.05
	Insp	B60f	0.95±0.04	0.94±0.04	0.94±0.07	0.93±0.08	0.94±0.06
	Exp	B30f	0.69±0.11	0.77±0.11	0.8±0.12	0.84±0.1	0.78±0.12
	Exp	B60f	0.71±0.14	0.77±0.11	0.8±0.14	0.86±0.08	0.78±0.13

Overlap calculated by the Jaccard index for fully automatic and manually corrected segmentation are given for inspiration (Insp) and expiration (Exp) and each for B30f and B60f kernel at baseline, 3, 12 and 24 months. The last column summarizes all time points. Mean and standard

deviations (mean±sd) are separately calculated for the right upper (RUL), middle (RML) and lower lobe (RLL), the left upper lobe (LUL), the lingula (LLi), the left lower lobe (LLL), and also combining left upper lobe and lingula into one lobe (LUL+LLi).
