# Supplementary material for: Validation of automated lobe segmentation on paired inspiratory-expiratory chest CT in 8-14 year-old children with cystic fibrosis
Source: PLoS One. 2018 Apr 9;13(4):e0194557. doi: 10.1371/journal.pone.0194557 (PMC5890971; doi:10.1371/journal.pone.0194557)
Supplement: S4 Table — The indices of overlap summarized for 5 and 6 lobes at baseline, 3, 12, 24 month. Mean and standard deviation (mean±sd) are given for inspiration (Insp) and expiration (Exp) and each for B30f and B60f kernel. When treating LUL and LLi as a combined lobe (LUL+LLi, 5 lobes), the results were somewhat better than for separate analysis (LUL and LLi; 6 lobes), which is mainly due to the missing fissure, i.e. unequivocal separation, between both lobes. The relative difference between the two approaches are calculated as relative difference in [%]. (PDF) [file pone.0194557.s016.pdf]

S4 Table. Overlap comparison for 5 vs. 6 lobes.

		Dice index					Jaccard index					Hausdorff distance [mm]				
		Baseline	3 months	12 months	24 months	overall	Baseline	3 months	12 months	24 months	overall	Baseline	3 months	12 months	24 months	overall
**5 lobes**																
**Insp**	**B30f**	0.98±0.01	0.97±0.03	0.98±0.01	0.98±0.01	0.98±0.02	0.96±0.01	0.94±0.05	0.96±0.02	0.95±0.02	0.95±0.03	0.08±0.03	0.13±0.21	0.11±0.06	0.13±0.08	0.13±0.21
**Insp**	**B60f**	0.97±0.01	0.97±0.01	0.96±0.03	0.97±0.02	0.97±0.02	0.94±0.02	0.94±0.02	0.93±0.06	0.94±0.04	0.94±0.04	0.19±0.23	0.3±0.78	0.59±1.48	0.24±0.4	0.3±0.78
**Exp**	**B30f**	0.83±0.08	0.85±0.08	0.86±0.06	0.9±0.06	0.86±0.07	0.71±0.11	0.75±0.12	0.77±0.1	0.82±0.1	0.76±0.11	1.66±1.24	1.38±1.51	1.87±2.44	0.74±0.55	1.38±1.51
**Exp**	**B60f**	0.81±0.09	0.83±0.08	0.86±0.07	0.89±0.07	0.85±0.08	0.7±0.13	0.72±0.13	0.76±0.11	0.81±0.11	0.75±0.12	2.12±1.54	1.89±1.85	1.66±1.58	1.34±1.84	1.89±1.85
**6 lobes**																
**Insp**	**B30f**	0.97±0.02	0.96±0.04	0.97±0.01	0.97±0.01	0.97±0.02	0.94±0.01	0.92±0.07	0.94±0.02	0.94±0.02	0.93±0.04	0.16±0.08	0.59±1.44	0.21±0.09	0.24±0.13	0.3±0.74
**Insp**	**B60f**	0.96±0.03	0.95±0.02	0.95±0.04	0.96±0.03	0.96±0.03	0.93±0.02	0.91±0.03	0.91±0.07	0.92±0.05	0.92±0.05	0.28±0.33	0.49±0.54	0.9±1.78	0.62±1.51	0.57±1.19
**Exp**	**B30f**	0.83±0.08	0.83±0.09	0.84±0.07	0.88±0.07	0.83±0.08	0.66±0.12	0.72±0.13	0.74±0.1	0.79±0.11	0.72±0.12	3.46±2.54	1.79±1.42	2.11±2.34	1.07±0.87	2.11±2.06
**Exp**	**B60f**	0.81±0.1	0.79±0.09	0.83±0.09	0.86±0.09	0.81±0.1	0.64±0.15	0.66±0.12	0.72±0.12	0.76±0.13	0.7±0.14	3.76±2.97	3.53±2.86	3.11±2.81	1.85±2.02	3.06±2.73
**relative difference [%]**																
**Insp**	**B30f**	1.02	1.03	1.02	1.02	1.02	2.08	2.13	2.08	1.05	2.11	100.00	353.85	90.91	84.62	130.77
**Insp**	**B60f**	1.03	2.06	1.04	1.03	1.03	1.06	3.19	2.15	2.13	2.13	47.37	63.33	52.54	158.33	90.00
**Exp**	**B30f**	0.00	2.35	2.33	2.22	3.49	7.04	4.00	3.90	3.66	5.26	108.43	29.71	12.83	44.59	52.90
**Exp**	**B60f**	0.00	4.82	3.49	3.37	4.71	8.57	8.33	5.26	6.17	6.67	77.36	86.77	87.35	38.06	61.90

The indices of overlap summarized for 5 and 6 lobes at baseline, 3, 12 and 24 month. Mean and standard deviation (mean±sd) are given for inspiration (Insp) and expiration (Exp) and each for B30f and B60f kernel. When treating LUL and LLi as a combined lobe (LUL+LLi, 5 lobes), the results were somewhat better than for separate analysis (LUL and LLi; 6 lobes), which is mainly due to the missing fissure, i.e. unequivocal separation, between both lobes. The relative difference between the two approaches are calculated as relative difference in [%].
