# Supplementary material for: Validation of automated lobe segmentation on paired inspiratory-expiratory chest CT in 8-14 year-old children with cystic fibrosis
Source: PLoS One. 2018 Apr 9;13(4):e0194557. doi: 10.1371/journal.pone.0194557 (PMC5890971; doi:10.1371/journal.pone.0194557)
Supplement: S7 Table — Lobar volumes [cm3] calculated for fully automatic and manually corrected segmentation on expiration B30f scans at baseline, 3, 12 and 24 months. The last column summarizes all time points. All values are separately calculated for the right upper (RUL), middle (RML) and lower lobe (RLL), the left upper lobe (LUL), the lingula (LLi), the left lower lobe (LLL), and also combining left upper lobe and lingula into one lobe (LUL+LLi). Both methods are compared in accordance with the approach of Bland-Altman giving mean differences (Δ), limits of agreement (LoA) and two regression coefficients (Intercept / Slope and Pearson’s correlation coefficient). (PDF) [file pone.0194557.s019.pdf]

S7 Table. Lobe volume determination on manual and automatic segmentation maps (Expiration B30f scans).

		Baseline	3 months	12 months	24 months	Overall
RUL	manually corrected (mean±sd)	230.34±86.44	245.4±94.3	270.53±101.51	310.56±114.78	264.21±102.09
	automatic (mean±sd)	221.6±101.11	272.66±127.78	296.12±142.28	333.54±141.23	280.98±132.53
	Δ (mean±sd)	8.74±47.35	-27.25±50.32	-25.59±59.02	-22.99±60.4	-16.77±55.29
	LoA (2.5% limit, 97.5% limit)	(-84.07, 101.55)	(-125.89, 71.38)	(-141.26, 90.08)	(-141.38, 95.4)	(-125.15, 91.61)
	Regr. (Intercept / Slope)	-20.49 / 1.1	-30.91 / 1.22	-67.18 / 1.36	-27.02 / 1.12	-39.14 / 1.2
	Regr (Pearson's r)	0.88	0.94	0.94	0.91	0.92
RML	manually corrected (mean±sd)	130.01±39.26	134.6±59.44	144.17±45.9	153.96±43.4	140.68±47.38
	automatic (mean±sd)	142.01±51.3	143.9±63.19	117.44±62.78	161.06±47.13	141.1±57.36
	Δ (mean±sd)	-12.01±41.1	-9.3±48	26.73±69.6	-7.1±37.82	-0.42±51.94
	LoA (2.5% limit, 97.5% limit)	(-92.57, 68.56)	(-103.39, 84.79)	(-109.69, 163.15)	(-81.24, 67.04)	(-102.22, 101.38)
	Regr. (Intercept / Slope)	-52.59 / 1.52	-26.48 / 1.29	-201.2 / 2.28	5.86 / 1.03	-23.03 / 1.25
	Regr (Pearson's r)	0.62	0.7	0.21	0.65	0.52
RLL	manually corrected (mean±sd)	336.77±92.61	349.91±146.68	371.21±115.95	426.88±148.03	371.19±129.56
	automatic (mean±sd)	307.01±96.2	295.38±168.41	357.65±122.39	381.76±161.57	335.45±141.5
	Δ (mean±sd)	29.77±41.82	54.53±44.44	13.56±60.63	45.13±67.2	35.75±55.53
	LoA (2.5% limit, 97.5% limit)	(-52.21, 111.74)	(-32.57, 141.64)	(-105.27, 132.39)	(-86.59, 176.85)	(-73.09, 144.58)
	Regr. (Intercept / Slope)	-63 / 1.08	-91.24 / 1.11	-91.04 / 1.2	-105.82 / 1.17	-83.58 / 1.14
	Regr (Pearson's r)	0.9	0.97	0.87	0.91	0.92
LUL	manually corrected (mean±sd)	171.74±67.96	171.37±68.27	205.36±83.97	230.07±89.51	194.63±80.09
	automatic (mean±sd)	202.62±135.81	188.62±106.06	207.49±77.1	245.08±119.24	210.95±110.97
	Δ (mean±sd)	-30.88±103.34	-17.26±67.65	-2.13±44.88	-15.01±57.54	-16.32±70.75
	LoA (2.5% limit, 97.5% limit)	(-233.43, 171.66)	(-149.85, 115.34)	(-90.09, 85.82)	(-127.78, 97.77)	(-154.99, 122.35)
	Regr. (Intercept / Slope)	-241.03 / 2.36	-61.6 / 1.43	-18.32 / 1.13	-15.48 / 1.08	-83.58 / 1.28
	Regr (Pearson's r)	0.67	0.78	0.85	0.89	0.77
LLi	manually corrected (mean±sd)	95.25±31.27	108.06±49.29	113.47±35.12	123.31±36.67	109.97±39.16
	automatic (mean±sd)	59.22±56.02	90.76±51.6	98.77±69.84	121.04±53.64	92.35±60.89
	Δ (mean±sd)	36.03±62.92	17.3±47.28	14.71±53.59	2.27±43.33	17.62±52.46
	LoA (2.5% limit, 97.5% limit)	(-87.28, 159.35)	(-75.36, 109.97)	(-90.33, 119.74)	(-82.67, 87.2)	(-85.19, 120.44)
	Regr. (Intercept / Slope)	-160.85 / 2.21	-26.97 / 1.2	-142.94 / 2.12	-31.91 / 1.36	-23.03 / 1.67
	Regr (Pearson's r)	0.05	0.56	0.66	0.6	0.52
LLL	manually corrected (mean±sd)	295.23±93.47	301.04±126.38	327.82±120.76	370.27±128.35	323.59±119.03
	automatic (mean±sd)	262.48±129.73	273.39±151.35	325.67±124.01	342.09±138.49	300.91±137.27
	Δ (mean±sd)	32.75±76	27.65±44.65	2.14±44.25	28.18±39.68	22.68±53.27
	LoA (2.5% limit, 97.5% limit)	(-116.21, 181.71)	(-59.87, 115.17)	(-84.58, 88.87)	(-49.59, 105.95)	(-81.73, 127.09)
	Regr. (Intercept / Slope)	-91.39 / 1.29	-103.63 / 1.22	-13.45 / 1	-51.17 / 1.09	-39.14 / 1.14
	Regr (Pearson's r)	0.82	0.96	0.93	0.96	0.92
LUL+LLi	manually corrected (mean±sd)	266.99±92.51	279.43±114.16	316.59±115.64	353.38±122.37	304.1±114.24
	automatic (mean±sd)	261.84±112.72	279.39±121.71	300.08±116.84	366.12±136.05	301.86±125.68
	Δ (mean±sd)	5.15±68.16	0.05±36.81	16.51±43.88	-12.74±36.18	2.24±48.07
	LoA (2.5% limit, 97.5% limit)	(-128.44, 138.74)	(-72.1, 72.19)	(-69.5, 102.51)	(-83.65, 58.17)	(-91.98, 96.46)
	Regr. (Intercept / Slope)	-67.32 / 1.18	-27.34 / 1.06	-68.35 / 1.24	-32.77 / 1.12	-50.14 / 1.15
	Regr (Pearson's r)	0.8	0.95	0.93	0.97	0.92

Lobar volumes [cm^3] calculated for fully automatic and manually corrected segmentation on expiration B30f scans at baseline, 3, 12 and 24 months. The last column summarizes all time points. All values are separately calculated for the right upper (RUL), middle (RML) and lower lobe (RLL), the left upper lobe (LUL), the lingula (LLi), the left lower lobe (LLL), and also combining left upper lobe and lingula into one lobe (LUL+LLi). Both

methods are compared in accordance with the approach of Bland-Altman giving mean differences (Δ), limits of agreement (LoA) and two regression coefficients (Intercept / Slope and Pearson’s correlation coefficient).
