# Supplementary material for: Validation of automated lobe segmentation on paired inspiratory-expiratory chest CT in 8-14 year-old children with cystic fibrosis
Source: PLoS One. 2018 Apr 9;13(4):e0194557. doi: 10.1371/journal.pone.0194557 (PMC5890971; doi:10.1371/journal.pone.0194557)
Supplement: S8 Table — Lobar volumes [cm3] calculated for fully automatic and manually corrected segmentation on expiration B60f scans at baseline, 3, 12 and 24 months. The last column summarizes all time points. All values are separately calculated for the right upper (RUL), middle (RML) and lower lobe (RLL), the left upper lobe (LUL), the lingula (LLi), the left lower lobe (LLL), and also combining left upper lobe and lingula into one lobe (LUL+LLi). Both methods are compared in accordance with the approach of Bland-Altman giving mean differences (Δ), limits of agreement (LoA) and two regression coefficients (Intercept / Slope and Pearson’s correlation coefficient). (PDF) [file pone.0194557.s020.pdf]

S8 Table. Lobe volume determination on manual and automatic segmentation maps (Expiration B60f scans).

		Baseline	3 months	12 months	24 months	Overall
**RUL**	**manually corrected (mean±sd)**	230±86.22	245.4±94.3	271.73±104.96	310.56±114.78	264.31±102.89
	**automatic (mean±sd)**	228.89±112	249.51±138.37	313.5±162.71	346.75±159.26	284.2±148.66
	**Δ (mean±sd)**	1.11±60.57	-4.1±83.06	-41.77±85.52	-36.19±88.6	-19.9±80.44
	**LoA (2.5% limit, 97.5% limit)**	(-117.61, 119.83)	(-166.91, 158.7)	(-209.38, 125.85)	(-209.85, 137.47)	(-177.55, 137.76)
	**Regr. (Intercept / Slope)**	-79.73 / 1.41	-29.28 / 1.2	-68.56 / 1.39	-19.03 / 1.11	-46.57 / 1.25
	**Regr (Pearson's r)**	0.84	0.81	0.88	0.84	0.86
**RML**	**manually corrected (mean±sd)**	134.68±39.51	134.6±59.44	144.07±49.14	153.96±43.4	141.87±48.05
	**automatic (mean±sd)**	154.91±67.34	123.64±90.66	131.46±44.5	168.03±56.86	144.77±68.42
	**Δ (mean±sd)**	-20.24±59.69	10.96±68.71	12.61±34.88	-14.07±34.35	-2.9±52.78
	**LoA (2.5% limit, 97.5% limit)**	(-137.22, 96.75)	(-123.71, 145.63)	(-55.76, 80.98)	(-81.39, 53.26)	(-106.35, 100.55)
	**Regr. (Intercept / Slope)**	-210.72 / 2.68	-160.25 / 2.18	1.3 / 0.99	-58.98 / 1.41	-88.39 / 1.63
	**Regr (Pearson's r)**	0.48	0.65	0.73	0.8	0.64
**RLL**	**manually corrected (mean±sd)**	338.15±92.55	349.91±146.68	368.35±119.43	426.88±148.03	370.86±130.39
	**automatic (mean±sd)**	309.35±124.27	349.18±174.76	328.55±117.29	366.43±123.65	338.53±135.66
	**Δ (mean±sd)**	28.8±86.71	0.73±87.77	39.8±100.63	60.46±109.79	32.33±96.77
	**LoA (2.5% limit, 97.5% limit)**	(-141.15, 198.74)	(-171.3, 172.75)	(-157.44, 237.03)	(-154.73, 275.64)	(-157.34, 221.99)
	**Regr. (Intercept / Slope)**	-116.94 / 1.24	-93.61 / 1.19	-55.61 / 1.08	-42.27 / 1	-60.05 / 1.08
	**Regr (Pearson's r)**	0.72	0.87	0.64	0.69	0.74
**LUL**	**manually corrected (mean±sd)**	172.68±68.44	171.37±68.27	205.89±86.89	230.07±89.51	194.83±80.76
	**automatic (mean±sd)**	232.02±157.69	185.97±95.79	257.42±120.77	229.93±79.9	225.84±117.22
	**Δ (mean±sd)**	-59.34±136.7	-14.6±90	-51.53±82.52	0.14±74.64	-31.01±99.97
	**LoA (2.5% limit, 97.5% limit)**	(-327.27, 208.58)	(-191, 161.8)	(-213.27, 110.21)	(-146.15, 146.44)	(-226.96, 164.94)
	**Regr. (Intercept / Slope)**	-330.22 / 3.22	-46.38 / 1.39	-19.03 / 1.17	41.71 / 0.84	-40.92 / 1.33
	**Regr (Pearson's r)**	0.5	0.44	0.73	0.62	0.54
**LLi**	**manually corrected (mean±sd)**	95.99±33.01	108.06±49.29	117.96±35.3	123.31±36.67	111±39.73
	**automatic (mean±sd)**	80.64±72.05	106.46±104.34	106.85±63.94	148.98±121.23	110.93±95.66
	**Δ (mean±sd)**	15.35±75.75	1.6±94.11	11.11±50.38	-25.67±102.31	0.08±83.9
	**LoA (2.5% limit, 97.5% limit)**	(-133.12, 163.81)	(-182.85, 186.06)	(-87.64, 109.85)	(-226.2, 174.85)	(-164.36, 164.52)
	**Regr. (Intercept / Slope)**	-383.66 / 4.24	-163.23 / 2.6	-107.6 / 1.83	-99.37 / 1.87	-149.42 / 2.26
	**Regr (Pearson's r)**	0.11	0.43	0.62	0.63	0.49
**LLL**	**manually corrected (mean±sd)**	296.68±93.5	301.04±126.38	328.45±124.97	370.27±128.35	324.04±119.91
	**automatic (mean±sd)**	232.45±122.29	281.1±136.01	272.03±137.58	338.1±134.75	281.06±135.02
	**Δ (mean±sd)**	64.23±96.55	19.95±39.41	56.42±48.63	32.17±49.97	42.98±64.01
	**LoA (2.5% limit, 97.5% limit)**	(-125.01, 253.48)	(-57.3, 97.19)	(-38.89, 151.74)	(-65.78, 130.11)	(-82.47, 168.43)
	**Regr. (Intercept / Slope)**	-111.34 / 1.25	-63.87 / 1.11	-94.44 / 1.15	-57.57 / 1.09	-82.19 / 1.14
	**Regr (Pearson's r)**	0.63	0.96	0.94	0.93	0.88
**LUL+LLi**	**manually corrected (mean±sd)**	268.67±94.49	279.43±114.16	318.03±119.55	353.38±122.37	304.67±115.4
	**automatic (mean±sd)**	312.67±132.63	293.07±120.05	350.03±127.98	378.91±142.08	333.41±132.13
	**Δ (mean±sd)**	-44±95.35	-13.64±33.05	-31.99±39.85	-25.53±40.93	-28.74±57.9
	**LoA (2.5% limit, 97.5% limit)**	(-230.89, 142.9)	(-78.42, 51.14)	(-110.09, 46.1)	(-105.75, 54.69)	(-142.21, 84.74)
	**Regr. (Intercept / Slope)**	-89.92 / 1.5	-12.2 / 1.09	32.2 / 0.96	-27.99 / 1.12	-9.1 / 1.1
	**Regr (Pearson's r)**	0.7	0.96	0.95	0.96	0.9

Lobar volumes [cm^3]calculated for fully automatic and manually corrected segmentation on expiration B60f scans at baseline, 3, 12 and 24 months. The last column summarizes all time points. All values are separately calculated for the right upper (RUL), middle (RML) and lower lobe (RLL), the

left upper lobe (LUL), the lingula (LLi), the left lower lobe (LLL), and also combining left upper lobe and lingula into one lobe (LUL+LLi). Both methods are compared in accordance with the approach of Bland-Altman giving mean differences (Δ), limits of agreement (LoA) and two regression coefficients (Intercept / Slope and Pearson's correlation coefficient).
