# Supplementary material for: Validation of automated lobe segmentation on paired inspiratory-expiratory chest CT in 8-14 year-old children with cystic fibrosis
Source: PLoS One. 2018 Apr 9;13(4):e0194557. doi: 10.1371/journal.pone.0194557 (PMC5890971; doi:10.1371/journal.pone.0194557)
Supplement: S10 Table — Air trapping (E/I MLA) calculated for fully automatic and manually corrected segmentation on B60f scans at baseline, 3, 12 and 24 months. The last column summarizes all time points. All values are separately calculated for the right upper (RUL), middle (RML) and lower lobe (RLL), the left upper lobe (LUL), the lingula (LLi), the left lower lobe (LLL), and also combining left upper lobe and lingula into one lobe (LUL+LLi). Both methods are compared in accordance with the approach of Bland-Altman giving mean differences (Δ), limits of agreement (LoA) and two regression coefficients (Intercept / Slope and Pearson’s correlation coefficient). (PDF) [file pone.0194557.s022.pdf]

S10 Table. E/I MLA determination on manual and automatic segmentation maps (B60f scans).

		Baseline	3 months	12 months	24 months	Overall
**RUL**	**manually corrected (mean±sd)**	0.64±0.09	0.68±0.11	0.67±0.09	0.7±0.09	0.67±0.1
	**automatic (mean±sd)**	0.64±0.09	0.66±0.11	0.67±0.1	0.7±0.09	0.67±0.1
	**Δ (mean±sd)**	0±0.02	0.01±0.02	0±0.01	0±0.01	0±0.02
	**LoA (2.5% limit, 97.5% limit)**	(-0.04, 0.04)	(-0.02, 0.05)	(-0.03, 0.02)	(-0.02, 0.02)	(-0.03, 0.04)
	**Regr. (Intercept / Slope)**	-0.05 / 1.08	0 / 0.99	-0.04 / 1.06	0 / 1	-0.01 / 1.02
	**Regr (Pearson's r)**	0.98	0.99	0.99	0.99	0.99
**RML**	**manually corrected (mean±sd)**	0.74±0.08	0.73±0.09	0.74±0.08	0.74±0.09	0.74±0.08
	**automatic (mean±sd)**	0.73±0.09	0.75±0.12	0.74±0.08	0.75±0.09	0.74±0.1
	**Δ (mean±sd)**	0±0.03	-0.01±0.07	0±0.02	-0.01±0.02	-0.01±0.04
	**LoA (2.5% limit, 97.5% limit)**	(-0.05, 0.06)	(-0.15, 0.12)	(-0.03, 0.03)	(-0.05, 0.03)	(-0.08, 0.07)
	**Regr. (Intercept / Slope)**	-0.12 / 1.16	-0.1 / 1.13	0 / 1	0.02 / 0.98	-0.03 / 1.04
	**Regr (Pearson's r)**	0.95	0.83	0.98	0.97	0.91
**RLL**	**manually corrected (mean±sd)**	0.61±0.11	0.61±0.1	0.62±0.09	0.63±0.09	0.62±0.1
	**automatic (mean±sd)**	0.62±0.11	0.63±0.1	0.64±0.08	0.63±0.08	0.63±0.09
	**Δ (mean±sd)**	-0.02±0.02	-0.02±0.03	-0.02±0.03	0±0.03	-0.02±0.03
	**LoA (2.5% limit, 97.5% limit)**	(-0.06, 0.02)	(-0.09, 0.04)	(-0.09, 0.05)	(-0.07, 0.06)	(-0.08, 0.04)
	**Regr. (Intercept / Slope)**	0.07 / 0.93	0.05 / 0.94	0.11 / 0.86	0.1 / 0.86	0.07 / 0.91
	**Regr (Pearson's r)**	0.98	0.95	0.93	0.94	0.95
**LUL**	**manually corrected (mean±sd)**	0.58±0.1	0.61±0.11	0.63±0.11	0.64±0.1	0.61±0.1
	**automatic (mean±sd)**	0.61±0.1	0.62±0.11	0.64±0.1	0.65±0.09	0.63±0.1
	**Δ (mean±sd)**	-0.02±0.03	-0.01±0.05	-0.01±0.02	-0.01±0.02	-0.01±0.03
	**LoA (2.5% limit, 97.5% limit)**	(-0.08, 0.04)	(-0.1, 0.08)	(-0.05, 0.02)	(-0.04, 0.02)	(-0.07, 0.05)
	**Regr. (Intercept / Slope)**	0.03 / 0.98	0.04 / 0.92	0.06 / 0.93	0.06 / 0.92	0.05 / 0.94
	**Regr (Pearson's r)**	0.96	0.91	0.99	0.99	0.96
**LLi**	**manually corrected (mean±sd)**	0.66±0.11	0.67±0.11	0.71±0.1	0.7±0.08	0.68±0.1
	**automatic (mean±sd)**	0.65±0.12	0.65±0.11	0.7±0.11	0.7±0.09	0.67±0.11
	**Δ (mean±sd)**	0.01±0.09	0.01±0.07	0.01±0.05	0±0.01	0.01±0.06
	**LoA (2.5% limit, 97.5% limit)**	(-0.17, 0.19)	(-0.13, 0.15)	(-0.09, 0.1)	(-0.03, 0.02)	(-0.12, 0.13)
	**Regr. (Intercept / Slope)**	-0.03 / 1.02	0 / 1	-0.06 / 1.1	-0.04 / 1.05	-0.04 / 1.05
	**Regr (Pearson's r)**	0.7	0.77	0.9	0.99	0.82
**LLL**	**manually corrected (mean±sd)**	0.58±0.12	0.58±0.11	0.59±0.11	0.6±0.09	0.59±0.11
	**automatic (mean±sd)**	0.61±0.12	0.6±0.11	0.62±0.1	0.61±0.09	0.61±0.1
	**Δ (mean±sd)**	-0.04±0.03	-0.03±0.02	-0.03±0.02	-0.01±0.02	-0.03±0.03
	**LoA (2.5% limit, 97.5% limit)**	(-0.1, 0.03)	(-0.07, 0.02)	(-0.07, 0.02)	(-0.04, 0.02)	(-0.08, 0.02)
	**Regr. (Intercept / Slope)**	0.04 / 0.98	0.05 / 0.95	0.09 / 0.89	0.06 / 0.92	0.06 / 0.93
	**Regr (Pearson's r)**	0.97	0.98	0.99	0.98	0.97
**LUL+LLi**	**manually corrected (mean±sd)**	0.61±0.1	0.63±0.1	0.65±0.1	0.66±0.09	0.64±0.1
	**automatic (mean±sd)**	0.61±0.1	0.62±0.1	0.65±0.1	0.66±0.09	0.64±0.1
	**Δ (mean±sd)**	0±0.02	0.01±0.02	-0.01±0.02	0±0.01	0±0.02
	**LoA (2.5% limit, 97.5% limit)**	(-0.04, 0.04)	(-0.02, 0.04)	(-0.04, 0.02)	(-0.03, 0.02)	(-0.03, 0.03)
	**Regr. (Intercept / Slope)**	0 / 1	0 / 0.99	-0.02 / 1.04	0.01 / 0.98	0 / 1
	**Regr (Pearson's r)**	0.98	0.99	0.99	0.99	0.99

Air trapping (E/I MLA) calculated for fully automatic and manually corrected segmentation on B60f scans at baseline, 3, 12 and 24 months. The last column summarizes all time points. All values are separately calculated for the right upper (RUL), middle (RML) and lower lobe (RLL), the left upper lobe (LUL), the lingula (LLi), the left lower lobe (LLL), and also combining left upper lobe and lingula into one lobe (LUL+LLi). Both methods are

compared in accordance with the approach of Bland-Altman giving mean differences (Δ), limits of agreement (LoA) and two regression coefficients (Intercept / Slope and Pearson's correlation coefficient).
